# Epigenetically controlled endothelial promyelocytic leukemia drives liver inflammation and fibrosis

**DOI:** 10.1172/JCI196730

**Published:** 2026-03-17

**Authors:** Can Gan, Enjiang Lai, Yang Tai, Shuai Chen, Chong Zhao, Wenting Dai, Zhu Yang, Bei Li, Tian Lan, Yang Xiao, Yangkun Guo, Jiaxin Chen, Bo Wei, Zhaodi Che, Sheng Cao, Mengfei Liu, Frank Tacke, Chengwei Tang, Vijay H. Shah, Haopeng Yu, Fei Wang, Zhiyin Huang, Jinhang Gao

**Affiliations:** 1 Department of Gastroenterology, Lab of Gastroenterology and Hepatology, State Key Laboratory of Biotherapy, West China Hospital, Sichuan University, Chengdu, China.; 2 Department of Gastroenterology and Hepatology, Tongji Hospital, School of Medicine, Tongji University, Shanghai, China.; 3 Department of Biliary Surgery, West China Hospital, Sichuan University, Chengdu, China.; 4 Department of Hepatology and Gastroenterology, Campus Virchow-Klinikum and Campus Charité Mitte, Charité - Universitätsmedizin Berlin, Berlin, Germany.; 5 The First Affiliated Hospital, Key Laboratory of Regenerative Medicine of Ministry of Education, College of Life Science and Technology, Jinan University, Guangzhou, Guangdong, China.; 6 Division of Gastroenterology and Hepatology, Mayo Clinic, Rochester, Minnesota, USA.; 7 Division of Digestive Disease, Internal Medicine, Yale University, New Haven, Connecticut, USA.; 8 Yale Liver Center, New Haven, Connecticut, USA.; 9 West China Biomedical Big Data Center, West China Hospital/West China School of Medicine, Sichuan University, Chengdu, China.; 10 Med-X Center for Informatics, Sichuan University, Chengdu, China.; 11 Division of Gastroenterology, Seventh Affiliated Hospital of Sun Yat-sen University, Shenzhen, China.

**Keywords:** Gastroenterology, Hepatology, Endothelial cells, Epigenetics, Fibrosis

## Abstract

Cellular and molecular heterogeneity in the liver has been increasingly recognized to drive liver fibrosis progression, but the particular events that occur initially in response to liver injury and trigger immune cell recruitment remain unclear. Here, we identify epigenetically aberrant liver sinusoidal endothelial cells (LSECs) as key players in this process. Mechanistically, the epigenetic readers like bromodomain-containing protein 4–dependent (BRD4-dependent) super enhancers (SEs) activate proinflammatory genes, including promyelocytic leukemia (PML). The PML protein, in turn, binds BRD4 and amplifies proinflammatory angiocrine signaling through phase separation–dependent SE activation via PML/BRD4 condensate formation. In mouse models, LSEC-specific depletion of the PML/BRD4 complex mitigates liver inflammation and fibrosis. Single-cell RNA-seq reveals that epigenetically aberrant LSECs exhibit a reprogrammed proinflammatory angiocrine landscape in mouse fibrotic livers. TIMP1^+^ LSECs promote the recruitment of CD63^+^ monocyte–derived macrophages (MoMFs) during liver fibrosis progression. Thereby, PML/BRD4 in LSECs governs inflammatory immune cell recruitment in liver fibrosis. Pharmacological BRD4 inhibition or epigenetic PML-SE repression alleviates liver inflammation and fibrosis. In conclusion, PML/BRD4-mediated SE activation via phase separation drives proinflammatory angiocrine signaling in LSECs, initiating the inflammatory cascade and subsequent immune cell recruitment during liver fibrosis.

## Abstract 2

Phase separation of endothelial PML promotes inflammatory macrophage recruitment in liver fibrosis via the activation of TIMP1-CD63 signaling axis.

## Graphical abstract

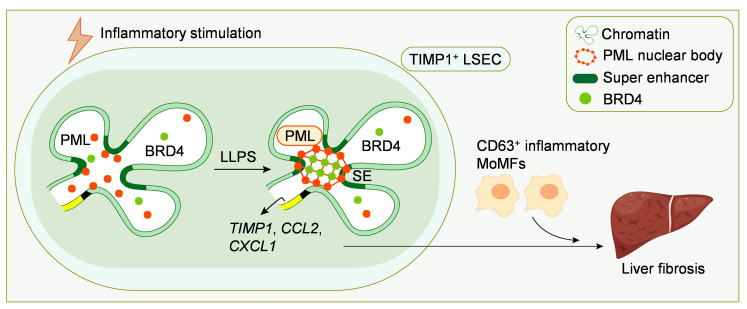

## Introduction

Liver cirrhosis accounts for approximately 2 million deaths annually worldwide (1). Chronic liver injury leads to disrupted liver cell crosstalk, immune cell recruitment, inflammation, and excessive extracellular matrix (ECM) deposition, which eventually progresses to liver fibrosis and cirrhosis (2, 3). Although many efforts have allowed us to decipher the cellular and molecular heterogeneity of liver fibrosis progression, it remains unclear which liver cells initially respond to liver injury and induce immune cell recruitment.

The hepatic sinusoids, lined with liver sinusoidal endothelial cells (LSECs) and surrounded by hepatic stellate cells (HSCs) and Kupffer cells (KCs)/macrophages, provide an ideal microenvironment for liver cell crosstalk (4). As the first line of defense against pathogenic factors during liver injury, LSECs adopt a proinflammatory phenotype and modulate surrounding cells through angiocrine signaling (4). Upon exposure to injury, LSECs transition into a proinflammatory phenotype and influence surrounding cells in an angiocrine signaling–dependent manner. We have previously shown that LSECs induce liver inflammation by recruiting neutrophils or macrophages during alcohol-associated hepatitis (AH) and liver fibrosis, respectively (5, 6). However, the mechanisms underlying LSEC phenotypic switching and the specific contributions of LSEC subtypes to immune cell recruitment in liver fibrosis remain poorly understood.

Epigenetic modifications, including histone acetylation and methylation, can alter gene accessibility and transcription during both normal development and liver diseases (7–9). Our previous findings demonstrated that hepatocyte STING induces NLRP3 expression by increasing histone methylation in the context of liver fibrosis (10). Histone H3 acetylation at lysine 27 (H3K27ac) and epigenetic readers like bromodomain-containing protein 4 (BRD4) are commonly found at promoters and enhancers of active genes (11, 12). These histone marks, epigenetic readers, and transcription factors can be highly enriched within a large cluster of DNA regions termed super enhancers (SEs), which drive rapid gene expression upon stimulation (13, 14). Our previous work demonstrated increased H3K27ac occupancy at SE regions of *CXCLs* in LSECs during AH (5). However, the epigenetic profiles of LSECs during liver fibrosis, particularly the mechanisms governing SE assembly and transcriptional activation, remain unexplored.

In 2017, Richard A. Young et al. proposed a hypothesis that liquid-liquid phase separation (LLPS) contributes to the formation of SEs by enriching transcription factors or coactivators at the chromatin loci of target genes (15, 16). LLPS describes a process where a single homogeneous mixture spontaneously divides into 2 liquid phases through a process involving weak multivalent interactions among molecules (17). Although LLPS typically involves liquid substances, proteins such as SP1, ZMYND8, and YAP, which possess intrinsically disordered regions (IDRs), can undergo LLPS to form intranuclear condensates that assemble transcription factors for SE assembly (18–20). LLPS provides a mechanism for the efficient spontaneous accumulation of transcription factors at SEs. Nevertheless, further exploration is needed to determine whether LLPS is also involved in the epigenetic profiling and SE assembly of LSECs during liver fibrosis.

In this study, we demonstrated that LSEC-derived PML/BRD4 complex drives proinflammatory angiocrine signaling, recruiting inflammatory monocyte-derived macrophages (MoMFs) and neutrophils to exacerbate liver fibrosis. By integrating transcriptomic and epigenomic analyses, we identified SE-induced promyelocytic leukemia (PML) expression in proinflammatory LSECs and successfully epigenetically suppressed PML SE via the CRISPRi-dCas9-KRAB approach to inhibit PML expression and affect angiocrine signaling. We further validated that PML serves as a scaffold for BRD4 accumulation via LLPS, facilitating and amplifying proinflammatory angiocrine signaling. Using single-cell RNA-seq (scRNA-seq) and validation in mouse liver fibrosis models, we confirmed that LSEC-derived PML/BRD4 promotes liver inflammation and fibrosis through activation of tissue inhibitor matrix metalloproteinase 1–positive (TIMP1^+^) LSECs that recruit CD63^+^ MoMFs. Lastly, LSEC-specific depletion, pharmacological inhibition, and/or epigenetic repression of BRD4 and PML, or endothelial *Timp1* knockdown efficiently improve liver inflammation and fibrosis. These findings indicate that phase separation of endothelial PML and subsequent BRD4 accumulation mediates SE activation that drives inflammatory macrophage infiltration during liver fibrosis.

## Results

### Epigenetically aberrant LSECs may facilitate inflammatory immune cell recruitment.

**Figure 1:**
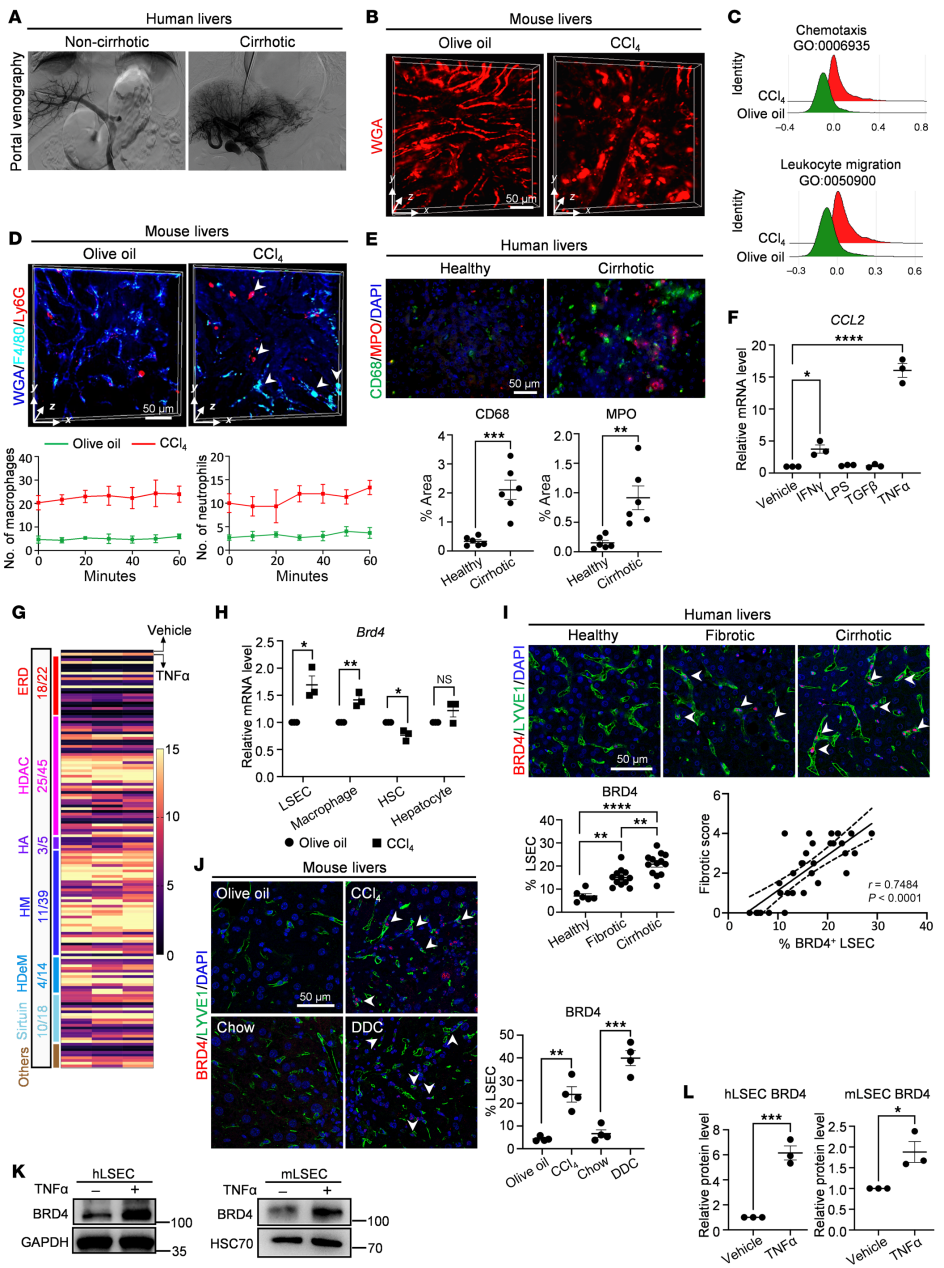
Epigenetically aberrant LSECs are involved in inflammatory immune cell recruitment. (**A**) Portal venography of noncirrhotic and cirrhotic human livers. (**B**) Representative DAOSLIMIT intravital staining of liver sinusoids from mice administered with olive oil or repetitive CCl_4_ injections for 6 weeks (*n* = 3/group). (**C**) Ridge plot of scRNA-seq analysis showing enriched pathways in liver sinusoidal endothelial cells between olive oil and CCl_4_-treated mice (*n* = 3/group). (**D**) Representative DAOSLIMIT liver intravital staining for F4/80 and Ly6G from mice administered with olive oil or CCl_4_ (*n* = 3/group). The quantification is shown in the lower panel. (**E**) Representative immunofluorescence (IF) staining for CD68 and MPO in human livers, comparing healthy and cirrhotic samples (*n* = 6/group). The quantification is shown in the lower panel. (**F**) qPCR analysis of *CCL2* gene in hLSECs treated with a panel of inflammatory stimuli (IFN-γ, LPS, TGF-β, and TNF-α) (*n* = 3, representing 3 independent experiments). (G) qPCR analysis of *CCL2* gene in hLSECs treated with TNF-α and TNF-α plus 152 inhibitors from a histone modification compound library (*n* = 3, representing 3 independent experiments). ERD, epigenetic reader domain; HDAC, Histone Deacetylase; HA, histone acetyltransferase; HM, histone methyltransferase; HDeM, histone demethylase. (**H**) qPCR analysis of the *Brd4* gene in primary liver cells isolated from mice treated with olive oil or CCl_4_ (*n* = 3/group). (**I**) Representative IF staining for BRD4 and LYVE1 in human livers categorized as healthy (*n* = 6), fibrotic (*n* = 12), or cirrhotic (*n* = 13). The arrowhead indicates BRD4^+^ LSECs. The quantification of BRD4^+^ LSECs and the correlation of BRD4^+^ LSECs with liver fibrotic scores are shown in the lower panel. (**J**) Representative IF staining for BRD4 and LYVE1 in mouse livers with olive oil or CCl_4_ administration for 6 weeks and in mouse livers with normal chow or DDC diet for 2 weeks (*n* = 4/group). The quantification is shown in the right panel. (**K**–**L**) Western blot analysis (**K**) and quantification (**L**) of BRD4 protein from hLSECs and isolated mouse primary LSECs treated with vehicle or TNF-α (*n* = 3, representing 3 independent experiments). ****P < 0.0001, ***P < 0.001, **P < 0.01 and *P < 0.05. Data are mean ± SEMs. 2-tailed Student’s *t* test for **E**, **H**, **J**, and **L**. 1-way ANOVA with Tukey’s multiple comparison tests for **F** and **I**. Pearson correlation coefficient analysis for **I**. Scale bars: 50 μm.

Clinical liver angiography revealed increased irregularity and tortuosity of the hepatic vasculature in patients with cirrhosis compared with patients without liver cirrhosis (Figure 1A and Supplemental Video 1; supplemental material available online with this article; https://doi.org/10.1172/JCI196730DS1). Consistently, sinusoidal distortion and congested blood flow in intravital carbon tetrachloride–treated (CCl_4_-treated) mouse fibrotic livers were visualized by digital adaptive optics scanning light-field mutual iterative tomography (DAOSLIMIT) (21), suggesting extensive sinusoidal remodeling during liver fibrosis progression (Figure 1B and Supplemental Video 2). To investigate the mechanism underlying sinusoidal dysfunction, we performed scRNA-seq analysis on normal and fibrotic mouse livers (Supplemental Figure 1A). Gene ontology analysis showed that LSECs express proinflammatory angiocrine genes involved in chemotaxis, leukocyte migration and adhesion, and mononuclear cell proliferation (Figure 1C and Supplemental Figure 1, B and C). To verify these bioinformatics findings, we conducted DAOSLIMIT intravital imaging for F4/80 (a macrophage marker) and MPO (a neutrophil marker) in mouse livers. Compared with that in normal livers, there is a notable increased infiltration of macrophages and neutrophils in the injured livers (Figure 1D and Supplemental Video 3). This increase in macrophage and neutrophil infiltration was also observed in human cirrhotic livers (Figure 1E). These data suggest that LSECs might contribute to the infiltration of inflammatory immune cells by secreting angiocrine factors.

CCL2 is the key chemokine for monocyte recruitment in liver injury (22). CCL2 emerged as the most representative chemokine in dysfunctional LSECs, and was the top-upregulated gene in in vivo murine LSECs (Supplemental Figure 1, D and E). RNA fluorescence in situ hybridization (FISH) analysis confirmed that endothelial CCL2 promoted macrophage infiltration in human and mouse fibrotic livers (Supplemental Figure 1, F and G). To verify which cell-stress conditions lead to LSEC dysfunction, we treated commercial human LSECs (hLSECs) with a panel of common inflammatory stimuli, including IFN-γ, LPS, TGF-β, and TNF-α. The qPCR analysis revealed that endothelial *CCL2* was markedly induced by TNF-α treatment (Figure 1F). To further identify critical signals that regulate LSEC-mediated proinflammatory angiocrine signaling, we treated hLSECs with TNF-α alone or in combination with 152 inhibitors from a histone modification compound library. Inhibitors targeting the epigenetic reader domain showed the highest efficiency (82%) in significantly blocking *CCL2* gene expression, with bromodomain-containing protein 4 (BRD4) inhibitors being the most potent compounds (Figure 1G).

BRD4 is a well-known epigenetic reader that binds to acetylated lysine residues on histones, facilitating the recruitment of transcriptional complexes to active gene loci (23). Our scRNA-seq analysis confirmed that LSECs constituted the most significant proportion of *Brd4*-expressing cells (Supplemental Figure 2A), and endothelial BRD4 expression was increased in CCl_4_-induced liver fibrosis as well as upon TNF-α treatment (Figure 1H and Supplemental Figure 2B). Consistently, published scRNA-seq datasets demonstrated that LSECs are the major cellular source of BRD4 in cirrhotic human and mouse livers (Supplemental Figure 2, C–E). At the protein level, the BRD4 protein level was higher in human cirrhotic and CCl_4_-induced mouse fibrotic livers than in control livers (Supplemental Figure 2F). Colocalization of BRD4 with the LSEC marker LYVE1 identified that BRD4 was abundant in endothelial cells and correlated with liver fibrosis development in human livers. (Figure 1I). Likewise, upregulated endothelial BRD4 expression was observed in CCl_4_- and 3,5-diethoxycarbonyl-1,4-dihydrocollidine–induced (DDC-induced) mouse models of liver fibrosis (Figure 1J), as well as in TNF-α–treated hLSECs and isolated murine LSECs (Figure 1, K and L). These data suggest that endothelial BRD4 is upregulated in fibrotic livers and may contribute to LSEC-mediated inflammatory immune cell recruitment.

### BRD4-dependent SEs promote PML expression.

**Figure 2:**
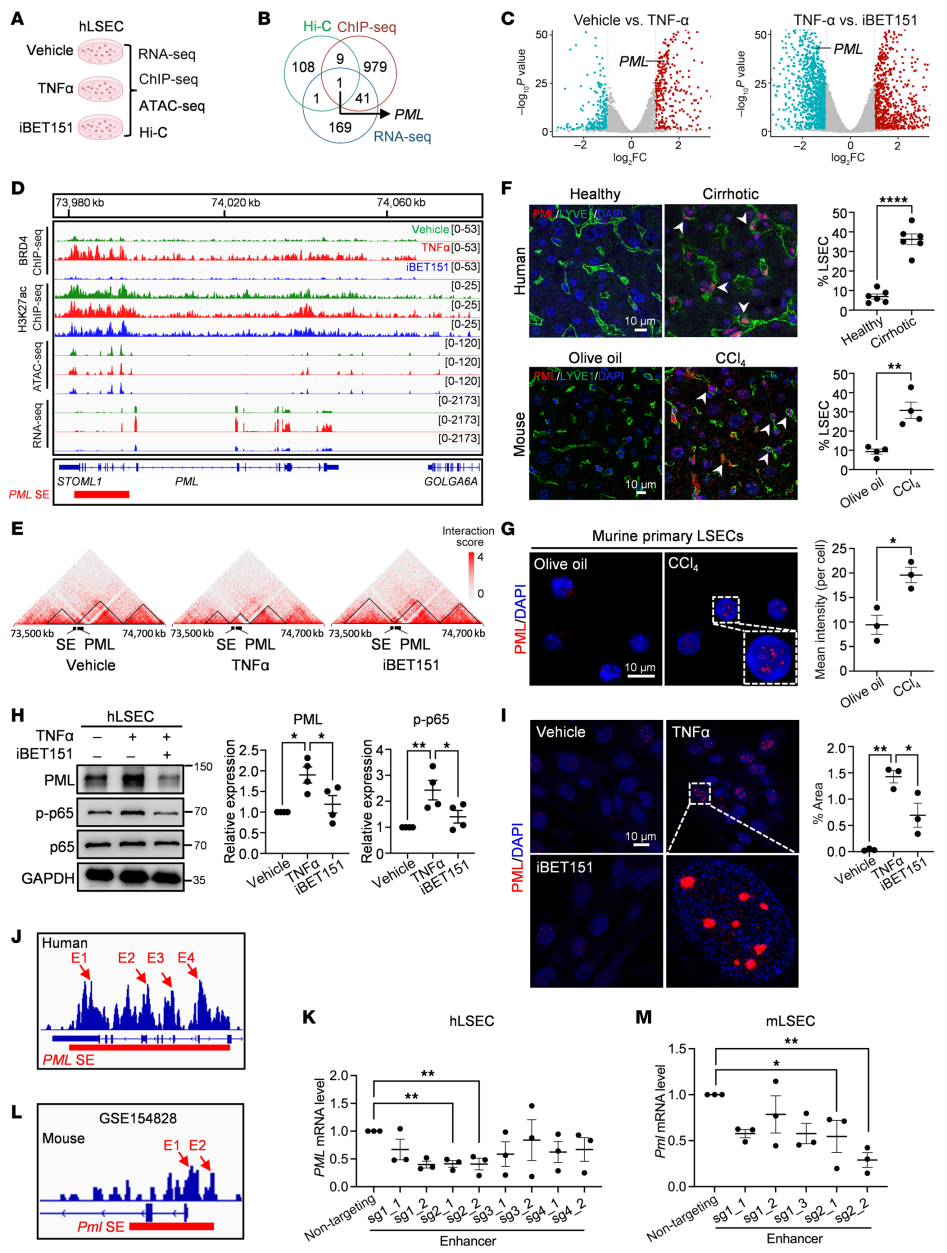
BRD4-dependent super enhancer promotes PML expression. (**A**) Schematic of RNA-seq, ChIP-seq, ATAC-seq, and Hi-C analyses conducted on hLSECs treated with vehicle, TNF-α, or TNF-α plus BRD4 inhibitor (iBET151). (**B**) A Venn diagram illustrating differentially expressed genes (DEGs) identified in RNA-seq, BRD4 ChIP-seq, and Hi-C analyses. (**C**) The volcano plot revealing the upregulated and downregulated DEGs from RNA-seq. (**D**) BRD4 and H3K27ac ChIP-seq and ATAC-seq intensities at the *PML* locus, along with RNA-seq signals for the *PML* expression. The red box indicates *PML* SE. (**E**) Hi-C heatmaps depicting the TAD-reorganization–related activation of the *PML* SE. (**F**) Representative IF images of PML and LYVE1 in human (*n* = 6/group) and mouse (*n* = 4/group) livers. The quantification is shown in the right panel. (**G**) Representative IF images and quantification of PML in isolated LSECs from olive oil and CCl_4_-treated mice (*n* = 3/group). The quantification is shown in the right panel. (**H**–**I**) Western blot analysis (PML and p-p65) and quantification (*n* = 4, representing 4 independent experiments) (**H**) and representative IF staining (PML) and quantification (*n* = 3, representing 3 independent experiments) (**I**) of hLSECs treated with TNF-α and iBET151. (**J**) Schematic of hLSECs H3K27ac ChIP-seq data indicates the human *PML* SE locus with arrows showing the sgRNA targeted sites. (**K**) qPCR analysis of *PML* expression in hLSECs with sgRNA transduction (*n* = 3, representing 3 independent experiments). (**L**) Schematic of the isolated mouse LSECs (mLSECs) H3K27ac ChIP-seq data indicates *Pml* SE locus with arrows showing the sgRNAs targeted sites (GSE154828). (**M**) qPCR analysis of *Pml* expression in mLSECs expressing the dCas9-KRAB protein with sgRNA transfection (*n* = 3, representing 3 independent experiments). ****P < 0.0001, **P < 0.01 and *P < 0.05. Data are means ± SEMs. 2-tailed Student’s *t* test for **F** and **G**. 1-way ANOVA with Tukey’s multiple comparison tests for **H** and **I**. Nested 1-way ANOVA with Dunnett’s test (different targeted sites as the main variable) for **K** and **M**. Scale bars: 10 μm.

To explore how BRD4 contributed to LSEC-mediated proinflammatory angiocrine signaling, we applied multiomics and experimental validation techniques on TNF-α–treated hLSECs. BRD4, together with H3K27ac, is recognized as a marker of putative SEs (5, 24). To uncover the 3D chromatin architecture-related mechanisms of SEs within LSECs and their impacts on genome regulation, we performed RNA-seq, BRD4 and H3K27ac chromatin immunoprecipitation (ChIP) sequencing (ChIP-seq), assay for transposase-accessible chromatin sequencing (ATAC-seq), and high-throughput chromosome conformation capture (Hi-C) analysis of hLSECs treated with vehicle, TNF-α, or iBET151 (TNF-α plus BRD4 inhibitor iBET151, Figure 2A). RNA-seq identified 213 BRD4-driven differentially expressed genes (DEGs), which were upregulated by TNF-α and downregulated by iBET151 (Supplemental Figure 3A). Similarly, 943 BRD4-driven SEs were identified in BRD4 ChIP-seq data of the TNF-α group (Supplemental Figure 3B). The increase in BRD4 and H3K27ac ChIP-seq signal intensity was observed on chromatins, especially on putative SEs in TNF-α–treated hLSECs, which diminished after iBET151 treatment (Supplemental Figure 3, C and D). In addition, ATAC-seq analysis revealed 3,954 BRD4-driven chromatin regions, all of which displayed increased chromatin accessibility after TNF-α treatment but were blocked by iBET151 (Supplemental Figure 3, E–G). Integrated analysis of RNA-seq, ChIP-seq, and Hi-C identified one overlapped DEG, promyelocytic leukemia (*PML*) (Figure 2, B and C).

PML is a multifunctional protein associated with DNA repair, apoptosis, and gene expression (25). The increased activity of *PML* putative SE, as well as the open *PML* gene loci indicated by BRD4 and H3K27ac ChIP-seq and ATAC-seq, were inhibited by the BRD4 inhibitor iBET151 (Figure 2D). Hi-C analysis revealed a high level of interaction between the SE and *PML* gene within a single topologically associating domain (TAD) in TNF-α–treated hLSECs, facilitating SE induction of PML expression. However, this interaction was separated into 2 TADs by iBET151, indicating that the TAD conformation manipulates SE activity (Figure 2E). In line with our findings, the activity of *PML* SE was shown to be regulated in a BRD4-dependent manner, as demonstrated in published BRD4 and p65 ChIP-seq analysis on TNF-α–treated HUVECs using the classical BRD4 inhibitor JQ1 (Supplemental Figure 4A). Indeed, increased PML protein levels were observed in both human and mouse LSECs from fibrotic livers, as shown by colocalization of PML and LYVE1 (Figure 2F). As determined by immunofluorescence (IF) staining of PML in primary isolated LSECs, endothelial PML was also increased in CCl_4_-induced fibrotic livers (Figure 2G). Likewise, analysis of public datasets revealed that LSECs were the main cellular source of PML in the human and mouse livers (Supplemental Figure 4, B and C). Among the 4 inflammatory stimuli, hLSECs treated with TNF-α exhibited significantly elevated PML expression (Supplemental Figure 4D), which was reversed by the BRD4 inhibitor iBET151 (Figure 2, H and I). PML protein oligomerizes and forms shell-like nuclear bodies (NBs) that contain numerous client proteins (25). This structural organization is disrupted upon generation of the PML/RAR-α fusion oncoprotein (26). Interestingly, the PML NBs appeared as dot-shaped spherical structures without PML/RAR-α induction (Figure 2, G and I, and Supplemental Figure 4E).

To further verify that suppression of PML SE activity inhibits *PML* gene expression, we utilized the CRISPRi-dCas9-KRAB epigenome system (11, 27). We designed multiple sgRNAs targeting the *PML* enhancers and transduced the sgRNA lentivirus along with dCas9-KRAB lentivirus into hLSECs (Figure 2J). Among these, sgRNAs targeting Enhancer 2 region (sg2_1 and sg2_2) showed the highest efficiency in repressing *PML* gene expression without affecting adjacent genes *STOML1* and *GOLGA6A* (Figure 2K and Supplemental Figure 4F). We also transfected sgRNAs targeting murine *Pml* enhancers, based on publicly available ChIP-seq datasets, into isolated mouse LSECs (mLSECs) expressing the dCas9-KRAB protein from *dCas9-KRAB*/*Cdh5*^CreERT2^ mice (Figure 2L). Notably, sgRNAs targeting Enhancer 2 region (sg2_1 and sg 2_2) were identified as the most effective in disturbing *Pml* SE and silencing *Pml* gene expression (Figure 2M). These data suggest that epigenetically manipulating BRD4-mediated *PML* SE activity can effectively suppress PML expression.

### PML directly binds with BRD4 in the nucleus.

**Figure 3:**
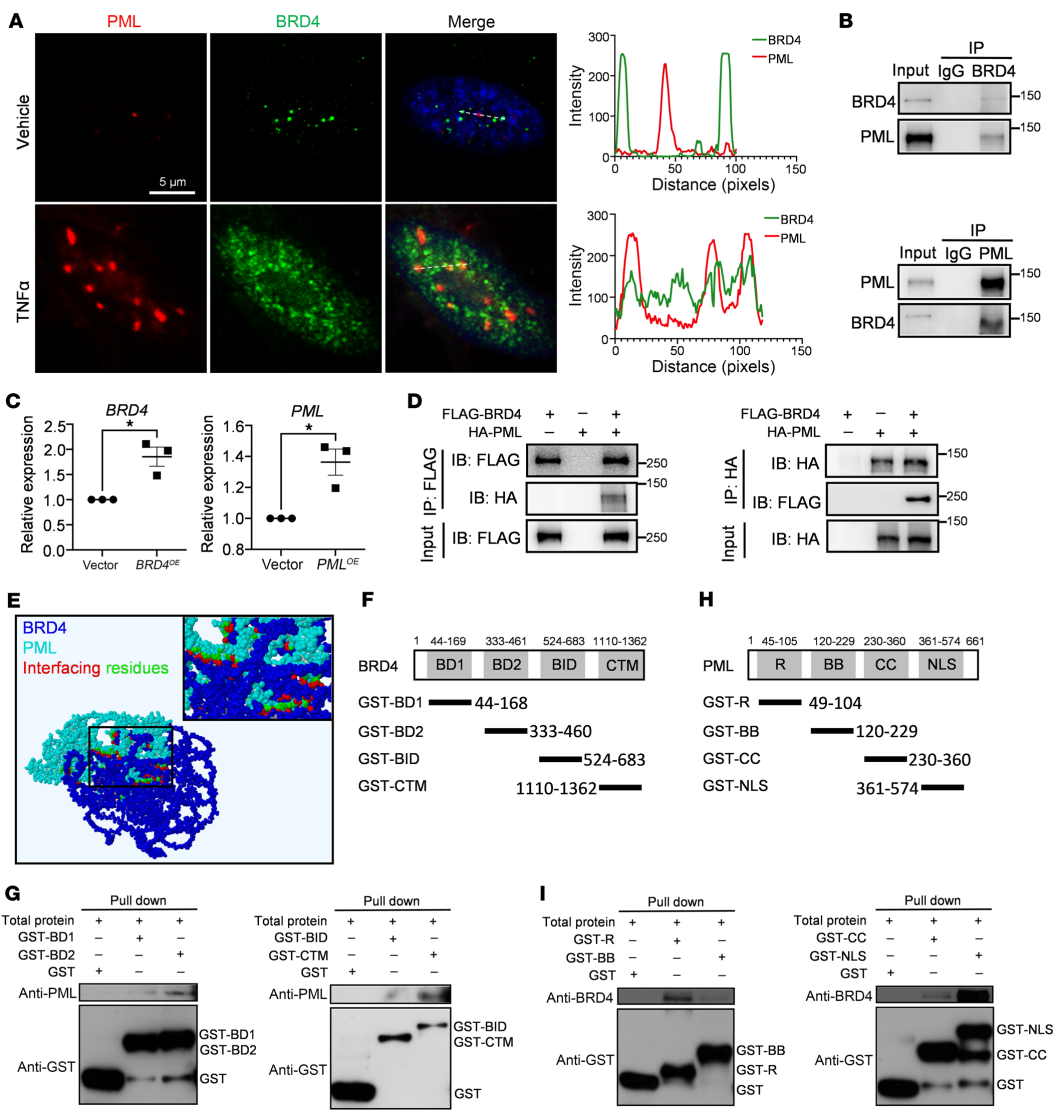
PML directly binds with BRD4 in the nucleus. (**A**) Representative IF staining and colocalization analysis (white line) of PML and BRD4 in hLSECs treated with vehicle or TNF-α. Scale bar: 5 μm. (**B**) Endogenous BRD4 and PML proteins were induced by TNF-α treatment on hLSECs. Reciprocal immunoprecipitation (IP) and immunoblotting (IB) analyses show that BRD4 binds with PML. (**C**) Lentivirus was transduced into hLSECs to overexpress BRD4 or PML, and qPCR was used to analyze BRD4 or PML expression (*n* = 3, representing 3 independent experiments). (**D**) HEK293T cells were transfected with either FLAG-tagged BRD4 or HA-tagged HA individually, or in combination. Reciprocal IP and IB analyses reveal the association between exogenous BRD4 and PML. (**E**) Molecular docking of PML-BRD4 proteins by the GRAMM-X platform and visualization by the PDBePISA platform. (**F**) Schematic diagram of the BRD4 domains and BRD4 GST fusion protein constructs. (**G**) The GST pull-down assay shows the interaction of BRD4 domains with the PML protein. (**H**) Schematic diagram of the PML domains and PML GST fusion protein constructs. (**I**) The GST pull-down assay shows the interaction of PML domains with BRD4 protein. *P < 0.05. Data are means ± SEMs. 2-tailed Student’s *t* test for **C**.

Given the BRD4-driven SE-induced PML expression and their localization in the nucleus of LSECs, we next explored the association between PML and BRD4. Interestingly, TNF-α treatment induced endogenous PML and BRD4 proteins to colocalize, with BRD4 found within the PML NBs in hLSECs (Figure 3A). Additionally, reciprocal immunoprecipitation (IP) followed by immunoblotting confirmed the efficient coimmunoprecipitation of endogenous PML with BRD4 (Figure 3B). Furthermore, an IP assay using exogenously overexpressed PML and BRD4 via lentiviral transduction in hLSECs or plasmid transfection of HA-PML and FLAG-BRD4 in HEK293T cells also demonstrated a direct interaction between BRD4 and PML (Figure 3, C and D, and Supplemental Figure 5A). These results suggested the existence of a PML/BRD4 complex in the nucleus.

Next, we hypothesized that PML physically binds with BRD4. To this end, we performed molecular docking analysis and found direct binding between PML and BRD4 by hydrogen bonds and salt bridges connecting interfacing residues (Figure 3E and Supplemental Figure 5B). Then, a GST pull-down assay using various GST/BRD4 fusion protein constructs purified from bacteria identified the BD2 domain (spanning amino acids 333–460), as well as the CTM domain (spanning amino acids 1,110–1,362) of BRD4, directly interacting with PML (Figure 3, F and G, and Supplemental Figure 5C). Next, we tested the interaction domains of PML for BRD4 binding using various GST/PML fusion protein constructs (Figure 3H). The R domain (spanning amino acids 49–104) and NLS domain (spanning amino acids 361–574) of PML were found to interact with BRD4, with the latter exhibiting a stronger interaction (Figure 3I and Supplemental Figure 5D). These results were consistent with the molecular docking predictions, which suggest the NLS domain of the PML protein binds with the CTM domain of the BRD4 protein. In summary, the above data demonstrate the physical binding of the PML and BRD4 proteins in the nucleus.

### PML accumulates BRD4 protein via phase separation to amplify proinflammatory gene expression.

**Figure 4:**
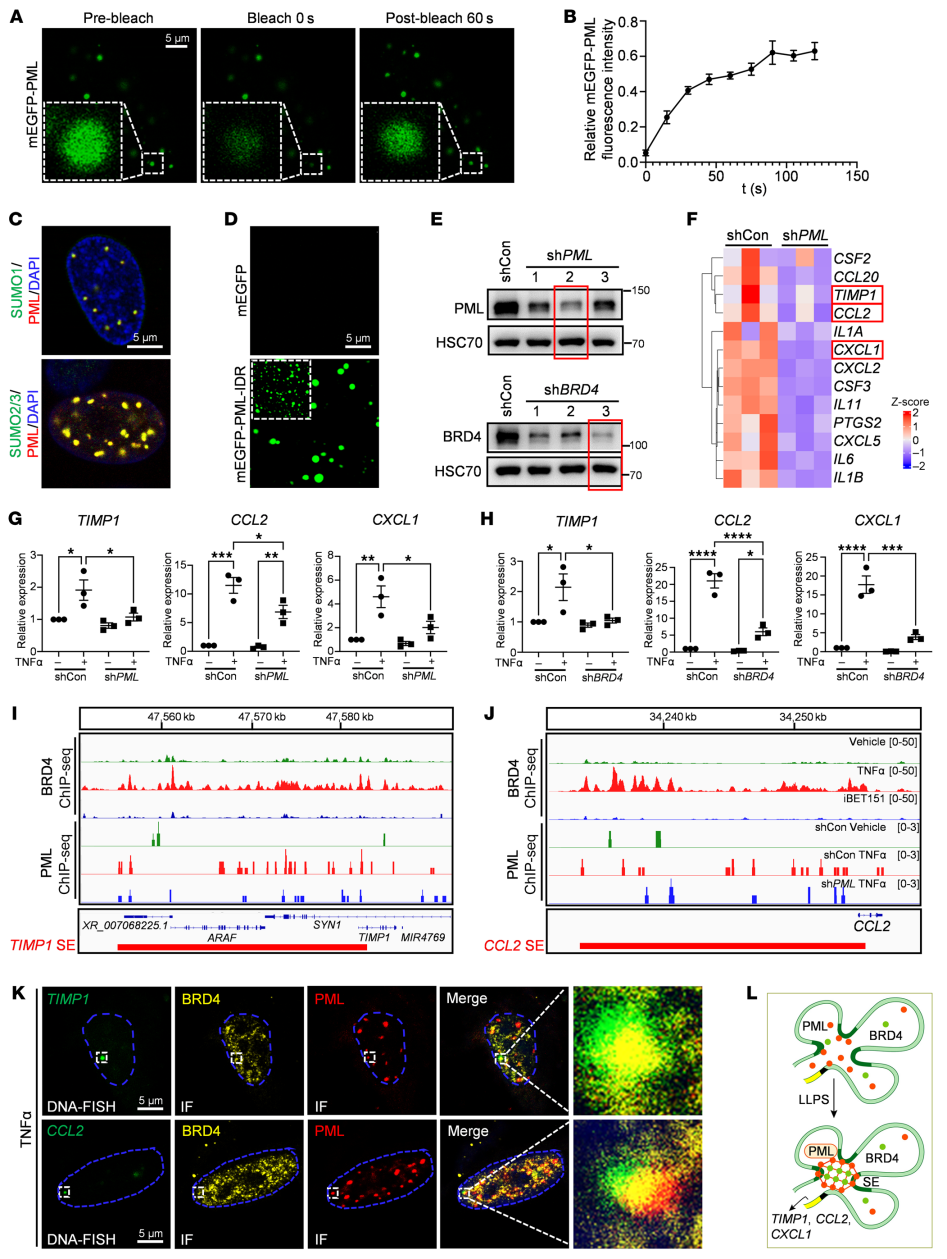
PML accumulates BRD4 protein via phase separation to facilitate proinflammatory gene expression. (**A**) Representative images of the FRAP experiment with mEGFP-PML–engineered hLSECs to validate the LLPS of PML. The white dashed box highlights the punctum undergoing targeted bleaching. (**B**) Quantification of FRAP data for mEGFP-PML puncta. The bleaching event starts at *t* = 0 s. (**C**) Representative costaining of SUMO1 and SUMO2/3 with PML in hLSECs treated with TNF-α. (**D**) Representative images of the droplet formation assay to validate the LLPS of PML. mEGFP-PML-IDR protein was added to the lipid droplet buffer to a final concentration of 20 μM. (**E**) Western blot analysis of PML and BRD4. Three lentiviruses were transduced into hLSECs to knock down the expression of PML or BRD4, respectively. Given that Sh*PML*#2 and sh*BRD4* #3 showed the highest efficiency, these lentiviruses were used for subsequent experiments. (**F**) RNA-seq heatmap of hLSECs treated with TNF-α plus shControl (shCon) and TNF-α plus *PML* knockdown (sh*PML*). (**G**–**H**) qPCR analysis of *CCL2*, *CXCL1* and *TIMP1* genes. ShCon, sh*PML*#2 (**G**), or sh*BRD4* #3 (**H**) were transduced into hLSECs before TNF-α treatment. (*n* = 3, representing 3 independent experiments). (**I** and **J**) BRD4 and PML ChIP-seq signals for *TIMP1* SE (**I**) and *CCL2* SE (**J**). The red boxes indicate SEs of *TIMP1* and *CCL2*, respectively. (**K**) IF staining for PML, BRD4, and DNA-FISH for the *TIMP1* or *CCL2* gene in hLSECs after TNF-α treatment. (**L**) Proposed working model: PML selectively accumulates BRD4 in conjunction with the SE loci of *TIMP1*, *CCL2*, and *CXCL1*, amplifying gene expression through a phase separation-dependent manner. ****P < 0.0001, ***P < 0.001, **P < 0.01, and *P < 0.05. Data are means ± SEMs. 1-way ANOVA with Tukey’s multiple comparison tests was used. Scale bars: 5 μm.

LLPS can facilitate the assembly of SEs by concentrating transcription factors, cofactors, and chromatin regulators to SEs (15). Given our observations of PML protein forming puncta within the nucleus (Figure 2, G and I, and Figure 3A), we employed fluorescence recovery after photobleaching (FRAP) and lipid droplet formation assays to ascertain whether these puncta exhibited the features of LLPS (28). In the FRAP assay on live hLSECs endogenously expressing tagged mEGFP-PML, the mEGFP-PML puncta demonstrated fluorescence recovery within seconds after photobleaching (Figure 4, A and B). These findings suggest that the PML protein can form liquid-like condensates. PML NBs are known to require SUMOylation for their assembly and stability (25). Consistent with this finding, PML harbors conserved SUMOylation motifs (Supplemental Figure 6A). Furthermore, PML indeed underwent SUMOylation in hLSECs upon TNF-α treatment, as shown by the colocalization of SUMO1 and SUMO2/3 with PML (Figure 4C and Supplemental Figure 6, B and C). In contrast, BRD4 acetylation is not involved in this LLPS process. Although BRD4 is predicted to contain putative acetylation sites (Supplemental Figure 6D), its acetylation signals were not significantly altered by TNF-α (Supplemental Figure 6E).

Intrinsically disordered regions (IDRs) of proteins are involved in condensate formation to create LLPS droplets (29). Interestingly, the PML protein is predicted to contain conserved IDRs within its NLS domain in both human and mouse species (Supplemental Figure 7A), and the protein binding regions of PML are predicted to overlap with the PML IDR (Supplemental Figure 7B). The IDR lipid droplet formation assay showed the formation of spherical droplets in purified recombinant mEGFP IDR fusion proteins (mEGFP-PML-IDR), but not in the mEGFP control protein (Figure 4D). These results demonstrated that PML forms puncta in an LLPS manner.

To examine whether the PML/BRD4 complex promotes gene expression in an LLPS-induced SE manner, we performed RNA-seq and ChIP-seq analysis on hLSECs following *PML* knockdown and TNF-α treatment. *PML* knockdown resulted in decreased expression of proinflammatory genes, such as tissue inhibitor matrix metalloproteinase 1 (*TIMP1*), *CCL2*, and *CXCL1* (Figure 4, E and F). These results were further verified by qPCR analysis of hLSECs (Figure 4G). Increased expression of *CCL2*, *CXCL1*, and *TIMP1* induced by TNF-α was also inhibited by *BRD4* knockdown (Figure 4, E and H). Moreover, the increased BRD4 and PML signal intensities observed in ChIP-seq analyses following TNF-α treatment indicate the presence of putative SEs associated with *TIMP1, CCL2*, and *CXCL1* (Figure 4, I and J, and Supplemental Figure 8, A and B). Hi-C analysis revealed increased interchromosomal interactions between *TIMP1* and *CCL2* genes, as well as between *TIMP1* and *CXCL1* genes in TNF-α–treated hLSECs (Supplemental Figure 8C). Given the phase-separation capability of PML and its regulatory role on these genes, we speculate that PML drives the expression of these genes by incorporating the loci of SEs into the PML NBs via phase separation. To further determine the regulatory role of the PML/BRD4 complex on proinflammatory genes, we conducted IF staining for PML and BRD4 proteins together with DNA-FISH for *TIMP1* and *CCL2* genes in hLSECs with vehicle or TNF-α treatment. We found that PML/BRD4 complex puncta overlapped the DNA-FISH foci in TNF-α–treated hLSECs, suggesting the presence of PML/BRD4 condensates at the SEs of these proinflammatory genes (Figure 4K and Supplemental Figure 8, D and E). In summary, PML accumulates BRD4 protein via phase separation to facilitate proinflammatory gene expression (Figure 4L).

### LSEC-specific depletion of PML/BRD4 complex in mice mitigates liver inflammation and fibrosis.

Given the upregulation of LSEC-derived PML and BRD4 and their regulatory roles in proinflammatory gene expression, we next investigated the biological functions of endothelial PML and BRD4 in liver fibrosis. Initially, we employed a conditional LSEC-specific *Brd4* knockout (*Brd4*^ΔLSEC^) and *Pml* knockout (*Pml*^ΔLSEC^) mouse model using an inducible *Cdh5*^CreERT2^ system, which did not cause detectable tissue abnormalities (Supplemental Figure 9, A–C, and Supplemental Figure 10, A–C). qPCR analysis of isolated murine LSECs revealed efficient knockdown of *Brd4* in *Brd4*^ΔLSEC^ mice and *Pml* in *Pml*^ΔLSEC^ mice compared with the corresponding controls following tamoxifen administration (Supplemental Figure 9D and Supplemental Figure 10D).

**Figure 5:**
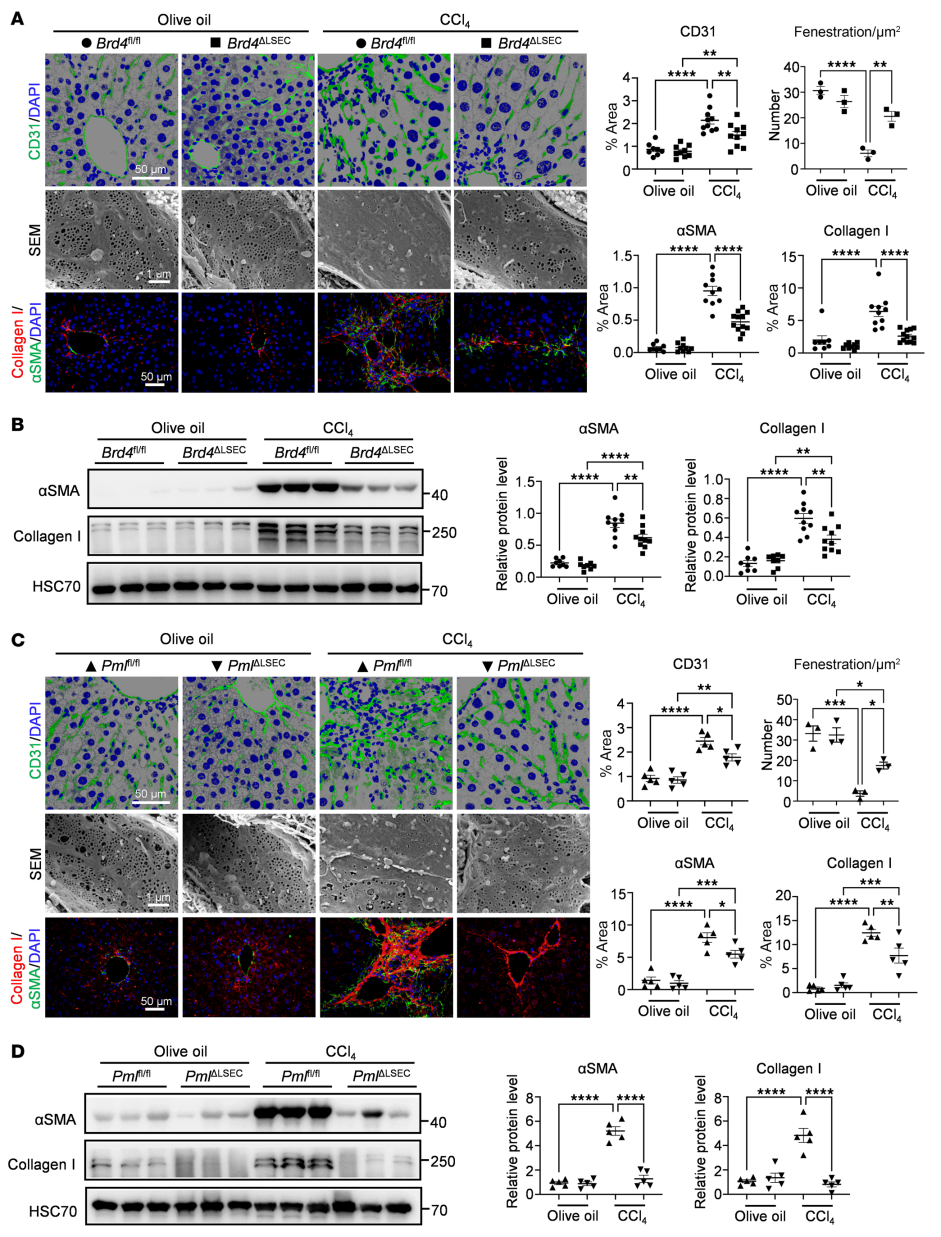
LSEC-specific PML/BRD4 complex depletion in mice attenuates liver inflammation and fibrosis. (**A**-**B**) *Brd4*^fl/fl^ and *Brd4*^ΔLSEC^ mice were intraperitoneally (i.p.) injected with olive oil (*n* = 8–9/group) or CCl_4_ (*n* = 10–12/group) for 6 weeks. Representative IF staining and quantifications for CD31 and collagen I/αSMA in liver sections, as well as scanning electron microscopy (SEM) images of the liver sinusoids with the quantification of LSEC fenestration (**A**). Western blot analysis and quantification of collagen I and αSMA in mouse livers (**B**). (**C** and **D**) *Pml*^fl/fl^ and *Pml*^ΔLSEC^ mice were i.p. injected with olive oil (*n* = 5/group) or CCl_4_ (*n* = 5/group) for 6 weeks. Representative IF staining and quantifications for CD31 and collagen I/αSMA in liver sections, as well as SEM images of the liver sinusoids with the quantification of LSEC fenestration (**C**). Western blot analysis and quantification of collagen I and αSMA in mouse livers (**D**). ****P < 0.0001, ***P < 0.001, **P < 0.01 and *P < 0.05. Data are means ± SEMs. 1-way ANOVA with Tukey’s multiple comparison tests was used. Scale bars (A and C): 50 μm (top and bottom rows); 1 μm (middle row).

First, we assessed the role of LSEC-derived BRD4 and PML in liver fibrosis using a 6-week CCl_4_ treatment protocol. Compared with *Brd4*^fl/fl^ mice, decreased expression of BRD4 in LSECs was confirmed at both the tissue and isolated cell levels in *Brd4*^ΔLSEC^ mice subjected to CCl_4_ treatment (Supplemental Figure 9, E and F). Interestingly, angiogenesis with new tortuous vasculature was revealed in CCl_4_-treated livers, as shown by CD31 staining with 3D reconstruction, accompanied by sinusoidal capillarization with the disappearance of LSEC fenestration, as determined by scanning electron microscopy (SEM). In contrast, LSEC-derived BRD4 depletion reversed abnormal angiogenesis and LSEC defenestration (Figure 5A and Supplemental Figure 9G). Remarkably, *Brd4*^ΔLSEC^ mice exhibited reduced inflammatory cell recruitment, as shown by H&E staining and IF staining for F4/80 and MPO (Supplemental Figure 9G). We also observed a remarkable decrease in HSC activation as shown by IF staining and Western blot (WB) analysis for α-SMA, along with reduced collagen deposition evidenced by IF staining and WB analysis for collagen I and Sirius red staining in *Brd4*^ΔLSEC^ mice treated with CCl_4_ (Figure 5, A and B, and Supplemental Figure 9G). Consistently, the endothelial PML, the severity of abnormal liver angiogenesis and LSEC defenestration, the inflammatory environment, and collagen deposition were also remarkably reduced in mice with LSEC-specific ablation of PML (Figure 5, C and D, and Supplemental Figure 10, E–G). These findings imply that the ablation of endothelial BRD4 and PML impedes the progression of inflammatory immune cell recruitment and liver fibrosis.

To ascertain that the observed effects of the LSEC-derived PML/BRD4 complex were not model-specific, we applied a DDC-diet mouse model of liver injury for 2 weeks. As expected, DDC-fed mice presented increased macrophage and neutrophil recruitment, HSC activation, and collagen deposition in the portal vein area (Supplemental Figure 11, A–D). Nonetheless, LSEC-specific BRD4 depletion significantly attenuated inflammatory immune cell accumulation, HSC activation, and collagen deposition (Supplemental Figure 11, A–D). Consistently, decreased macrophage and neutrophil infiltration and HSC activation were revealed in *Pml*^ΔLSEC^ mice fed a DDC diet (Supplemental Figure 12, A–D). In summary, our findings suggest that the PML/BRD4 complex in LSECs promotes angiogenesis, immune cell infiltration, and collagen deposition, thus leading to liver inflammation and fibrosis.

### Epigenetically aberrant LSECs exhibit a reprogrammed proinflammatory angiocrine landscape in mouse fibrotic liver.

**Figure 6:**
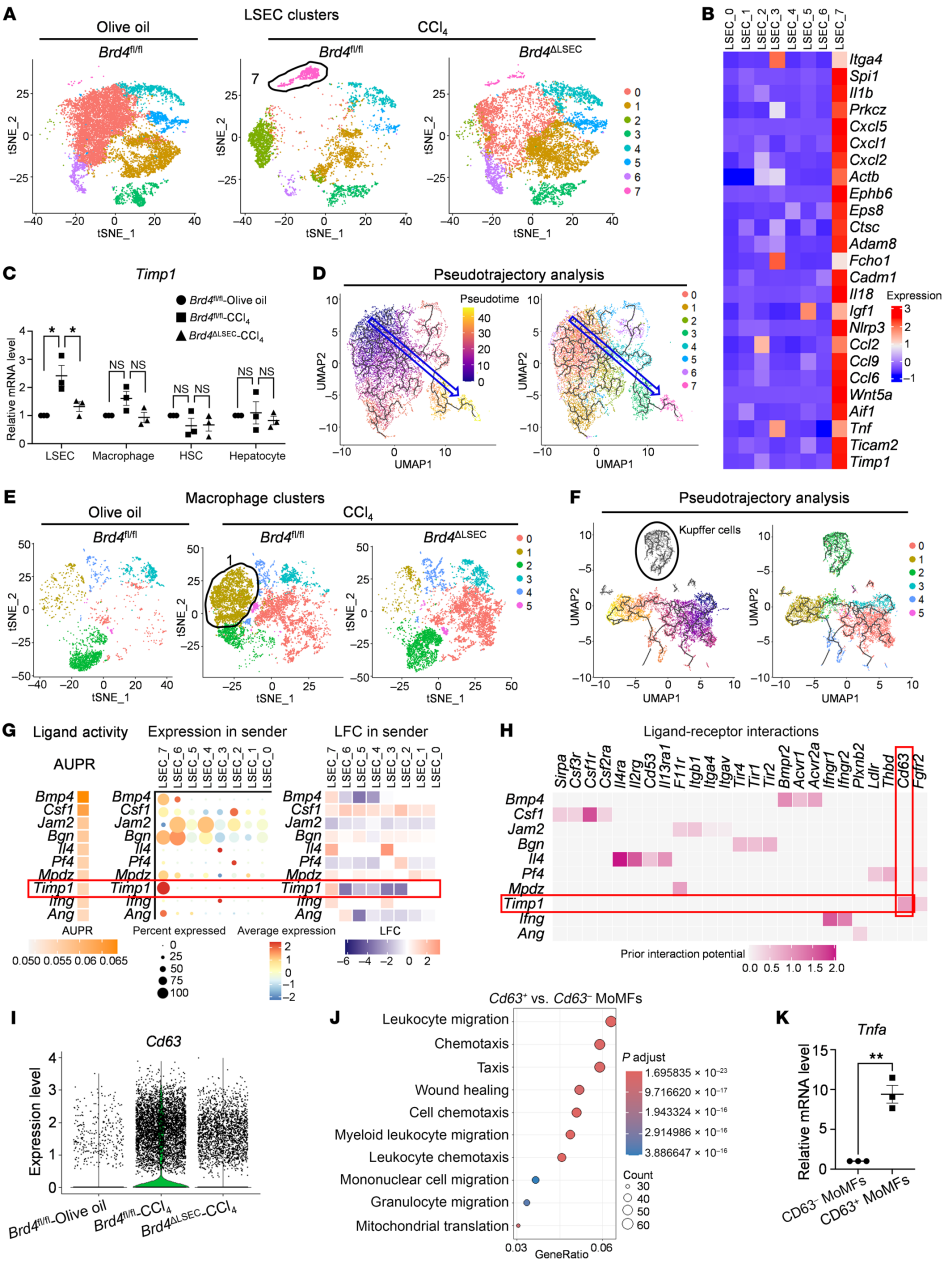
scRNA-seq analysis reveals pathological communications between LSECs and MoMFs in mouse fibrotic livers. scRNA-seq analysis was performed on normal (olive oil) and fibrotic (CCl_4_) mouse livers with or without LSEC-specific *Brd4* depletion (*n* = 3/group). (**A**) t-Distributed stochastic neighbor embedding (t-SNE) analysis illustrating 8 different LSEC clusters in the livers. (**B**) Heatmap of representative endothelial angiocrine genes in the 8 LSEC clusters. (**C**) qPCR analysis of *Timp1* gene in primary liver cells isolated from *Brd4*^fl/fl^ and *Brd4*^ΔLSEC^ mice with olive oil or CCl_4_ treatment (*n* = 3/group). (**D**) Pseudotrajectory analysis of LSEC lineages in normal and fibrotic mouse livers using Monocle3 (left) and visualization of clusters onto the pseudotime map (right). (**E**) t-SNE analysis identified 6 different clusters of macrophages in the livers. (**F**) Pseudotrajectory analysis of macrophage lineages in normal and fibrotic mouse livers using Monocle3 (left) and visualization of clusters onto the pseudotime map (right). (**G**) Ligand activity and expression analysis of LSECs. AUPR, area under the precision-recall curve; LFC, log–fold change. (**H**) Ligand-receptor interaction analysis of LSECs and macrophages. (**I**) *Cd63* expression in macrophages among the 3 groups. (**J**) Enriched pathways in *Cd63*^+^ MoMFs compared with *Cd63*^–^ MoMFs. (**K**) qPCR analysis of *Tnfa* gene in isolated CD63^–^ MoMFs and CD63^+^ MoMFs from CCl_4_-treated mice. **P < 0.01 and *P < 0.05 Data are means ± SEMs. 1-way ANOVA with Tukey’s multiple comparison tests for **C**. 2-tailed Student’s *t* test for **K**.

LSECs secrete angiocrine factors that interact with surrounding cells under both healthy and diseased states (4). To explore how epigenetically aberrant LSECs influence the recruitment of inflammatory immune cells, we performed scRNA-seq analysis using nonparenchymal cells (NPCs) from normal and CCl_4_-induced fibrotic livers, with and without LSEC-specific *Brd4* depletion. We identified 8 clusters, including LSECs, HSCs, KCs, MoMFs, neutrophils, and other immune cells utilizing conserved gene markers (Supplemental Figure 13, A–C). To determine which LSEC subsets exhibit SE-dependent gene expression, we performed subgroup analysis on LSECs and found 8 clusters based on marker genes (Figure 6A and Supplemental Figure 13D). Among these clusters, cluster 7 (LSEC_7) was significantly expanded after CCl_4_ treatment. Gene ontology analysis revealed that LSEC_7 expressed more genes associated with leukocyte activation, migration, adhesion, and chemotaxis. In contrast, LSEC_0 and LSEC_1 were enriched for genes involved in homeostatic functions, such as regulation of hydrolase activity, cell growth, and vascular maintenance, whereas LSEC_2 appeared to represent an intermediate state between LSEC_0/1 and LSEC_7 (Supplemental Figure 13, E–H). Furthermore, we identified LSEC_7, which was predominantly expanded in CCl_4_-treated LSECs, served as the major cellular source of proinflammatory angiocrine factors, such as *Ccl2* and *Timp1* (Figure 6B). These upregulated genes were notably mitigated in a BRD4-dependent manner, as evidenced by isolated LSECs, scRNA-seq, and ChIP-seq, as well as RNA-FISH data (Figure 6C and Supplemental Figure 14, A–D). Pseudotemporal trajectory analysis of LSEC subsets further demonstrated a transition from normal to proinflammatory gene expression during the progression of liver fibrosis, pinpointing LSEC_7 as a key contributor to LSEC inflammation, with LSEC_2 representing a translational intermediate (Figure 6, B and D). This proinflammatory transition in LSECs was normalized by LSEC-specific *Brd4* depletion (Figure 6A). Consistent with these findings, scRNA-seq of *Pml*^ΔLSEC^ mouse livers revealed a reduction in the LSEC subset (LSEC_3), which exclusively expressed *Timp1* alongside genes associated with leukocyte migration, adhesion, and chemotaxis (Supplemental Figure 15, A–G). These angiocrine factors, such as *Timp1* and *Ccl2,* were decreased by endothelial PML depletion, as evidenced by scRNA-seq and isolated LSECs data (Supplemental Figure 15, G and H). In summary, epigenetically aberrant angiocrine signaling might play a significant role in promoting liver fibrosis.

### Endothelial BRD4/PML contributes to inflammatory CD63^+^ MoMFs recruitment.

As macrophages become prominent immune cells during the development of liver fibrosis (Supplemental Figure 13A), we further analyzed macrophages at the single-cell level and subclassified them into 6 different clusters. t-SNE analysis revealed that cluster 1, which was significantly enriched in fibrotic livers, was diminished in livers from *Brd4*^ΔLSEC^ mice (Figure 6E and Supplemental Figure 16A). Cluster 1 exhibited an inflammatory profile characterized by involvement in myeloid leukocyte migration, activation and chemotaxis, and cytokine-mediated signaling pathways and regulation of inflammatory response (Supplemental Figure 16, B and C). The pseudotemporal trajectory analysis revealed the dynamic transitions between macrophage subpopulations during liver fibrosis. Cluster 2 was identified as KCs, the liver resident macrophages, while other clusters were classified as MoMFs based on distinct differentiation paths and marker gene expression (Figure 6F and Supplemental Figure 16B). Notably, cluster 1 (MoMF_1) emerged as a terminally differentiated, highly inflammatory population (Figure 6F and Supplemental Figure 16C).

Given the increase in proinflammatory angiocrine factors from LSECs (Figure 6B) and the remarkable rise in macrophages, we analyzed communications between LSECs and macrophages via ligand-receptor interaction analysis (Figure 6, G and H). We first identified the top 10 ligands based on their activity and expression levels in LSECs (Figure 6G). Among these, *Timp1*, shown to be regulated by PML/BRD4 complex in vitro (Figure 4, G and H), was exclusively expressed in LSEC_7 and displayed high activity (Figure 6G). Additionally, we observed a strong interaction between *Timp1* in LSECs and *Cd63* in macrophages (Figure 6H). Interestingly, *Cd63* was uniquely upregulated in MoMF_1 (Supplemental Figure 16B) in an endothelial BRD4-dependent manner (Figure 6I). Compared with *Cd63*^–^ MoMFs, *Cd63*^+^ MoMFs were enriched for genes associated with leukocyte migration and chemotaxis (Figure 6J). Furthermore, the inflammatory phenotype of CD63^+^ MoMFs was confirmed in isolated MoMFs from CCl_4_-treated mice, with increased *Tnfa* expression in CD63^+^ MoMFs compared with CD63^–^ MoMFs (Figure 6K). Moreover, CD63^+^ MoMFs were observed to be specifically located in mouse fibrotic livers (Supplemental Figure 16D). Although the total macrophage population did not show a significant increase in scRNA-seq data from *Pml*^fl/fl^ and *Pml*^ΔLSEC^ mouse livers with CCl_4_ treatment, the expanded inflammatory CD63^+^ MoMFs were also decreased in *Pml*^ΔLSEC^ group (Supplemental Figure 17, A–E). This finding highlights the heterogeneity of macrophages during liver fibrosis and the role of endothelial BRD4/PML complex in inflammatory macrophage recruitment.

### TIMP1^+^ LSECs recruit CD63^+^ MoMFs during the progression of liver fibrosis.

**Figure 7:**
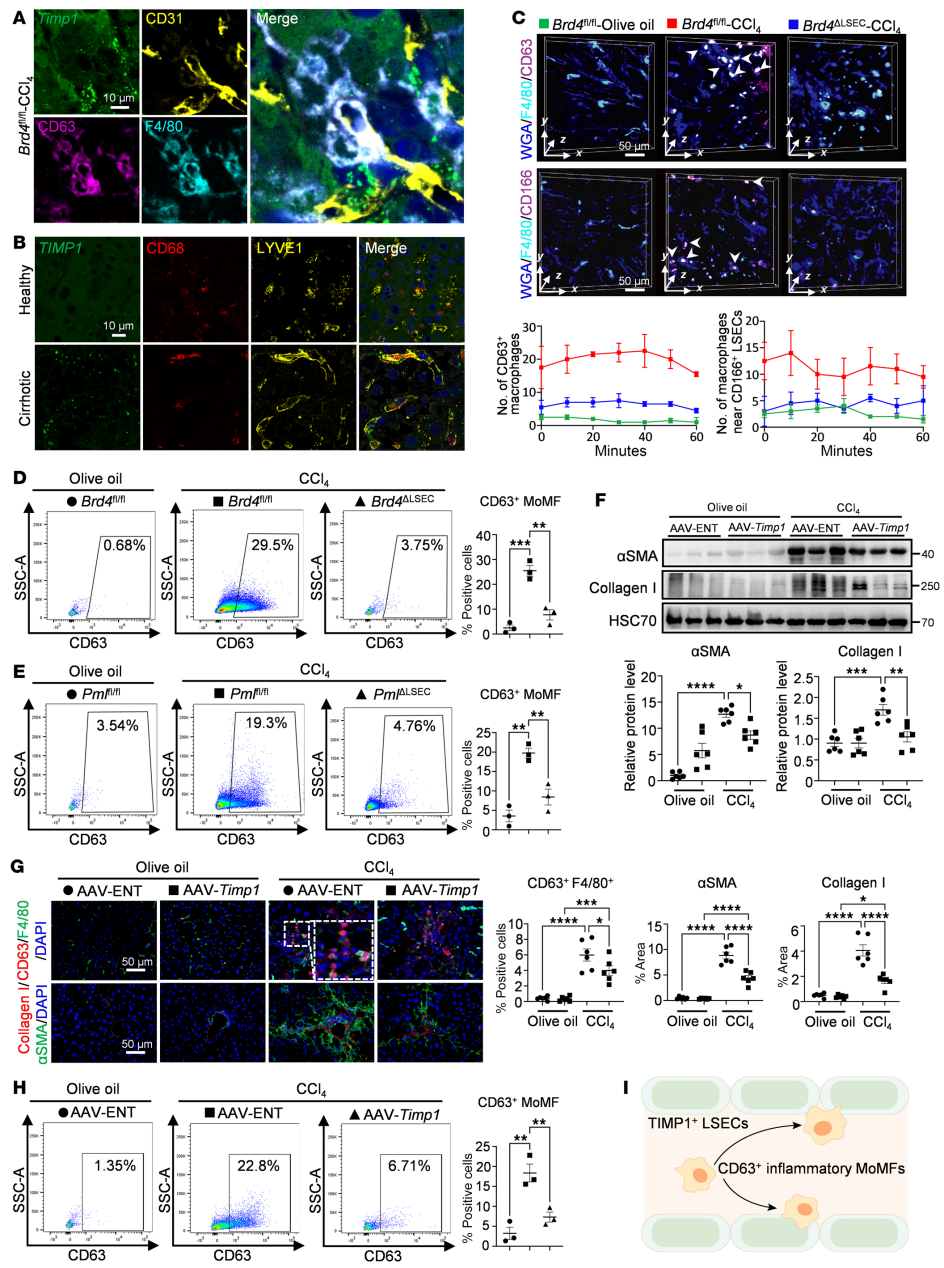
TIMP1^+^ LSECs recruit CD63^+^ MoMFs during the progression of liver fibrosis. (**A**) RNA-FISH analysis of *Timp1* probe and proteins (CD31, F4/80, and CD63) in liver sections from *Brd4*^fl/fl^ mice with CCl_4_ treatment (*n* = 3/group). (**B**) RNA-FISH analysis of *TIMP1* probe and proteins (LYVE1 and CD68) in liver sections from people who were healthy controls and patients with cirrhosis. (**C**) Representative DAOSLIMIT liver intravital staining for WGA/F4/80/CD166 and WGA/F4/80/CD63 from mice administered with olive oil or CCl_4_ with and without LSEC-specific *Brd4* depletion (*n* = 2/group). The quantification is shown in the lower panel. (**D**) Flow cytometry analysis and quantification of CD63^+^ MoMFs from *Brd4*^fl/fl^ and *Brd4*^ΔLSEC^ mice with olive oil or CCl_4_ treatment (*n* = 3/group). (**E**) Flow cytometry analysis and quantification of CD63^+^ MoMFs from *Pml*^fl/fl^ and *Pml*^ΔLSEC^ mice with olive oil or CCl_4_ treatment (*n* = 3/group). (**F**–**H**) WT mice were pretreated with either AAV-ENT or AAV-*Timp1* via i.v. injection prior to i.p. injection of olive oil or CCl_4_. Western blot analysis and quantification of collagen I and αSMA in mouse livers (**F**). Representative IF staining and quantification of CD63/F4/80 and collagen I/αSMA in mouse liver sections (**G**). Flow cytometry analysis and quantification of CD63^+^ MoMFs from AAV-ENT and AAV-*Timp1* mice with olive oil or CCl_4_ treatment (*n* = 3/group) (**H**). (**I**) Proposed working model: TIMP^+^ LSECs recruit CD63^+^ MoMF infiltration in the liver during the progression of liver fibrosis. ****P < 0.0001, ***P < 0.001, **P < 0.01, and *P < 0.05. Data are means ± SEMs; 1-way ANOVA with Tukey’s multiple comparison tests on **D–H**. Scale bars: 10 μm (**A** and **B**); 50 μm (**C** and **G**).

To further investigate the biological importance of TIMP1^+^ LSECs in recruiting CD63^+^ MoMFs, we conducted RNA-FISH analysis on mouse fibrotic liver samples. The mRNA level of *Timp1* in LSECs was significantly increased in fibrotic livers, and CD63^+^ MoMFs were in close proximity to *Timp1*^+^ LSECs. However, *Timp1*^+^ in LSECs, as well as CD63 in MoMFs, were remarkably decreased in *Brd4*^ΔLSEC^ mouse livers (Figure 7A and Supplemental Figure 18A). Consistently, *TIMP1^+^* LSECs were also found in close proximity to macrophages in human cirrhotic livers (Figure 7B). To directly visualize the dynamic process by which TIMP1^+^ LSECs recruit CD63^+^ MoMFs in vivo, we performed DAOSLIMIT imaging on mouse fibrotic livers. Since TIMP1 is an angiocrine factor secreted by LSECs, we utilized CD166, an LSEC_7-specific cell membrane marker in LSECs, instead of TIMP1 for visualization (Supplemental Figure 18B). DAOSLIMIT imaging revealed an increase in CD166^+^ LSECs, as well as the hepatic recruitment of CD63^+^ MoMFs in close proximity to CD166^+^ LSECs in CCl_4_-induced fibrotic livers (Figure 7C and Supplemental Videos 4 and 5). These observations were substantially diminished in the livers of LSEC-specific *Brd4*-depletion mice (Figure 7C and Supplemental Videos 4 and 5). Consistently, macrophages, especially CD63^+^ MoMFs, accumulated near CD166^+^ LSECs in the human cirrhotic livers (Supplemental Figure 18, C and D). Flow cytometry analysis further confirmed that CD63^+^ MoMFs were accumulated in the liver during fibrosis progression, and this recruitment was significantly reversed upon LSEC-specific depletion of the BRD4 or PML (Figure 7, D and E, and Supplemental Figure 18E). The molecular docking analysis revealed direct stable physical interactions between TIMP1 and CD63 mediated by hydrogen bonds (Supplemental Figure 18, F and G). In summary, TIMP1^+^ LSECs recruit CD63^+^ MoMFs during the progression of liver fibrosis.

### LSEC-knockdown of TIMP1 attenuates liver inflammation and fibrosis via reducing CD63^+^ MoMFs.

To further demonstrate the role of LSEC-derived TIMP1 in liver fibrosis development in vivo, we delivered adeno-associated virus serotype 2 (AAV2)-ENT-*Timp1*(AAV-*Timp1*) to WT mice to selectively knockdown the expression of LSEC *Timp1* followed by CCl_4_ administration (Supplemental Figure 19A). The vector AAV-*Timp1* is constructed on an AAV2 recombinant backbone and engineered with an endothelial-specific TIE1 promoter to drive the expression of both GFP and shRNA (30). Both AAV-*Timp1* and AAV-ENT predominantly target LSECs (Supplemental Figure 19B). Among the constructs tested, AAV-*Timp1*-1 showed the highest efficiency, achieving approximately 50% knockdown compared with the AAV-ENT control (Supplemental Figure 19C). We then induced liver fibrosis in AAV-ENT and AAV-*Timp1*-1 delivered mice by administering CCl_4_. As expected, increased tortuous vessels, CD63^+^ MoMFs, and collagen deposition with increased endothelial *Timp1* were improved by blockade of LSEC-*Timp1* (Figure 7, F and G, and Supplemental Figure 19D–G), indicating the proinflammatory and profibrotic effects of LSEC-derived TIMP1 in the setting of liver fibrosis.

The proinflammatory and profibrotic role of LSEC-derived TIMP1 was then validated. Flow cytometry analysis of fibrotic livers showed increased monocyte infiltration and differentiation into MoMFs, approximately 16.7% of which were CD63^+^ MoMFs. However, LSEC *Timp1* knockdown did not attenuate this monocyte infiltration (Figure 7H and Supplemental Figure 20A). Mechanistically, this effect was specifically dependent on the TIMP1-CD63 interaction and not secondary to alterations in canonical chemokine signaling in LSECs (Supplemental Figure 20, B and C). These data indicate that LSEC TIMP1 promotes liver inflammation by recruiting CD63^+^ MoMFs (Figure 7I).

To determine whether the profibrotic effect of endothelial TIMP1 is direct or macrophage mediated, we first examined its role in monocyte/macrophage function using the THP1 human monocytic model. THP1 cells differentiated into a CD63^+^ phenotype and exhibited enhanced migration when treated with conditional media or cocultured with TNF-α–treated hLSECs, whereas these effects were blocked by *TIMP1* knockdown in hLSECs (Supplemental Figure 20, D and E). Furthermore, conditional media from LPS-treated THP1 cells, a phenotype of CD63^+^ macrophages, induced HSC activation and collagen I expression (Supplemental Figure 20, F and G), suggesting a macrophage-dependent profibrotic mechanism. To test whether LSEC TIMP1 can also directly activate HSCs, we co-cultured murine primary HSCs with LSECs isolated from CCl_4_-treated AAV-ENT or AAV-*Timp1* mice. HSC activation was markedly enhanced when cocultured with CCl_4_-treated LSECs, an effect also observed in the LSEC *Timp1* knockdown group (Supplemental Figure 20H). These results indicate that LSEC-derived TIMP1 activates HSCs through CD63^+^ MoMF-mediated indirect pathways. Interestingly, HSC activation was blocked by LSEC BRD4 depletion or inhibition (Supplemental Figure 20, I and J). In summary, the above data elucidate the function of LSEC-derived TIMP1 in the recruitment of CD63^+^ MoMFs and the subsequent activation of HSCs, eventually aggravating liver fibrosis.

### Pharmacological BRD4 inhibition alleviates liver inflammation and fibrosis.

**Figure 8:**
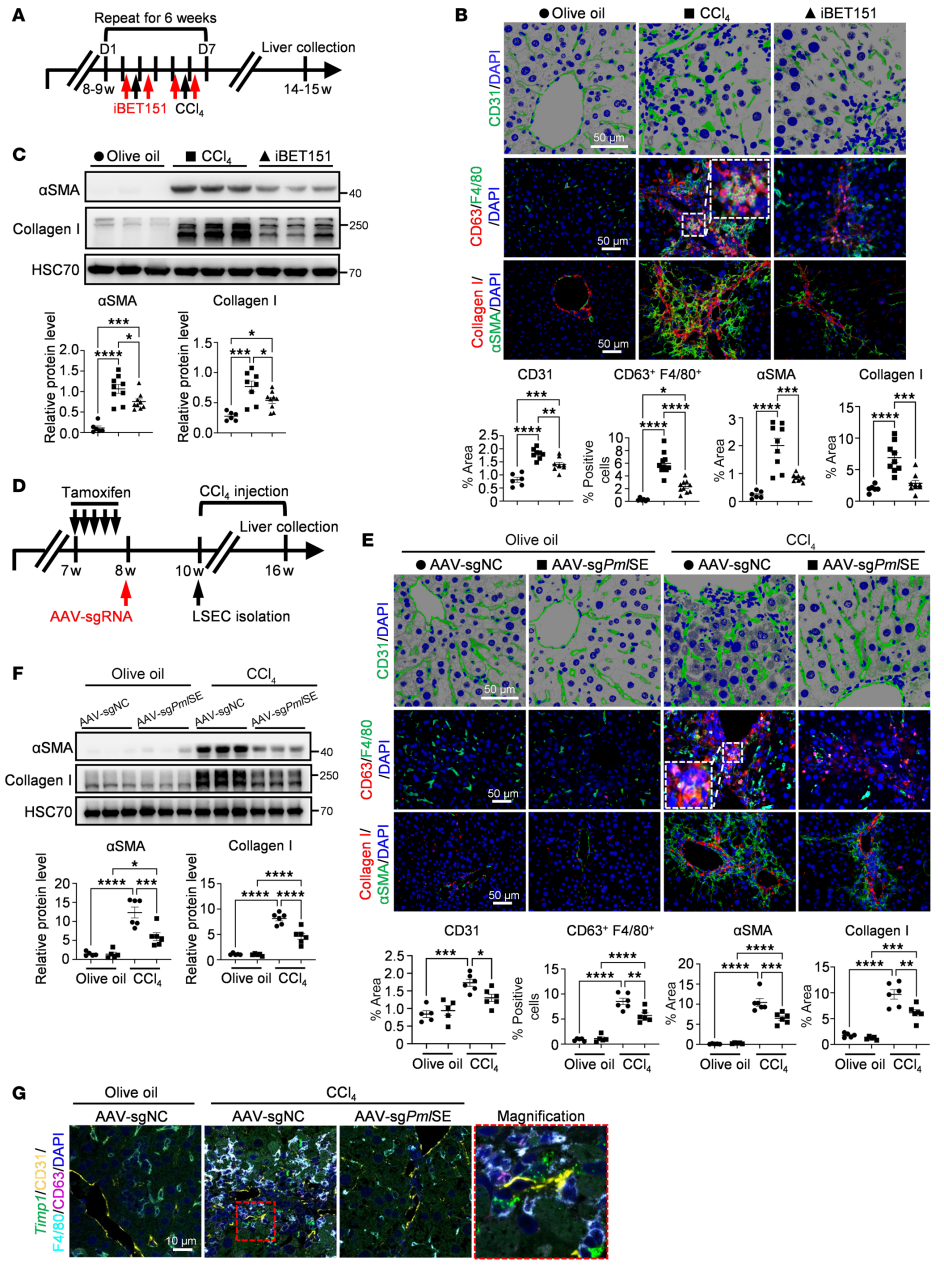
Blockade of BRD4/PML complex ameliorates liver inflammation and fibrosis. (**A**–**C**) Schematic of the mouse liver fibrotic model and iBET151 treatment. Mice were i.p. injected with olive oil (*n* = 6), CCl_4_ (*n* = 9), or CCl_4_ plus the iBET151 compound (*n* = 9) for 6 weeks (**A**). Representative IF staining and quantification of CD31, CD63/F4/80, and collagen I/αSMA in mouse livers (**B**). Western blot analysis and quantification of collagen I, αSMA on mouse livers (**C**). (**D**–**G**) Schematic of AAV-sgRNA delivery to *dCas9-KRAB*/*Cdh5*^CreERT2^ mice and the liver fibrosis model (*n* = 5/group, olive oil) (*n* = 6/group, CCl_4_) (**D**). Representative IF staining and quantification of CD31, CD63/F4/80, and collagen I/αSMA in mouse livers (**E**). Western blot analysis and quantification of collagen I, αSMA in mouse livers (**F**). (**G**) RNA-FISH analysis of *Timp1* probe and proteins (CD31, F4/80, and CD63) in mouse liver sections (*n* = 3/group). ****P < 0.0001, ***P < 0.001, **P < 0.01 and *P < 0.05. Data are means ± SEMs. 1-way ANOVA with Tukey’s multiple comparison tests on **B**, **C**, **E**, and **F**. Scale bars: 10 μm (**G**); 50 μm (**B** and **E**).

To evaluate the potential of BRD4 inhibition as a therapeutic approach for treating liver fibrosis, we administered the BRD4 inhibitor iBET151 to mice during the 6-week CCl_4_ injection (Figure 8A). Consistent with our previous study (5), iBET151 showed no signs of organ toxicity and was well tolerated (Supplemental Figure 21, A and B). Consistent with observations in *Brd4*^ΔLSEC^ mice, iBET151 administration resulted in decreased abnormal angiogenesis, CD63^+^ MoMFs, and neutrophil infiltration in mice subjected to CCl_4_ treatment. A significant decrease in HSC activation and collagen deposition at both the histologic and molecular levels was also noted following iBET151 treatment (Figure 8, B and C, and Supplemental Figure 21C). These in vivo data suggest that pharmacological inhibition of BRD4 diminishes SE activity, consequently reducing hepatic inflammation and fibrosis.

### Epigenetic repression of PML SE relieves liver inflammation and fibrosis.

We previously demonstrated the efficient disturbance of PML SE activity via the dCas9-KRAB approach in vitro and highlighted that PML phase separation plays an important role in gene regulation. The next goal was to investigate the biological function of endothelial PML SE activity in vivo. Given the efficacy of sg2_2 on the activity of PML SE, we administered AAV-sg*PML* (sg2_2) systemically to 8-week-old *dCas9-KRAB*/*Cdh5*^CreERT2^ mice via tail vein injection (Figure 8D). We validated the efficiency of AAV delivery and subsequent repression of *Pml* mRNA expression in isolated LSECs 2 weeks after AAV delivery (Supplemental Figure 22, A and B). Next, a mouse liver fibrosis model was conducted via CCl_4_ administration. A significant decrease in tortuous angiogenesis, CD63^+^ MoMFs and neutrophil recruitment, HSC activation, and collagen deposition was shown in AAV-sg*PML* mice subjected to CCl_4_ treatment (Figure 8, E and F, and Supplemental Figure 22C). Moreover, RNA-FISH analysis revealed that LSEC-derived TIMP1 attracted CD63^+^ MoMF infiltration in fibrotic livers from AAV-sgNC mice, but this effect was not observed in AAV-sg*PML* mice (Figure 8G and Supplemental Figure 22D). These data demonstrate that inhibiting the SE activity of LSEC-derived PML via epigenetic manipulation silences PML expression and mitigates liver inflammation and fibrosis.

## Discussion

It is well known that immune cells accumulate during liver fibrosis (31), but the mechanisms behind their recruitment remain unclear. Angiocrine signaling is critical for endothelial cells to maintain liver homeostasis and influences disease progression (4). This study revealed that the PML/BRD4 complex in LSECs drives proinflammatory angiocrine signaling, inducing a TIMP1^+^ proinflammatory LSEC phenotypic transition and recruiting CD63^+^ macrophages to exacerbate liver fibrosis. Mechanistically, PML/BRD4 orchestrates this process through SE- and phase separation-dependent transcription. These findings not only clarify the role of LSECs in immune cell recruitment but also highlight novel therapeutic targets for liver fibrosis.

Current therapies for liver fibrosis primarily focus on hepatocytes, HSCs, and immune cells, with no approved clinical treatments for liver cirrhosis (32). However, vascular remodeling, marked by irregular and tortuous sinusoids, is increasingly recognized as a driver of fibrosis (33–35). Given their role as the liver’s first line of defense (36), LSECs may represent a novel therapeutic target for liver fibrosis. This work revealed that LSEC-specific disruption of BRD4, PML, TIMP1, and epigenetic repression of PML SE attenuate liver fibrosis, mirroring the benefits observed with endothelial Notch, p300, and HK2 modulation (6, 37, 38). Inflammation is typically associated with the development of liver fibrosis, and antiinflammatory therapies targeting specific inflammatory mediators, such as CCL2, IL-1β, and TNF-α, show potential for therapeutic applications (6, 10, 39, 40). In the liver, LSECs are crucial regulators of the intrahepatic proinflammatory microenvironment via angiocrine signaling (4). Therefore, since BRD4 and PML function as transcriptional coactivators, their depletion selectively reduces the production of proinflammatory cytokines and the recruitment of immune cells. Consistently, LSEC-specific depletion or pharmacological inhibition of HDAC2 and DNMT1 alleviated liver fibrosis by epigenetically reprogramming vascular adaptation and decreasing profibrotic Th17 cells (33). These results suggest that epigenetic modulation of LSECs could be a novel therapeutic strategy for liver fibrosis.

Hepatic macrophages are important to activate HSCs in liver fibrosis (32, 41). Our previous study demonstrated that LSECs produce CXCL1 and CCL2 to attract neutrophils and macrophages in AH and liver fibrosis, respectively (5, 6, 37). As for the heterogeneity of these macrophages, which subtype responds to LSEC injury and how they are recruited to the liver remains unclear. In the current study, a novel proinflammatory subset of TIMP1^+^ LSECs was identified to critically mediate the recruitment of inflammatory CD63^+^ macrophages derived from monocytes. Similar to the heterogeneity of macrophages, published scRNA-seq data on LSECs also uncovered the portal-central axis zonation of LSECs and Oit3 as a marker gene for LSECs (42, 43). However, the heterogeneity of LSECs and their interaction with macrophages in the context of liver fibrosis remain poorly understood. The TIMP1^+^ LSEC subset is mainly distributed to the periportal fibrotic regions and is the major cellular source of inflammatory angiocrine factors, such as CCL2, TIMP1, IL-1β, and CXCL1. In this study, TIMP1 depletion in LSECs was sufficient to reduce inflammatory CD63^+^ macrophage infiltration and mitigate liver inflammation and fibrosis. In alignment with these findings, systemic TIMP1 silencing attenuates macrophage recruitment and liver fibrosis (44). These data establish TIMP1^+^ LSECs as key regulators of macrophage-driven fibrosis and refine our understanding of LSEC heterogeneity in disease.

LSECs undergo phenotypic shifts during liver injury (4, 11), but the epigenetic mechanisms governing these changes are poorly understood. BRD4 serves as a reader of acetylated histones in the transcription of profibrotic genes (45). Here, we identified that BRD4 induces proinflammatory signaling in TIMP1^+^ LSECs by binding to SEs of genes like *TIMP1*, *IL-1β*, *CCL2*, and *CXCL1*. Consistently, BRD4 promotes *Il1b* transcription through enhancer-dependent cis-regulatory pathways in CX3CR1^+^ macrophages during cardiac fibrosis (46). SE activity is regulated by 3D chromatin interactions organized into TADs. Notably, BRD4 physically interacts with PML at SEs within the TADs of *TIMP1* and *CCL2*, suggesting a cooperative role of PML and BRD4 in transcriptional activation in LSECs during liver fibrosis. PML polymerizes to form shell-like membrane-free structures known as nuclear bodies (NBs), which exhibit high functional heterogeneity by recruiting diverse client proteins (25). Although the importance of PML in acute promyelocytic leukemia has been well documented previously (47, 48), the source and role of PML in the liver remain largely unexplored. In this study, enlarged PML NBs in fibrotic LSECs were enriched with BRD4 at the SEs of *TIMP1* and *CCL2*. The mechanism by which PML cooperates with BRD4 remains unclear. Proteins harboring one or more IDRs might undergo LLPS, while the interaction site of PML with BRD4 is a fragment of a typical IDR. LLPS plays pivotal roles in various biological processes, including transcription regulation, tumorigenesis, and chromatin organization (49). Transcription factors like MED1, YAP, and SP1 use LLPS to concentrate the transcriptional machinery at SEs (16, 18–20). Nevertheless, the role of LLPS in LSEC biology and liver diseases remains elusive. A novel mechanism was established in this study that PML NBs serve as scaffolds for the compartmentalization of BRD4 and SE-associated chromatins in a phase separation–dependent manner. Therefore, developing inhibitors that specifically target the interaction sites of the PML/BRD4 complex may offer a promising therapeutic strategy to ameliorate liver fibrosis.

This study has several limitations that warrant consideration. First, while we identified a BRD4/PML-mediated phenotypic shift in LSECs toward a proinflammatory state (LSEC_2 and LSEC_7), the precise molecular mechanisms governing this transition remain obscure and require further investigation. Second, the direct contribution of these phenotypically altered LSECs to HSC activation was not the focus of this study and remains to be further elucidated. Third, the translational potential of the BRD4/PML-TIMP1 axis inhibition in preventing liver fibrosis and its safety remain to be determined in human trials.

In summary, we elucidated the phase separation of PML and PML/BRD4-mediated SEs in the angiocrine signaling of LSECs, as well as the subsequent inflammatory cascade that recruits immune cells during liver fibrosis. Targeting these key molecular players involved in this LSEC phenotypic transition and angiocrine signaling might help improve liver inflammation and fibrosis.

## Methods

Further information can be found in Supplemental Methods.

### Sex as a biological variable.

Sex was not considered a biological variable in this study. Our study examined male and female humans and mice, and similar findings have been reported for both sexes.

### Human specimens.

Human liver samples were collected at West China Hospital, Sichuan University. Healthy liver samples (without fibrosis) were obtained from patients with hepatic hemangiomas who underwent endoscopic hepatectomy. The cirrhotic liver samples were collected from patients with histologically diagnosed liver cirrhosis. We used the METAVIR scoring system to determine the grade of liver fibrosis. The F0 stage was defined as healthy livers (*n* = 6), the F1 to F2 stages were defined as fibrotic livers (*n* = 12), and the F3 to F4 stages were defined as cirrhotic livers (*n* = 13). Patient information is listed in Supplemental Table 1.

### Mice.

C57BL/6J (WT), *Brd4*^fl/fl^, and *Pml*^fl/fl^ mice were obtained from GemPharmatech Co., Ltd, China. *Cdh5*^CreERT2^ mice were kindly provided by Bisen Ding at Sichuan University. *Brd4*^fl/fl^ and *Pml*^fl/fl^ mice were crossed with *Cdh5*^CreERT2^ mice to generate *Brd4*^fl/fl^/*Cdh5*^CreERT2^ and *Pml*^fl/fl^/*Cdh5*^CreERT2^ mice, respectively. At 6–7 weeks of age, these mice were intraperitoneally injected with tamoxifen (75 mg/kg/day) for 5 consecutive days to induce LSEC-specific *Brd4* (*Brd4*^ΔLSEC^) or *Pml* (*Pml*^ΔLSEC^) knockout. *dCas9-KRAB* mice were purchased from the Jackson Laboratory. *dCas9-KRAB* mice were crossed with *Cdh5*^CreERT2^ mice to generate *dCas9-KRAB*/*Cdh5*^CreERT2^ mice, after which tamoxifen was administered to induce dCas9-KRAB protein expression in LSECs. sgRNA targeting the SE of the *Pml* gene (AAV-sg*Pml*SE) was delivered to *dCas9-KRAB*/*Cdh5*^CreERT2^ mice via the tail vein to generate LSEC-specific *Pml* SE ablation mice. All these conditional knockout mice and littermate controls, as well as WT mice, were housed in the specific-pathogen-free environment at the West China Hospital Animal Experimental Center of Sichuan University. Both male and female mice were used in all these experiments. The protocols of the animal experiments were approved by the Laboratory Animal Ethics Committee of West China Hospital, Sichuan University.

### Statistics.

All data were analyzed with GraphPad Prism software (Version 10) or R statistical software (Version 4.4.1). The data are presented as mean ± standard error and were analyzed by unpaired 2-tailed Student’s *t*-test, one-way ANOVA with post hoc Tukey’s multiple comparisons or nested one-way ANOVA with post hoc Dunnett’s test. The correlation of BRD4^+^ LSECs with liver fibrotic scores was conducted by Pearson correlation coefficient analysis. *P* < 0.05 was regarded as statistically significant. For in vivo experiments, *n* indicates the number of individual experimental samples. For in vitro experiments, *n* indicates the data from independent biological replicates. Additional materials and methods are described in the Supplementary file.

### Study approval.

Human liver samples were collected with informed consent at West China Hospital, Sichuan University with institutional approval (No. HX2024-1923). All animal experiments were approved by the Animal Ethics Committees of West China Hospital, Sichuan University (IACUC No. 20220224070 and 20250211005). All research was conducted following both the Declaration of Helsinki and Istanbul.

### Data availability.

All RNA-seq, ChIP-seq, ATAC-seq, and scRNA-seq data generated in this publication will be available in the GEO database (GSE300053). All relevant data generated in this study are included in this article, supplementary information files, and source data files.

## Author contributions

JG contributed to the study design, funding support, manuscript revision, and overall study supervision. CG contributed to the study design, funding support, animal and cell experiments, data acquisition and analysis, and manuscript drafting. FW and ZH contributed to the study design, funding support, and manuscript revision. CT, VHS, and FT contributed to the study design, manuscript revision, and supervision. S Cao, ML, and HY contributed to the study design and manuscript revision. EL and HY contributed to bioinformatic data processing and analysis. EL, YT, ZY, BL, and BW contributed to the animal experiments and human liver sample collection. S Chen, CZ, WD, TL, ZC, YX, YG, and JC contributed to the genotyping, animal and cell experiments, and data analysis. The order of the shared first authors is determined by overall academic contributions and experimental workload.

## Conflict of interest

The authors have declared no conflict of interest exists.

## Funding support

- The National Natural Science Fund of China (82322011, U25A2020, and 82170623 to JG, 82170625 to CT, 82300711 to CG, 82470648 to ZH).
- The Shenzhen Medical Research Funds (B2302007 to FW).
- The National Key R&D Program of China (2023YFA1800801 to FW).
- The 135 projects for disciplines of excellence of West China Hospital, Sichuan University (ZYYC23026 to JG, ZYGD23029 to CT).
- The Postdoctor Research Fund of West China Hospital, Sichuan University (2024HXBH049 to CG).
- The International Science & Technology Cooperation Project of Sichuan (2025YFHZ0120 to JG).
- The Sichuan Science and Technology Program (2024NSFSC0641 to YT).

## Supplementary Material

Supplemental data

Unedited blot and gel images

Supplemental video 1

Supplemental video 2

Supplemental video 3

Supplemental video 4

Supplemental video 5

Supporting data values

## Version 1 03/17/2026

In-Press Preview

## Version 2 06/01/2026

Electronic publication
