# Supplementary material for: Epigenetically controlled endothelial promyelocytic leukemia drives liver inflammation and fibrosis
Source: J Clin Invest. 2026 Mar 17;136(11):e196730. doi: 10.1172/JCI196730 (PMC13221223; doi:10.1172/JCI196730)
Supplement: Supplemental data [file jci-136-196730-s110.pdf]

Materials and Methods in the Supplementary file

In vivo experiment

For carbon tetrachloride (CCl_4)-induced liver fibrosis, CCl_4 (1 μL/g body weight) was intraperitoneally injected into these mice twice a week for 6 weeks, and olive oil injection was used as a control. For 3,5-diethoxycarbonyl-1,4-dihydrocollidine (DDC)-induced liver fibrosis, the mice were fed a DDC or a control chow diet for 2 weeks to generate another liver fibrosis model. For iBET151 treatment, we intraperitoneally injected the BRD4 inhibitor iBET151 (6 mg/kg body weight) into the mice 4 times per week during the 6-week CCl_4 injection. For *Timp1* shRNA treatment, AAV-ENT conjugated with shRNA targeting *Timp1* was delivered to WT mice via the tail vein to generate LSEC-specific *Timp1* deletion mice (AAV-*Timp1*). The mice were humanely sacrificed 48 hours after the last administration.

Intravital imaging of mammalian livers

Advanced intravital microscopy (digital adaptive optical scanning light–field mutual iterative tomography, DAOSLIMIT) was utilized to observe the mouse liver microenvironments (1, 2). WT mice after 6 weeks of olive oil or CCl_4 administration and $Brd4^{fl/fl}$ and $Brd4^{\Delta LSEC}$ mice after 6 weeks of CCl_4 administration were used for DAOSLIMIT intravital microscopy. Antibodies and fluorescent dyes were injected 30 minutes before the experiments to label immune cells in mouse livers. Specifically, 5 μg of WGA, Ly6G, 3 μg of F4/80, 5 μg of CD63, CD166 and 60 μL of phosphate-buffered saline (PBS) were simultaneously injected into mice through the tail vein to label vessels, neutrophils, macrophages, and $CD63^+$ macrophages, respectively. Fifteen minutes after intravenous injection, the mice were anesthetized with Avertin (350 mg/kg, i.p.), followed by dissection to expose their livers, which were then mounted on a customized sample holder fitted with a 170-μm-thick coverslip for intravital imaging. To achieve an hour-long imaging duration, the laser power intensity was reduced to 1.0 mW/mm^2 at 488 nm, 1.8 mW/mm^2 at 561 nm, and 1.4 mW/mm^2 at 640 nm with a frame interval of 60 s, as described previously (3). The visualizations were performed after 3D reconstruction with background removal (3).

Portal venography

Direct portal venography was performed in a cirrhotic patient and a noncirrhotic patient with cavernous transformation of the portal vein (CTPV) via the jugular approach during the interventional procedure. In brief, right external jugular venous access was achieved by using Rupus-100 (Cook Incorporated, Bloomington, IN). After catheterization of the hepatic vein and hepatic venography, the liver parenchyma and the right portal vein were punctured via the intrahepatic route. Then, a 5-Fr catheter was advanced into the main portal vein, and portal venography images were acquired immediately after contrast agent injection.

Mouse liver cells isolation and culture

Primary mouse hepatocytes, LSECs, HSCs and macrophages were isolated from WT, *Brd4*$^{fl/fl}$, *Pml*$^{fl/fl}$, *Brd4*$^{\Delta LSEC}$, *Pml*$^{\Delta LSEC}$, AAV-*Timp1*, and AAV-*Pml*SE mice as previously described (4, 5). For hepatocytes, the mice were anesthetized with isoflurane before the livers were perfused with PBS buffer containing collagenase Type IA (Sigma, C9891). The perfused liver was dissociated and filtered through 70 μm cell strainers (Falcon, 352350), centrifuged (50 g, 5 minutes) to pellet hepatocytes. For non-parenchymal cells (NPCs), the mice were anesthetized with isoflurane before the livers were perfused with PBS buffer containing proteases (Roche, 25551121) and collagenase P (Roche, 11249002001). The perfused liver was dissociated and filtered through 70 μm and 40 μm cell strainers (Falcon, 352350 and 352340) to obtain a single-cell suspension. LSECs and macrophages were subsequently purified from this suspension using anti-CD146 and anti-F4/80 microbeads (Miltenyi Biotec, 130-092-007 and 130-110-443, respectively) in accordance with the manufacturer's protocols. HSCs were isolated from cell suspension by density gradient centrifugation as previously described (5). Isolated hepatocytes, HSCs and macrophages were then processed for qPCR analysis. The purified LSECs were cultured in endothelial cell medium (ScienCell Research Laboratories, 1001) and collected for further analysis.

BDR4 inhibitor treatment

Human liver sinusoidal endothelial cells (hLSECs, ScienCell Research Laboratories 5000) were cultured according to the manufacturer's protocols (4). To study PML expression, low-passage cells were starved with basal endothelial medium for 2 hours and then treated with a BRD4 inhibitor (iBET151, 2.5 μmol/L; Selleck, S2780) for 2 hours. Then, hLSECs were treated with human recombinant TNFα (50 ng/mL; PeproTech, 300-01A) for 6 hours before the cells were collected for further analysis.

Histone modification compound library

Starved hLSECs were pretreated with 152 inhibitors from a histone modification compound library (Table S2) for 2 hours. Then, hLSECs were treated with human recombinant TNFα for 6 hours before collection for qPCR analysis of the *CCL2* gene.

Plasmid transfection in HEK293T cells

FLAG-BRD4, HA-PML constructs (Hanbio Biotechnology) were transfected or co-transfected into HEK293T cells via Lipofectamine 2000 (Thermo Fisher). After 6 hours, the transfection mixture was replaced with fresh complete growth medium. Cells were harvested 48 hours post-transfection for subsequent Co-Immunoprecipitation (Co-IP) assay.

shRNA transduction of *PML* and *BRD4*

Lentivectors expressing sh*BRD4*, sh*PML,* sh*TIMP1*, or negative control shRNA (shCon) were transfected into HEK293T cells via Lipofectamine 2000. The sequences used for RNAi are shown in Table S3. Supernatants containing lentivirus were collected, centrifuged and filtered. hLSECs at 30% confluence were transduced with sh*BRD4*, sh*PML,* sh*TIMP1*, or shCon lentivirus via a transfection reagent (GeneChem) according to the manufacturer's protocols. The cells were then treated with puromycin (BRD4, TIMP1) or geneticin (PML) for 24 hours to select positive cells. The selected cells were passaged and treated with human TNFα for qPCR analysis of *BRD4, TIMP1* or *PML* gene expression.

Overexpression of *PML* and *BRD4*

The sequences of the *BRD4* and *PML* genes were inserted into the backbone vectors GV513 and GV492, respectively, according to the manufacturer's protocols. Lentivectors overexpressing the *BRD4* (*BRD4*^{OE}) or *PML* (*PML*^{OE}) gene were transfected into HEK293T cells, and supernatants containing lentivirus were collected. hLSECs at 30% confluence were transduced with *BRD4*^{OE} or *PML*^{OE} lentivirus via transfection reagent (GeneChem) according to the manufacturer's protocols. The cells were then treated with puromycin for 24 hours to select positive cells. The selected cells were passaged and treated with human TNFα for qPCR analysis of *BRD4* or *PML* gene expression.

CRISPR-dCas9 lentiviral transduction

SgRNAs were selected at different regions of the putative PML SE for the dCas9-KRAB targeting assay. Putative target regions were selected on the basis of the top peaks of BRD4 ChIP-seq, and sgRNA sequences were designed via the publicly available CHOPCHOP web tool. The synthesized sequences were inserted into the sgRNA backbone vector LentiGuide-Puro (Miaolingbio, Plasmid P0115) according to the manufacturer's protocols (6). The backbone vector was used as a control. The dCas9-KRAB lentivector (Miaolingbio, Plasmid P1595) or the dCas9-FLAG lentivector (Miaolingbio, Plasmid P34629) were synthesized by Miaoling Biology Co. Ltd. HEK293T cells were transfected with the dCas9 viral vector or sgRNA viral vector according to the manufacturer's instructions. The cells were cultured for 48 hours, and the supernatants containing the lentiviruses were collected. hLSECs were transduced with supernatant containing sgRNA lentivirus along with a 1:1000 dilution of polybrene (Millipore, TR1003-G). The cells were cultured for 48 hours before selection with puromycin. The selected cells were passaged and transduced with either dCas9-KRAB or dCas9-FLAG lentivirus concentrate, and hLSECs were treated with blasticidin (dCas9-KRAB) or puromycin (dCas9-FLAG) after 48 hours of transduction. The selected cells were passaged and treated with human TNFα for qPCR analysis of *PML* gene expression. The primers used are listed in Table S4.

SgRNA design and transfection

sgRNA sequences targeting the putative SE region of *Pml* were designed via the public CHOPCHOP web tool and synthesized by GenScript. For isolation of LSECs from *dCas9-KRAB*/*Cdh5*^{CreERT2} mice, synthetic sgRNA was transfected with Lipofectamine 2000 transfection reagent according to the manufacturer's protocols 18 hours before mouse recombinant TNFα was administered for 24 hours. The cells were then harvested for qPCR analysis. The sgRNA sequences are listed in Table S3.

AAV subcloning, production, and delivery

SgRNA2_2 was selected for subcloning into the AAV2 backbone because of its high efficiency in silencing *Pml* expression. The AAV2 backbone engineered with an endothelial-specific TIE1 promoter to drive the expression of both GFP and sgRNA (AAV-ENT-U6-sgRNA-TIE2-GFP) was obtained from Genechem (Shanghai, China). An AAV encoding a negative control (AAV-ENT-U6-sgRNA (non)-TIE2-GFP) was used as the nontargeting control (7). High-titer AAVs for systemic delivery were produced in AAV-ENT (high efficiency in targeting endothelial cells) by Genechem. *dCas9-KRAB*/*Cdh5*^{CreERT2} mice were injected with AAV-sg*Pml* (3×10^{11} viral genomes/mouse) or AAV-non (3×10^{11} viral genomes/mouse) via the tail vein via a 31-gauge needle. LSECs were isolated to detect *Pml* expression 2 weeks after AAV delivery. The AAV-sgRNA sequences are listed in Table S3.

For *Timp1*, an AAV2 backbone encoding sh*Timp1* or control shRNA was obtained from Genechem. High-titer AAVs for systemic delivery were produced in AAV-ENT by Genechem. WT mice aged 8 weeks were injected with AAV-*Timp1* (3×10^{11} viral genomes/mouse) or AAV-ENT (3×10^{11} viral genomes/mouse) via the tail vein via a 31-gauge needle. LSECs were isolated to detect *Timp1* expression 3 weeks after AAV delivery. The AAV-shRNA sequences are listed in Table S3.

Endogenously tagged cell line generation

The CRISPR/Cas9 approach was used to generate endogenously mEGFP-tagged PML.

Briefly, the sgRNA sequence (5' TCGGGCGGGTGCAGGCTCCA 3') that was targeted for PML was designed by CHOPCHOP and synthesized by Cyagen. Repair templates were cloned and inserted into an α-donor vector (Cyagen, Ltd.) containing mEGFP, a GS linker and homology arms flanking the insert, which was at the upstream ATG start codon of the PML gene. A total of 100,000 hLSECs were transfected with an α-donor vector and a ribonucleoprotein (RNP) complex containing the Cas9 protein and sgRNA according to the electroporation protocol. Colonies were picked via a limiting dilution assay. The primer pairs used for genotyping were as follows: Region 1, 5' CTCTCCCGCTTTACCGTAAGT 3' 3' TCCCTAATCTGAAGTCCTGCATC 5'; Region 2, 5' GTCCCATCATGCACAGCTGATCGT 3' 3' GCCACCAGACTTGTACAGCTCGT 5'; Region 3, 5' ACGAGCTGTACAAGTCTGGTGGC 3' 3' CTGTAATACGGTGTCCTCTGTAC 5'.

Fluorescence recovery after photobleaching (FRAP)

hLSECs stably expressing mEGFP-PML were cultured in a Col1-coated glass bottom dish for 24 hours. FRAP was performed on a Leica confocal microscope (Stellaris 8 Dive) with a 488 nm laser. The fluorescence intensity of the bleached cells at each time point was normalized to the fluorescence intensity in the background region and the fluorescence intensity of adjacent unbleached cells. The images were analyzed via Leica software.

Protein purification

cDNA encoding the PML-IDR was cloned and inserted into a modified version of a pET-28a expression vector (Genecreate). The base vector was engineered to include a 5'6×HIS followed by either mEGFP or a 14 amino acid linker sequence "GAPGSAGSAAGGSG." NEBuilder® HiFi DNA Assembly Master Mix (NEB E2621S) was used to insert the PML-IDR sequence (generated by PCR) in-frame with the linker sequence. The vector expressing mEGFP alone contains the linker sequence followed by a stop codon.

In vitro droplet assay

The recombinant mEGFP fusion protein was concentrated via centrifugal filters (30K MWCO, Millipore). Recombinant protein was added to solutions at the indicated concentrations, and 15% PEG-8000 (Sigma) was added as a crowding agent in droplet formation buffer (50 mM Tris-HCl (pH 7.5), 10% glycerol, and 1 mM DTT). The protein mixture was immediately loaded onto a homemade chamber comprising a glass slide with a coverslip attached to two parallel strips of double-sided tape. The slides were then imaged with a Leica confocal microscope with a 63× objective (Stellaris 8 Dive).

Cell coculture and transwell assay

hLSECs were transduced with either sh*TIMP1* or shCon lentivirus for 24 hours prior to TNFα treatment. Following this incubation, the conditioned media were collected, or hLSECs were cocultured with THP1 cells. For IF staining, THP1 cells were plated on glass-bottom chamber slides and pretreated with phorbol myristate acetate (PMA) before exposure to conditioned media. Then the conditional media from THP1 cells were collected to treat LX2 cells for subsequent IF staining. For the transwell assay, THP1 cells were plated on the upper chamber of a 24-well transwell insert (NEST, 725301, 8 μm pore size), followed by PMA treatment. Subsequently, hLSECs transfected with either sh*TIMP1* lentivirus or shCon lentivirus and treated with TNFα were cocultured in the lower chamber. Conditional media from hLSECs treated with TNFα and TNFα plus BRD4 inhibitor iBET151 were treated to LX2 cell for subsequent western blot analysis. In a parallel mouse primary cell experiment, isolated primary HSCs from WT mice were cocultured with primary LSECs isolated from AAV-*Timp1*, *Brd4*$^{\Delta LSEC}$ mice and their littermate controls with olive oil and CCl_4 treatment followed by IF staining of HSCs.

Hematoxylin and eosin (H&E) and Sirius red staining

Liver tissues were fixed in 4% formaldehyde, embedded in paraffin, and cut into 5 μm sections. The sections were deparaffinized and rehydrated for H&E and Sirius red

staining. For H&E staining, the slides were stained with hematoxylin for 3 minutes, after which they were rinsed with running tap water. The slides were subsequently stained with eosin, rinsed, dehydrated, and mounted with a resinous medium. The tissue sections were stained with Sirius red to assess collagen deposition. After deparaffinization and rehydration, the slides were stained with Sirius red before they were rinsed, dehydrated, and mounted with resinous medium. Images of H&E- and Sirius red-stained samples were captured via an OLYMPUS microscope (CX41) and analyzed via ImageJ software.

Immunofluorescence (IF) staining

Paraffin sections were deparaffinized and rehydrated as previously described. Heat-induced epitope retrieval (HIER) was performed in citrate or Tris-EDTA buffer before blocking in 10% serum for 1 hour at room temperature. Then, the slides were incubated with primary antibodies overnight at 4 °C. After incubation with fluorochrome-coupled secondary antibodies and DAPI, images were captured via an OLYMPUS microscope (CX41) or Leica confocal microscope (Stellaris 5), and the data were analyzed via ImageJ. For CD31 staining, tissue sections (8–10 μm thick) were imaged via Z-stack acquisition, followed by 3D reconstruction. Imaging was performed via a Leica confocal microscope (Stellaris 5). The primary antibodies used for IF staining are listed in Table S5.

Scanning electron microscopy (SEM)

Mice were anesthetized and perfused via the portal vein with freshly prepared 3% glutaraldehyde fixative. Following perfusion, liver lobes were dissected and immersed in the same 3% glutaraldehyde fixative overnight at 4°C. After fixation, liver tissues were washed in PBS, rinsed in water, and dehydrated through a graded series of ethanol. The dehydrated samples were then subjected to critical point drying using carbon dioxide, mounted on an aluminum stub, and sputter-coated with gold-palladium for 90 seconds. Images were acquired using a JSM-IT700HR InTouchSoope^{TM} scanning electron microscope.

Flow cytometry (FC) analysis

Primary mouse NPCs were isolated from the livers of *Brd4*$^{\Delta LSEC}$*, Pml*$^{\Delta LSEC}$, AAV-*Timp1* mice and their respective littermate controls with olive oil and CCl_4 treatment, as previously described. The NPC suspension was centrifuged at 300g for 10 minutes at 4°C to obtain a cell pellet, which was then resuspended in PBS buffer. Cells were first incubated with Fc receptor blocking solution (anti-CD16/32; BD, 553142) in the dark for 5 minutes at 4°C to minimize nonspecific antibody binding. Antibody staining was performed by incubating the samples with the fluorochrome-conjugated antibodies for 30 minutes at 37°C in the dark. The following anti-mouse antibodies were used: anti-CD45, anti-CD11b, anti-Ly6c, anti-F4/80 and anti-CD63. Prior to acquisition, cells were stained with DAPI to discriminate live (DAPI-negative) or dead (DAPI-positive) populations. All samples were acquired on a BD FACS Symphony flow cytometer, and data were analyzed using FlowJo software (Version 10). CD63^{+} MoMFs were defined as CD45^{+}CD11b^{+}Ly6c^{hi}F4/80$^{int/lo}$CD63^{+}.

RNA-fluorescence in situ hybridization (FISH) combined with IF

For RNA-FISH, a probe complementary to the *Timp1* target RNA sequence covering the region 5—785, *Ccl2* target RNA sequence covering the region 21—785, *TIMP1* target RNA sequence covering the region 2—765, *CCL2* target RNA sequence covering the region 1—720 was designed by patented algorithms (CHINA patent number ZL202110581853.9). The paraffin-embedded slides were deparaffinized and rehydrated as previously described. The slides were incubated with Pre-A buffer to inhibit endogenous peroxidase. Next, the slides were digested with Proteinase II before heat-induced epitope retrieval in Pre-B buffer. The FISH procedure was conducted according to the instructions of a PinpoRNA 2.0 kit (#PIF1000, Pinpoease, China). After that, the slides were incubated with 10% serum, primary antibodies, fluorochrome-coupled secondary antibodies, and DAPI according to the IF staining protocol. Images were captured via a Leica confocal microscope with a 63× objective (Stellaris 5).

DNA-FISH combined with IF

For DNA-FISH, probes complementary to *TIMP1* target the DNA sense strand covering the region 2070-3985 and *CCL2* targets the DNA antisense strand covering the region 270-2810; these probes were designed via patented algorithms (CHINA patent number ZL202110581853.9). hLSECs for DNA-FISH were cultured on glass coverslips, fixed with 4% paraformaldehyde in PBS for 10 minutes and washed with PBS. The slides were subsequently incubated with RNase and Pre-A buffer. Next, the slides were digested with Proteinase II for 15 minutes at 40 °C before heat-induced epitope retrieval in pretreatment buffer B for 15 minutes. The FISH procedure was conducted according to the instructions of a PinpoDNA 2.0 kit (#PDF1000, Pinpoease, China). Images were captured via a Leica confocal microscope with a 63× objective (Stellaris 5).

RNA-Seq, ChIP-Seq, ATAC-seq, and Hi-C

hLSECs were starved of basal endothelial medium for 2 hours, followed by treatment with a BRD4 inhibitor (iBET151, 2.5 μmol/L) for 2 hours. Then, hLSECs were treated with human recombinant TNFα (50 ng/mL) for 6 hours before the cells were collected for RNA-Seq, ChIP-Seq, ATAC-seq, and Hi-C. All the raw data have been deposited in Gene Expression Omnibus (GEO) database with accession of GSE300053.

RNA-Seq process and data analysis

The cells used for RNA-seq were collected and washed twice with cold PBS, after which total RNA was extracted. Then, the mRNA was purified from total RNA via poly-T oligo-attached magnetic beads. Strand cDNA was synthesized via random hexamer primers, followed by second-strand cDNA synthesis. The libraries then underwent PCR amplification and purification. After the libraries passed quality control, they were pooled and sequenced on the Illumina sequencing platform.

The RNA-seq reads were aligned to the hg38 revision of the human genome via STAR (v2.7.3a) (8). Mapped reads were counted via HTseq-count (v0.11.3) (9). Gene expression was quantified as reads per kilobase per million mapped reads (RPKM), and

protein-encoding genes with an RPKM ≥1 in at least one sample were selected for differential analysis. Differentially expressed genes (DEGs) between groups were identified via the edgeR package (v3.28.1) (10) in R 3.6.0, with cutoffs of $P<0.05$ and absolute log2 (fold change) ≥1. RNA expression at specific locations was visualized via Integrative Genomics Viewer (IGV) software.

ChIP-Seq, ATAC-seq process and data analysis

The cells used for ChIP-seq were harvested and crosslinked with 1% formaldehyde in PBS, followed by quenching with 125 mM glycine on ice. The samples were washed with cold PBS and collected. The cells were then pelleted for 10 min at 4 °C, resuspended in lysis buffer I (50 mM HEPES-KOH, pH 7.5; 140 mM NaCl; 1 mM EDTA; 10% glycerol; 0.5% NP-40; 0.25% Triton X-100; and protease inhibitors), rotated for 10 min at 4 °C, and then resuspended in lysis buffer II (10 mM Tris-HCl, pH 8.0; 200 mM NaCl; 1 mM EDTA; 0.5 mM EGTA; and protease inhibitors). The cells were rotated once more for 10 min at 4 °C and resuspended in sonication buffer (20 mM Tris-HCl pH 8.0, 150 mM NaCl, 2 mM EDTA pH 8.0, 0.1% SDS, and 1% Triton X-100, protease inhibitors) for sonication. Sonicated lysates were centrifuged for 10 minutes at 4 °C. The sonicated chromatin was then incubated with magnetic beads bound with H3K27ac, BRD4 or PML antibodies overnight at 4 °C to enrich for DNA. The beads were then sequentially washed with wash buffer (50 mM HEPES-KOH pH 7.5, 500 mM LiCl, 1 mM EDTA pH 8.0, 0.7% Na-deoxycholate, and 1% NP-40) and TE buffer (10 mM Tris-HCl pH 8.0, 1 mM EDTA, and 50 mM NaCl). Chromatin elution was performed by incubation in elution buffer (50 mM Tris-HCl pH 8.0, 10 mM EDTA, 1% SDS) at 65 °C for 30 minutes. The cross-links were reversed overnight at 65 °C. To remove protein, proteinase K was added, and the mixture was incubated at 37 °C for 2 hours. The DNA was then purified, amplified via PCR, and sequenced on the Illumina platform.

The ATAC-seq libraries were generated following a previously published protocol (11). The cells were washed with cold PBS and then centrifuged for 5 minutes. The

cells were subsequently lysed with cold lysis buffer (10 mM Tris-HCl, pH 7.4; 10 mM NaCl; 3 mM $MgCl_2$; and 0.1% IGEPAL CA-630), followed by centrifugation for 10 min at 4 °C. The pellets were subsequently resuspended in transposase reaction mixture (25 µL of 2× TD buffer, 2.5 µL of transposase (Illumina), and 22.5 µL of nuclease-free water), and transposition was carried out for 30 minutes at 37 °C. The sample was then purified, and the DNA was amplified via PCR. The libraries were then sequenced on the Illumina platform.

The ChIP-seq data and ATAC-seq data were processed similarly. The sequencing data were first quality assessed via the ChIPQC package (v1.21.0) (12). The reads were then mapped to the human reference genome hg38 via Bowtie2 (v2.3.1) (13). Duplicated mapped reads were discarded via the MarkDuplicates function from Picard (v2.25.6, https://github.com/broadinstitute/picard). Narrow peaks from H3K27ac, BRD4, and PML were identified via MACS2 (14). The differentially bound sites were identified via Diffbind (v3.4.11) (15) at cutoffs of $P<0.05$ and absolute log2 (fold change) ≥1. Using Deeptools (v3.5.6) (16), the ChIP and ATAC signal profiles in the TSS±5 kb region were normalized, and the normalized values for the differentially expressed genes were used to generate profile plots. The ATAC signal profile within ±5 kb of the differentially bound sites was used to generate heatmaps. Super enhancer calling was performed via BRD4 ChIP-seq data via the ROSE tool (v0.1) (17, 18), which uses peaks called with MACS2. Genes that overlap with the superenhancers and genes in the vicinity of the superenhancers were determined to be under the influence of the superenhancer. The ChIP signal tracks were visualized via IGV.

Hi-C process and data analysis

Hi-C library preparation was carried out as previously described (19, 20) with modifications. Briefly, approximately 100,000 to 1,000,000 cell pellets were used as inputs per sample. Nuclei were digested overnight with MboI at 37 °C, followed by end-labeling of the DNA with biotin using 25 µL of fill-in master mix at 37 °C for 2 hours. The DNA was then ligated via the addition of 450 µL of ligation master mix at

room temperature for 4 hours. DNA was purified via phenol–chloroform extraction. To remove linear DNA, the sample was treated with Lambda exonuclease (NEB) and Exonuclease I (NEB) at 37 °C for 30 minutes. Fragmentation of 100–500 ng of DNA was performed via the TruePrep DNA Library Prep Kit V2 (Vazyme). Biotin-labeled DNA was enriched via pull-down with Dynabeads MyOne Streptavidin T1 beads (Life Technologies). Library amplification and size selection were conducted via TruePrep Amplify Enzyme (Vazyme) and SPRIselect Beads (Beckman Coulter). Libraries were sequenced on an Illumina platform.

Hi-C reads were processed via the Hi-C Pro pipeline (v3.1.0) (21). The reads were first aligned to the hg38 reference genome via Bowtie2, the aligned reads were assigned to MboI restriction fragments, and the interaction pairs were reconstructed. A quality assessment was subsequently performed, and singleton and multihit pairs and failed ligation products were filtered. The remaining valid pairs were then used to build contact matrices and normalized via iterative correction and eigenvector decomposition (22). The TADs within each sample were then identified via TopDom (v0.0.2) (23) on 40 kb-resolution ICE-normalized contact matrices. TAD reorganization of the TADs was then identified following a previously published protocol (24). Specifically, the domain directions function from diffHiC (v1.18.0) (25) was used to calculate the direction index. We subsequently performed a significant difference test through the glmQLFTest function of edgeR, where raw TopDom TAD boundaries with FDR<0.05 were considered reorganizations. Juicebox (v1.11.08) (26) was used to visualize the Hi-C contact matrices.

Single-cell RNA sequencing (scRNA-seq) data analysis

Liver tissues from *Brd4*$^{fl/fl}$, *Pml*$^{fl/fl}$, *Brd4*$^{\Delta LSEC}$ and *Pml*$^{\Delta LSEC}$ mice were perfused according to the protocol of LSEC isolation to obtain single-cell suspensions. The cells were subsequently centrifuged at 50 × g for 5 minutes to remove hepatocytes. The collected suspension was centrifuged at 300 × g for 10 minutes to obtain a cell pellet, which was washed twice and resuspended in cold PBS with 0.04% BSA. The cells were subsequently counted via a hemocytometer. After the cells were counted, 10,000 cells

were chosen as the appropriate target volume for each sample. The samples were then loaded onto a 10x Genomics single-cell A chip. After the single-cell suspensions were prepared, the following steps were executed following the Chromium Single-cell 3' Reagent v3 user guide. In brief, droplets were generated with single cells and barcoded beads, after which cDNA synthesis was performed. The droplets were broken, the cDNA was amplified, and libraries were constructed. Finally, the single-cell libraries were sequenced on the Illumina platform.

scRNA-seq reads were first processed with 10x Genomics Cell Ranger (v7.2.0) (27). Cells with fewer than 200 unique genes, fewer than 500 transcripts or more than 10% mitochondrial reads were filtered out. The Seurat package (v5.1.0) (28) was used for further processing, cell clustering, and downstream analysis. After normalization, we identified the top variable features in each sample. The cells were subsequently divided into clusters via the shared nearest neighbor (snn) method. We applied tSNE to visualize the cell clusters. The classification and annotation of the cells were based on known marker genes from the literature review and the CellMarker 2.0 database (29). Endothelial cells and macrophages from the samples were then isolated and analyzed via similar procedures. Single-cell level gene set variance analysis was carried out via the GSVA package (v2.0.4) (30). Differential expression analysis between cell clusters was performed via the Wilcoxon rank sum test with the FindMarkers function from Seurat, and the threshold was set as an absolute log2 (fold change) ≥1 and $P<0.05$. Pathway enrichment analysis of the DEGs was carried out via the R package clusterProfiler (v4.12.6) (31). We used the R package nichenetr (v2.1.5) (32) to analyze ligand–receptor interactions between the EC clusters and macrophages. The R package monocle3 (v1.42.0) (33) was used to perform UMAP reduction, trajectory, and pseudotime analysis on the endothelial cell and macrophage data.

Quality control of sequencing reads

All Illumina sequencing reads used in the study were quality controlled by Trim Galore (v0.6,5, https://github.com/FelixKrueger/TrimGalore). Reads with a base quality lower

than 20 were discarded, and adaptor sequences were removed from the 3' end. Reads shorter than 50 nt were filtered out.

Real-time quantitative PCR (RT–qPCR)

Total RNA was extracted from cultured cells or liver tissues via an RNeasy Mini Kit (Foregene, RE-03014) following the manufacturer's instructions. The OD 260/280 ratio and concentration of the purified RNA solution were measured via a NanoDrop spectrophotometer. Reverse transcription was performed via a First-Strand cDNA Synthesis Kit (Thermo Fisher, K1622). The obtained cDNA was further used for qPCR with SYBR Green Supermix (Bimake, B21202) according to the manufacturer's instructions. The mRNA levels in the murine samples were normalized to those of the β-actin gene, and the mRNA levels in the human samples were normalized to those of the GAPDH gene. The sequences of primers used are listed in Table S4.

Genotyping

Genotyping was performed according to the manufacturer's protocol (Mouse Genotyping Kit, Ubigene Biosciences, YK-MG-1000). Mouse tails were lysed in tail lysis buffer at 95 °C for 15 minutes. After lysis, the supernatant was collected to obtain DNA. The PCR system was subsequently prepared according to the instructions provided in this kit, and the results were analyzed via agarose gel electrophoresis. All the mice were genotyped via specific primers designed to differentiate the knockout allele from the wild-type allele. The sequences of primers used in this study are provided in Table S4.

Molecular docking and intrinsically disordered region (IDR) prediction

The molecular docking of the PML-BRD4 and TIMP1-CD63proteins was performed on the GRAMM-X platform and visualized via the PDBePISA platform. The 3D structures of proteins were retrieved from the Protein Data Bank (PDB) and prepared for docking. The docking procedure was performed via predefined settings on the GRAMM-X platform, and the results were analyzed and visualized via the PDBePISA

platform. For IDR prediction, a publicly available database (https://iupred3.elte.hu/plot) was utilized. Additionally, the protein binding region was predicted via the HybridPBRpred server (http://biomine.cs.vcu.edu/servers/hybridPBRpred/).

Co-IP assay

IP was performed according to the manufacturer's protocol (Thermo Fisher, 88804). hLSECs or HEK293T cells were lysed in IP buffer supplemented with a protease inhibitor cocktail. The cell lysates were incubated with BRD4 or PML IP antibodies (for hLSECs), or with FLAG or HA IP antibodies (for HEK293T cells) at 4 °C overnight, and then the protein-A/G magnetic beads were incubated with the cell lysates at room temperature for 2 hours. Normal rabbit IgG (Millipore Sigma, 12-370) was utilized as a control. The immunoprecipitated proteins were loaded onto SDS–PAGE gels and analyzed by immunoblotting as previously described. Antibody information is shown in Table S5.

Glutathione-S-transferase (GST) fusion protein construction and pull-down assay

GST fusion constructs encoding BRD4 fragments (amino acids 44-168, 333-460, 524-683, and 1110-1362) and PML fragments (amino acids 49-104, 120-229, 230-360, and 361-574) were synthesized and cloned by Genscript Corporation (Nanjing, China). All the constructs were sequence-verified, and detailed cloning strategies, protein expression and purification are provided in the Certificate of Analysis (COA) for each fragment. The GST pull-down assay was conducted following the manufacturer's protocol (GENECREATE, JKR23011). Briefly, GST-tagged proteins were expressed and purified from bacteria and then incubated with glutathione beads at room temperature for 2 hours. The beads containing the GST-tagged proteins were subsequently incubated with total protein extracts from hLSECs at 4 °C overnight. The bound proteins were then eluted before they were analyzed by immunoblotting to detect protein interactions.

Protein extraction and Western blot analysis

Proteins from liver tissues and cells were extracted via radioimmunoprecipitation lysis buffer (RIPA buffer; Keygen Biotech, KGB5303-100). Then, the liver tissue homogenate or cell lysate was centrifuged at 12,000 rpm before the protein concentration was determined. A total of 30–50 μg of protein was loaded onto an SDS–PAGE gel and transferred onto a PVDF membrane. The membrane was blocked with 5% nonfat milk for 1 hour before being incubated with a primary antibody overnight at 4 °C. After being washed with TBST, the membrane was incubated with secondary antibodies for 1 hour. The signals of the bands were developed and detected via an enhanced chemiluminescence detection kit (Biotite, P0018AM), and the results were quantified via ImageJ software. HSC70 for mice and GAPDH for humans were used as loading controls. The primary and secondary antibodies used in this study are listed in Table S5.

Publicly available database

Human (GSE109774, GSE136103) and mouse (GSE218299) liver scRNA-seq data were obtained from the Gene Expression Omnibus (GEO) database. The data were processed and analyzed via the R package Seurat via strategies similar to those described in the previous sections regarding scRNA-seq data analysis. HUVEC ChIP-seq (GSE53998) and mouse isolated LSEC ChIP-seq data (GSE154828) were obtained from the GEO database. The data were analyzed via the Integrative Genomics Viewer (IGV).

Reference

1. Wu J, Lu Z, Jiang D, Guo Y, Qiao H, Zhang Y, et al. Iterative tomography with digital adaptive optics permits hour-long intravital observation of 3D subcellular dynamics at millisecond scale. *Cell.* 2021;184(12).
2. Lu Z, Cai Y, Nie Y, Yang Y, Wu J, and Dai Q. A practical guide to scanning light-field microscopy with digital adaptive optics. *Nat Protoc.* 2022;17(9):1953–79.
3. Lu Z, Liu Y, Jin M, Luo X, Yue H, Wang Z, et al. Virtual-scanning light-field microscopy for robust snapshot high-resolution volumetric imaging. *Nat Methods.* 2023;20(5):735–46.
4. Gan C, Yaqoob U, Lu J, Xie M, Anwar A, Jalan-Sakrikar N, et al. Liver sinusoidal endothelial cells contribute to portal hypertension through collagen type IV-driven sinusoidal remodeling. *JCI Insight.* 2024;9(11).
5. Mederacke I, Dapito DH, Affò S, Uchinami H, and Schwabe RF. High-yield and high-purity isolation of hepatic stellate cells from normal and fibrotic mouse livers. *Nat Protoc.* 2015;10(2):305–15.
6. Liu M, Cao S, He L, Gao J, Arab JP, Cui H, et al. Super enhancer regulation of cytokine-induced chemokine production in alcoholic hepatitis. *Nat Commun.* 2021;12(1):4560.
7. Zhao WJ, Qian Y, Zhang YF, Yang AH, Cao JX, Qian HY, et al. Endothelial FOSL1 drives angiotensin II-induced myocardial injury via AT1R-upregulated MYH9. *Acta Pharmacol Sin.* 2025;46(4):922–39.
8. Dobin A, Davis CA, Schlesinger F, Drenkow J, Zaleski C, Jha S, et al. STAR: ultrafast universal RNA-seq aligner. *Bioinformatics.* 2013;29(1):15–21.
9. Anders S, Pyl PT, and Huber W. HTSeq—a Python framework to work with high-throughput sequencing data. *Bioinformatics (Oxford, England).* 2014;31(2):166–9.
10. McCarthy DJ, Chen Y, and Smyth GK. Differential expression analysis of multifactor RNA-Seq experiments with respect to biological variation. *Nucleic Acids Research.* 2012;40(10):4288–97.
11. Buenrostro JD, Giresi PG, Zaba LC, Chang HY, and Greenleaf WJ. Transposition of native chromatin for fast and sensitive epigenomic profiling of open chromatin, DNA-binding proteins and nucleosome position. *Nature Methods.* 2013;10(12):1213–8.
12. Carroll TS, Liang Z, Salama R, Stark R, and de Santiago I. Impact of artifact removal on ChIP quality metrics in ChIP-seq and ChIP-exo data. *Front Genet.* 2014;5:75.
13. Langmead B, and Salzberg SL. Fast gapped-read alignment with Bowtie 2. *Nature Methods.* 2012;9(4):357–9.
14. Zhang Y, Liu T, Meyer CA, Eeckhoute J, Johnson DS, Bernstein BE, et al. Model-based Analysis of ChIP-Seq (MACS). *Genome Biology.* 2008;9(9):R137.
15. Ross-Innes CS, Stark R, Teschendorff AE, Holmes KA, Ali HR, Dunning MJ, et al. Differential oestrogen receptor binding is associated with clinical outcome in breast cancer. *Nature.* 2012;481(7381):389–93.

16. Ramírez F, Ryan DP, Grüning B, Bhardwaj V, Kilpert F, Richter AS, et al. deepTools2: a next generation web server for deep-sequencing data analysis. *Nucleic Acids Res.* 2016;44(W1):W160–5.
17. Whyte WA, Orlando DA, Hnisz D, Abraham BJ, Lin CY, Kagey MH, et al. Master transcription factors and mediator establish super-enhancers at key cell identity genes. *Cell.* 2013;153(2):307–19.
18. Lovén J, Hoke HA, Lin CY, Lau A, Orlando DA, Vakoc CR, et al. Selective inhibition of tumor oncogenes by disruption of super-enhancers. *Cell.* 2013;153(2):320–34.
19. Rao Suhas SP, Huntley Miriam H, Durand Neva C, Stamenova Elena K, Bochkov Ivan D, Robinson James T, et al. A 3D Map of the Human Genome at Kilobase Resolution Reveals Principles of Chromatin Looping. *Cell.* 2014;159(7):1665–80.
20. Zhang C, Xu Z, Yang S, Sun G, Jia L, Zheng Z, et al. tagHi-C Reveals 3D Chromatin Architecture Dynamics during Mouse Hematopoiesis. *Cell Reports.* 2020;32(13):108206.
21. Servant N, Varoquaux N, Lajoie BR, Viara E, Chen C-J, Vert J-P, et al. HiC-Pro: an optimized and flexible pipeline for Hi-C data processing. *Genome Biology.* 2015;16(1):259.
22. Ramachandran P, Dobie R, Wilson-Kanamori JR, Dora EF, Henderson BEP, Luu NT, et al. Resolving the fibrotic niche of human liver cirrhosis at single-cell level. *Nature.* 2019;575(7783):512–8.
23. Shin H, Shi Y, Dai C, Tjong H, Gong K, Alber F, et al. TopDom: an efficient and deterministic method for identifying topological domains in genomes. *Nucleic Acids Research.* 2015;44(7):e70–e.
24. Wang J, Yu H, Ma Q, Zeng P, Wu D, Hou Y, et al. Phase separation of OCT4 controls TAD reorganization to promote cell fate transitions. *Cell Stem Cell.* 2021;28(10):1868–83.e11.
25. Lun ATL, and Smyth GK. diffHic: a Bioconductor package to detect differential genomic interactions in Hi-C data. *BMC Bioinformatics.* 2015;16(1):258.
26. Durand NC, Robinson JT, Shamim MS, Machol I, Mesirov JP, Lander ES, et al. Juicebox Provides a Visualization System for Hi-C Contact Maps with Unlimited Zoom. *Cell Syst.* 2016;3(1):99–101.
27. Zheng GXY, Terry JM, Belgrader P, Ryvkin P, Bent ZW, Wilson R, et al. Massively parallel digital transcriptional profiling of single cells. *Nature Communications.* 2017;8(1):14049.
28. Hao Y, Stuart T, Kowalski MH, Choudhary S, Hoffman P, Hartman A, et al. Dictionary learning for integrative, multimodal and scalable single-cell analysis. *Nature Biotechnology.* 2024;42(2):293–304.
29. Hu C, Li T, Xu Y, Zhang X, Li F, Bai J, et al. CellMarker 2.0: an updated database of manually curated cell markers in human/mouse and web tools based on scRNA-seq data. *Nucleic Acids Research.* 2023;51(D1):D870–D6.
30. Hänzelmann S, Castelo R, and Guinney J. GSVA: gene set variation analysis for microarray and RNA-Seq data. *BMC Bioinformatics.* 2013;14(1):7.

31. Wu T, Hu E, Xu S, Chen M, Guo P, Dai Z, et al. clusterProfiler 4.0: A universal enrichment tool for interpreting omics data. *The Innovation.* 2021;2(3).
32. Browaeys R, Saelens W, and Saeys Y. NicheNet: modeling intercellular communication by linking ligands to target genes. *Nature Methods.* 2020;17(2):159–62.
33. Qiu X, Mao Q, Tang Y, Wang L, Chawla R, Pliner HA, et al. Reversed graph embedding resolves complex single-cell trajectories. *Nature Methods.* 2017;14(10):979–82.

Supplementary figures

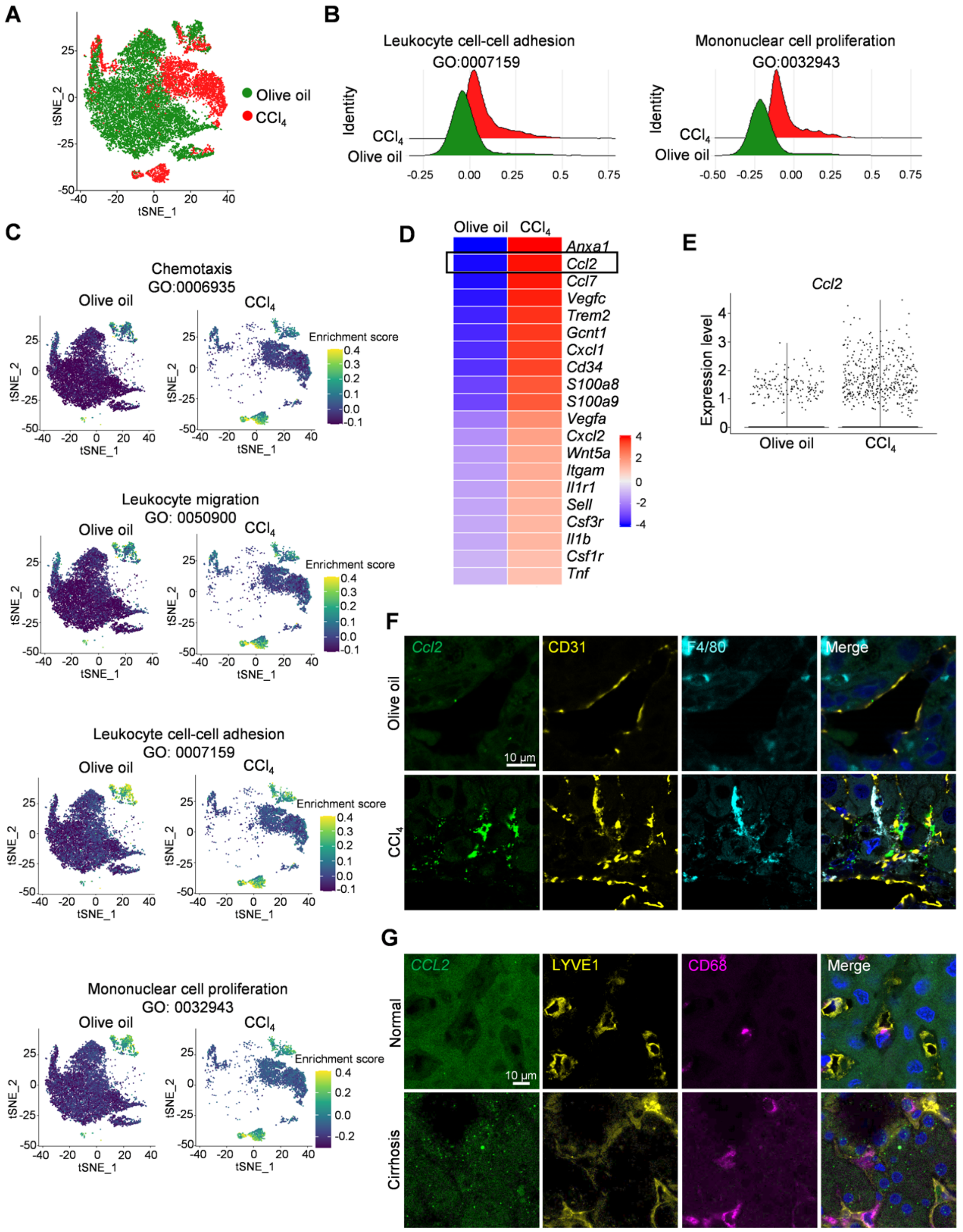

Figure S1. Liver endothelial cells are responsible for inflammatory immune cell recruitment during the progression of liver fibrosis. (A-E) scRNA-seq analysis was performed on mouse livers subjected to olive oil and CCl_4 for 6 weeks. t-SNE plot of liver sinusoidal endothelial cells (LSECs, A). Ridge plot showing enriched pathways in LSECs between olive oil- and CCl_4-treated mice (B). tSNE plot showing enriched

signaling pathways in LSECs between olive oil- and CCl_4-treated mice (C). Heatmap of the top upregulated genes in LSECs induced by CCl_4 treatment (D). *Ccl2* expression in total LSECs from mice treated with olive oil or CCl_4 (E). **(F)** RNA-FISH analysis of the *Ccl2* probe and proteins (CD31 and F4/80) in liver sections from olive oil- or CCl_4-treated mice. **(G)** RNA-FISH analysis of *CCL2* probes and proteins (LYVE1 and CD68) in liver sections from healthy controls or cirrhotic patients.

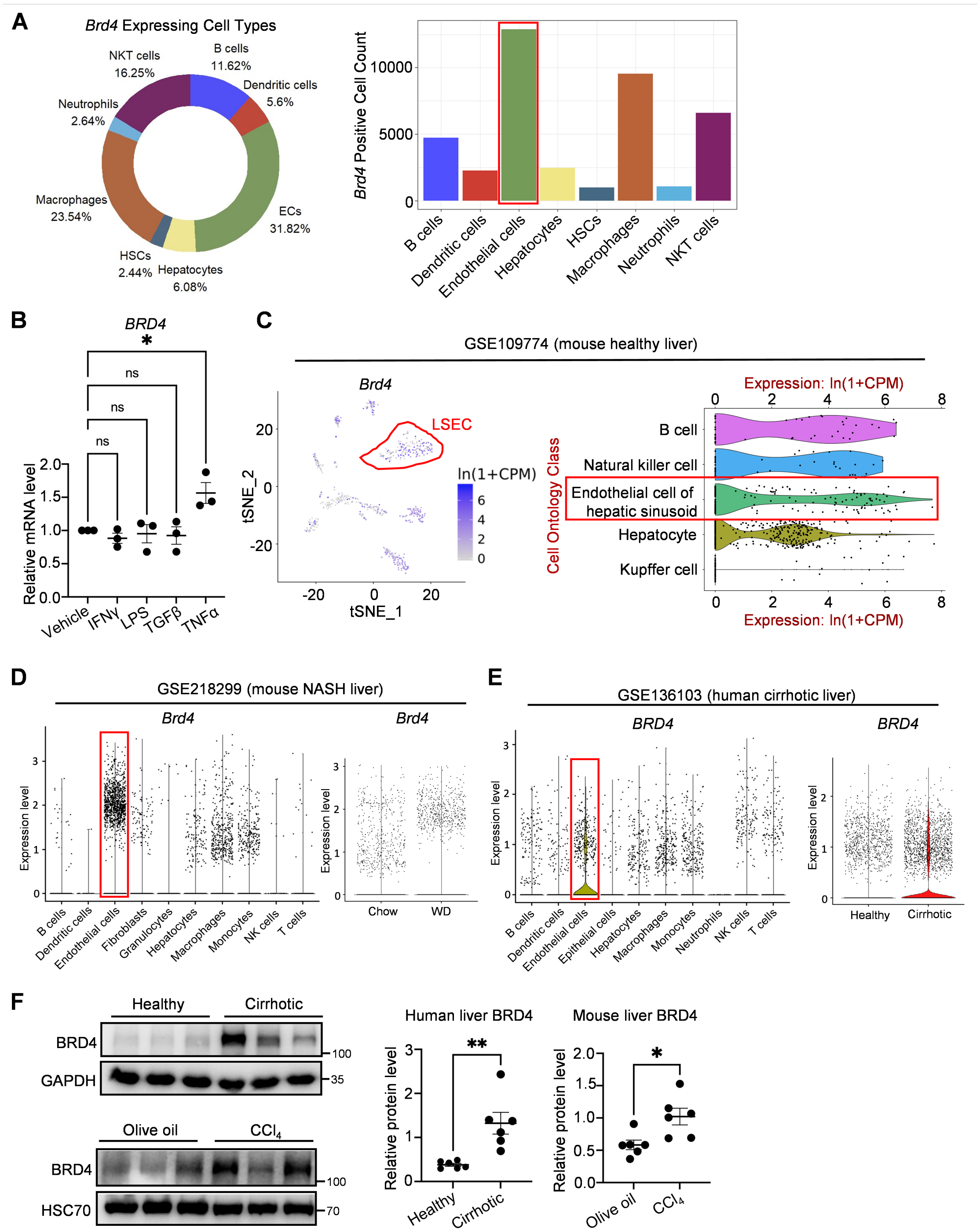

Figure S2. Increased BRD4 expression in diseased human and mouse livers. (A) The percentage of *Brd4*-expressing cell types (left) and *Brd4*-positive cell counts among liver cell types in CCl_4-treated mouse livers (right) from our scRNA-seq analysis. **(B)** qPCR analysis of the *BRD4* gene in hLSECs treated with a panel of inflammatory stimuli (IFNγ, LPS, TGFβ, and TNFα) (n=3, representing 3 independent experiments). **(C-E)** Published scRNA-seq analysis revealed that LSECs are the main cell type

expressing BRD4 in healthy mouse livers (GSE109774, C), NASH mouse livers (GSE218299, D), and human cirrhotic livers (GSE136103, E). LSEC-derived BRD4 was increased in mouse NASH livers (D) and human cirrhotic livers (E). **(F)** Western blot analysis and quantification of BRD4 in human and mouse cirrhotic livers (n=6/group). Western diet, WD. ***P*<0.01, **P*<0.05 and ns, not significant. The data are presented as the means ± SEMs; one-way ANOVA with Tukey's multiple comparison test for (B) and two-tailed Student's *t* test for (F).

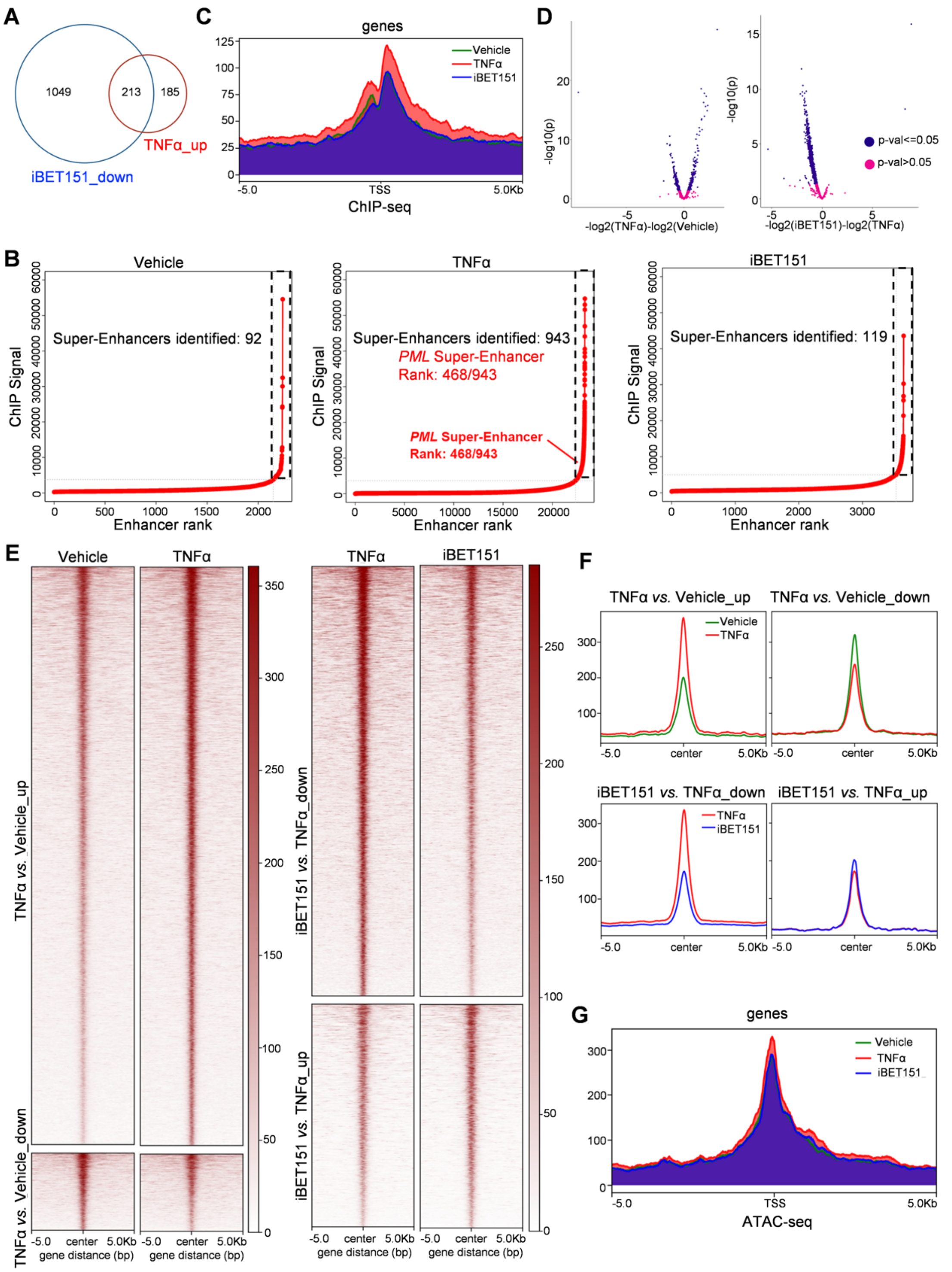

Figure S3. SE-induced PML expression is BRD4 dependent. RNA-seq, BRD4 and H3K27ac ChIP-seq, ATAC-seq, and Hi-C analyses were performed on hLSECs treated with vehicle, TNFα or TNFα plus a BRD4 inhibitor (iBET151). **(A)** Venn diagram of DEGs identified via RNA-seq analysis. **(B)** For BRD4 ChIP-seq, ROSE algorithm analysis of putative SEs in hLSECs among the three groups. The region enclosed by the black dashed box highlights the sequences with the greatest BRD4 enrichment,

which are identified as putative SEs. Importantly, the *PML* SE was ranked 468th among the 943 total SEs. **(C)** The signal profile plot of H3K27ac ChIP-seq on DEGs for hLSECs treated with vehicle, TNFα, or iBET151. **(D)** H3K27ac ChIP-seq reads mapping around transcription start sites of upregulated genes in hLSECs treated with TNFα and downregulated genes in hLSECs treated with iBET151. **(E-F)** Heatmap (E) and signal profile plot (F) of ATAC-seq data (±5 kb from the peak center) for hLSECs treated with vehicle, TNFα, or iBET151. **(G)** The signal profile plot of ATAC-seq data for DEGs in hLSECs treated with vehicle, TNFα, or iBET151.

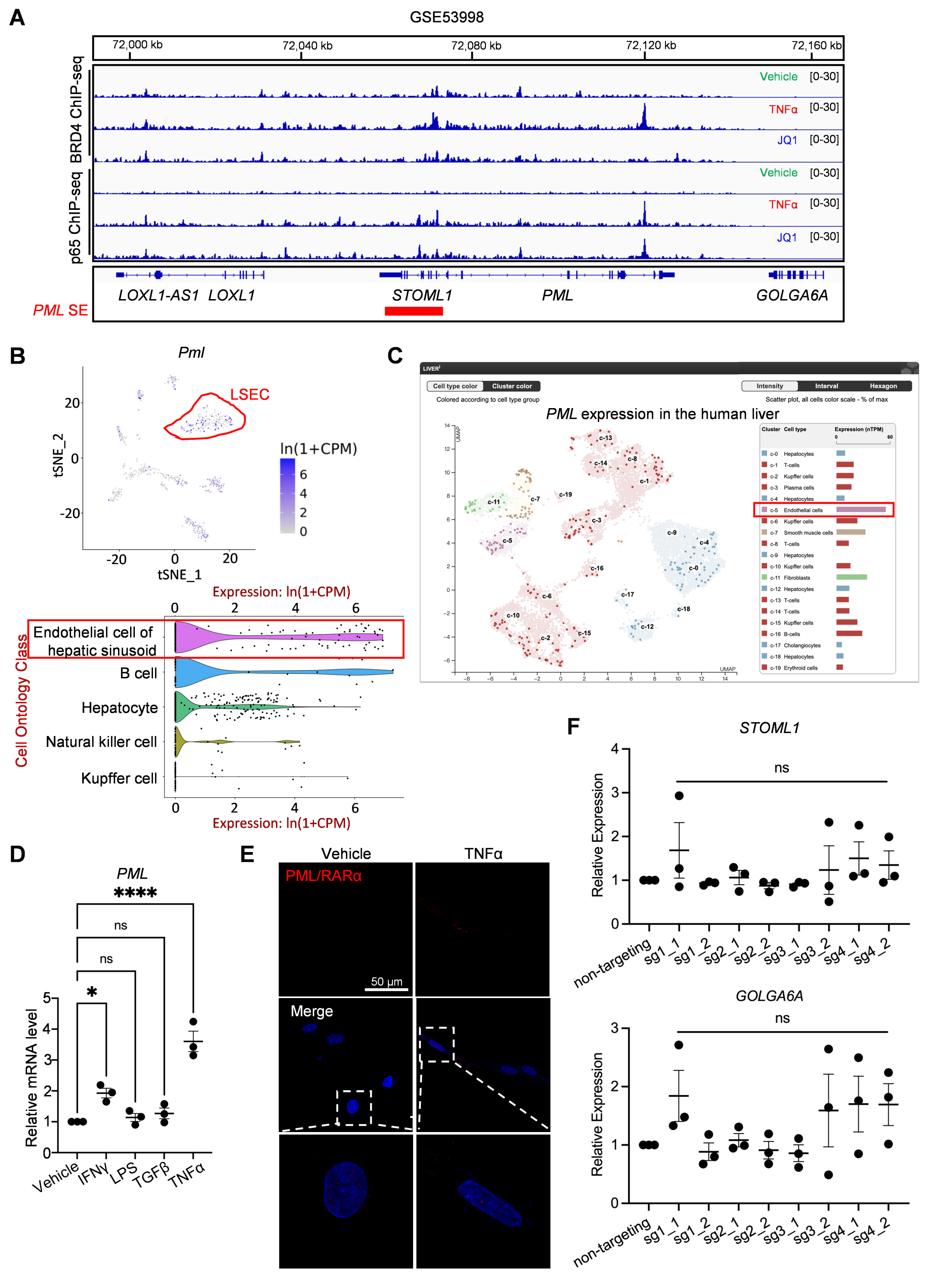

Figure S4. LSECs are the major cell type expressing PML in the human and mouse liver. (A) Published BRD4 and p65 ChIP-seq analysis revealed that the SE of *PML* in HUVECs was regulated by BRD4 (GSE53998). **(B-C)** Published scRNA-seq analysis revealed that LSECs are the primary cell type expressing PML in healthy livers from mice (https://tabula-muris.ds.czbiohub.org, B) and humans (https://www.proteinatlas.org/ENSG00000140464-PML/single+cell/liver, C). **(D)**

qPCR analysis of the *PML* gene in hLSECs treated with a panel of inflammatory stimuli (IFNγ, LPS, TGFβ, and TNFα) (n=3, representing 3 independent experiments). **(E)** Representative IF staining of PML/RARα in hLSECs treated with vehicle or TNFα. **(F)** sgRNAs targeting different sites of *PML* SE were transduced into hLSECs expressing dCas9-KRAB, and qPCR analysis of *PML*-near genes (*STOML1* and *GOLGA6A*) was performed. Human umbilical vein endothelial cells, HUVECs. *****P*<0.0001, and **P*<0.05. ns, not significant. The data are presented as the means ± SEMs. One-way ANOVA with Tukey's multiple comparison test was used.

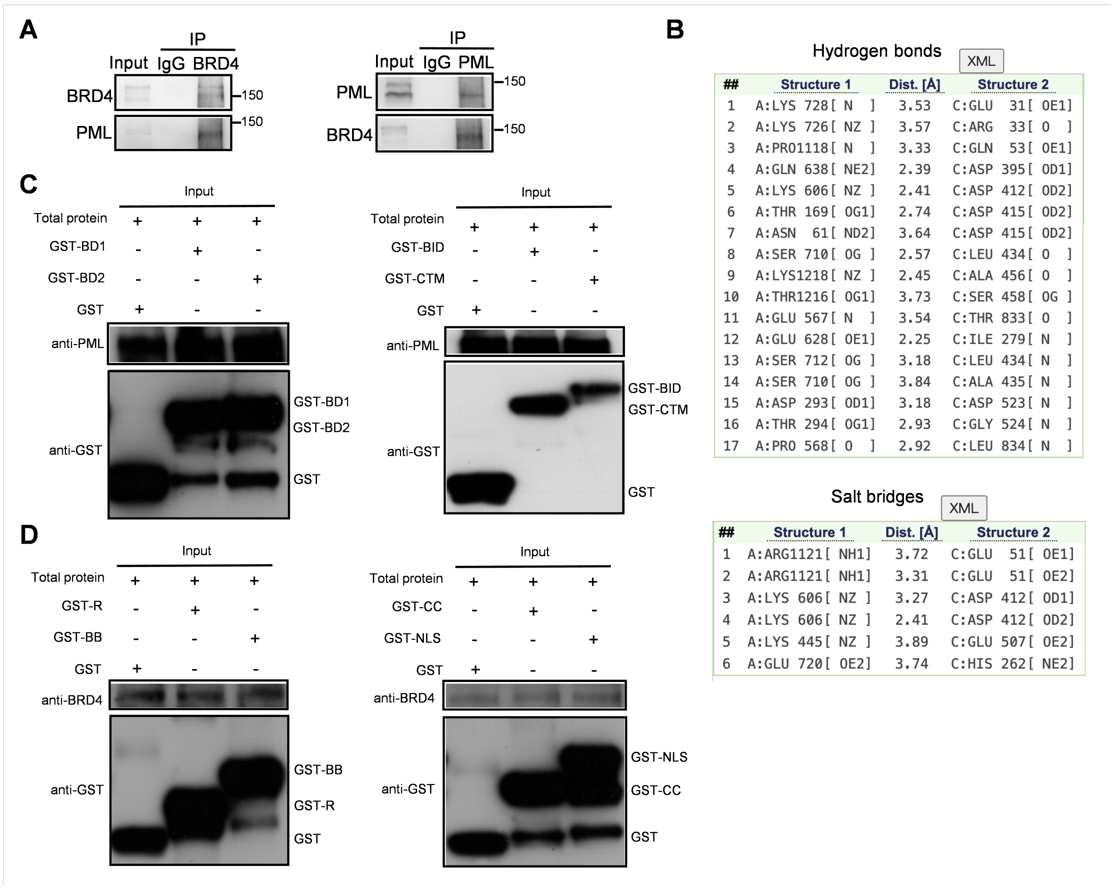

Figure S5. PML directly binds BRD4 in the nucleus. (A) A lentivirus was transduced into hLSECs to overexpress the BRD4 or PML genes. Reciprocal IP and IB analyses revealed the associations between exogenous BRD4 and PML. **(B)** The PML and BRD4 proteins interact via hydrogen bonds and salt bridges at the interface. Structure 1 is BRD4, and structure 2 is PML. **(C)** IB analysis of GST-tagged BRD4 domains with total protein (related to Fig. 3G). **(D)** IB analysis of GST-tagged PML domains with

total protein (related to Fig. 3I).

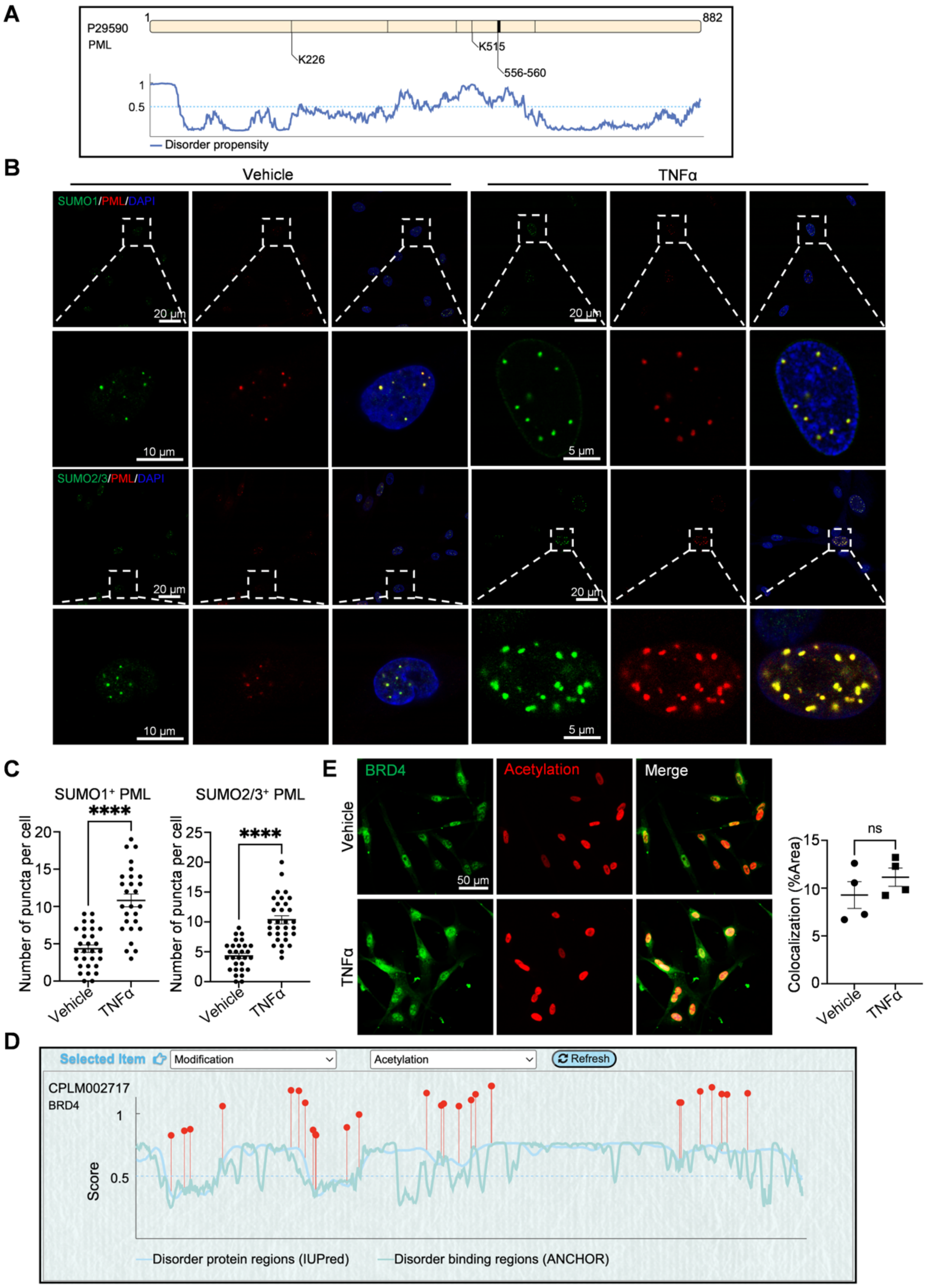

Figure S6. Inflammatory stimuli induce posttranslational modifications of PML in hLSECs. (A) In silico prediction of conserved SUMOylation motifs in human PML (GPS-SUMO 2.0). **(B-C)** Costaining (B) and quantification (C) of SUMO1 or SUMO2/3 with PML in hLSECs treated with vehicle or TNFα. **(D)** Putative acetylation

sites in human BRD4 (compendium of protein lysine modifications, CPLM). **(E)** Costaining and quantification of BRD4 with acetylated lysine in hLSECs treated with vehicle or TNFα. ****P<0.0001; ns, not significant. The data are presented as the means ± SEMs. Two-tailed Student's t test for (C) and (E).

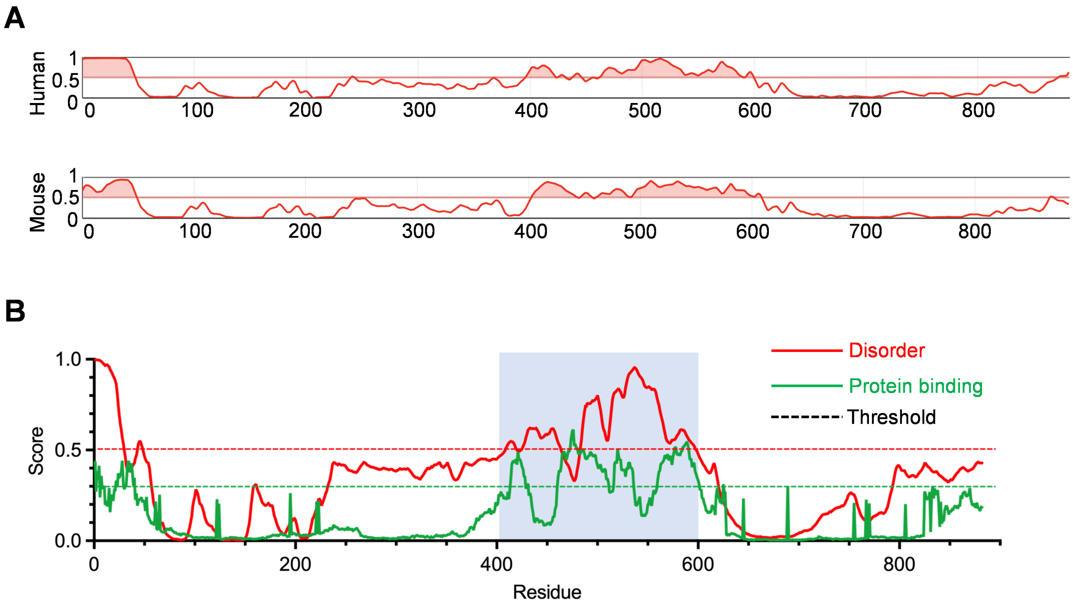

Figure S7. Prediction of intrinsically disordered regions (IDRs) and protein binding regions of the PML protein. (A) Publicly available database showing the IDR regions of the PML protein in humans and mice (https://iupred3.elte.hu/plot). **(B)** Prediction of the IDR region (red) and protein binding region (green) of the PML protein (http://biomine.cs.vcu.edu/servers/hybridPBRpred/). The box shows that amino acids 400--600 are predicted to be disordered and protein-binding regions.

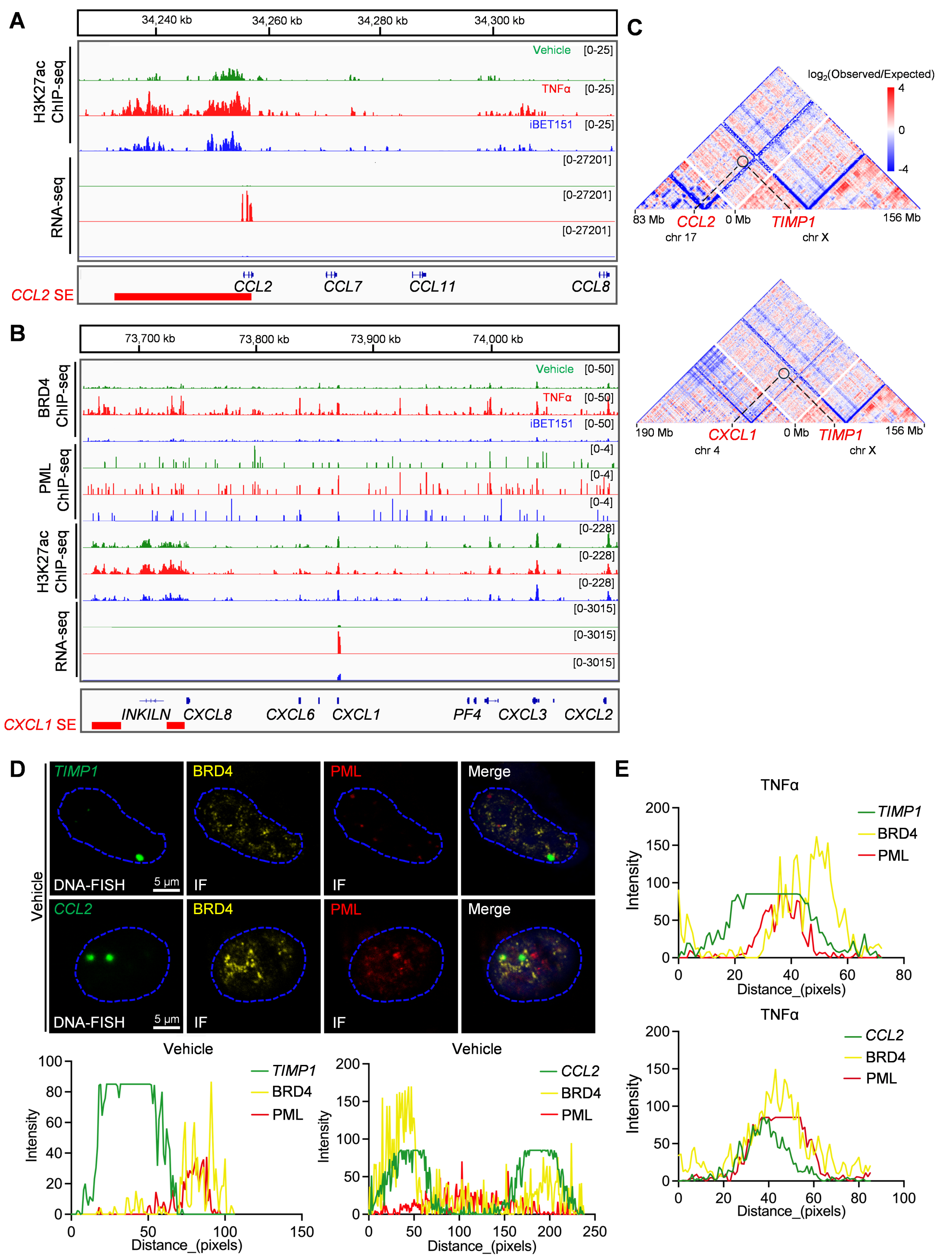

Figure S8. SE-induced chemokine expression is BRD4 dependent. RNA-seq, ChIP-seq, and Hi-C analyses were performed on hLECs treated with TNFα and a BRD4 inhibitor (iBET151). **(A)** ChIP-seq analysis showing H3K27ac intensity and SE activity at the *CCL2* locus in hLSECs, alongside the RNA-seq signal for *CCL2* expression. **(B)** ChIP-seq analysis of H3K27ac, BRD4 and PML intensity, as well as SE activity at the *CXCL1* locus in hLSECs, accompanied by the RNA-seq signal for *CXCL1* expression.

(C) Hi-C heatmaps showing increased interchromosomal interactions between the *TIMP1* and *CCL2* genes (upper panel), as well as between the *TIMP1* and *CXCL1* genes (lower panel), in TNFα-treated hLSECs. **(D)** Representative IF staining for PML and BRD4 combined with DNA-FISH for the *TIMP1* or *CCL2* gene in vehicle-treated hLSECs and the corresponding quantification. **(E)** Quantification of DNA-FISH data from Fig. 4K.

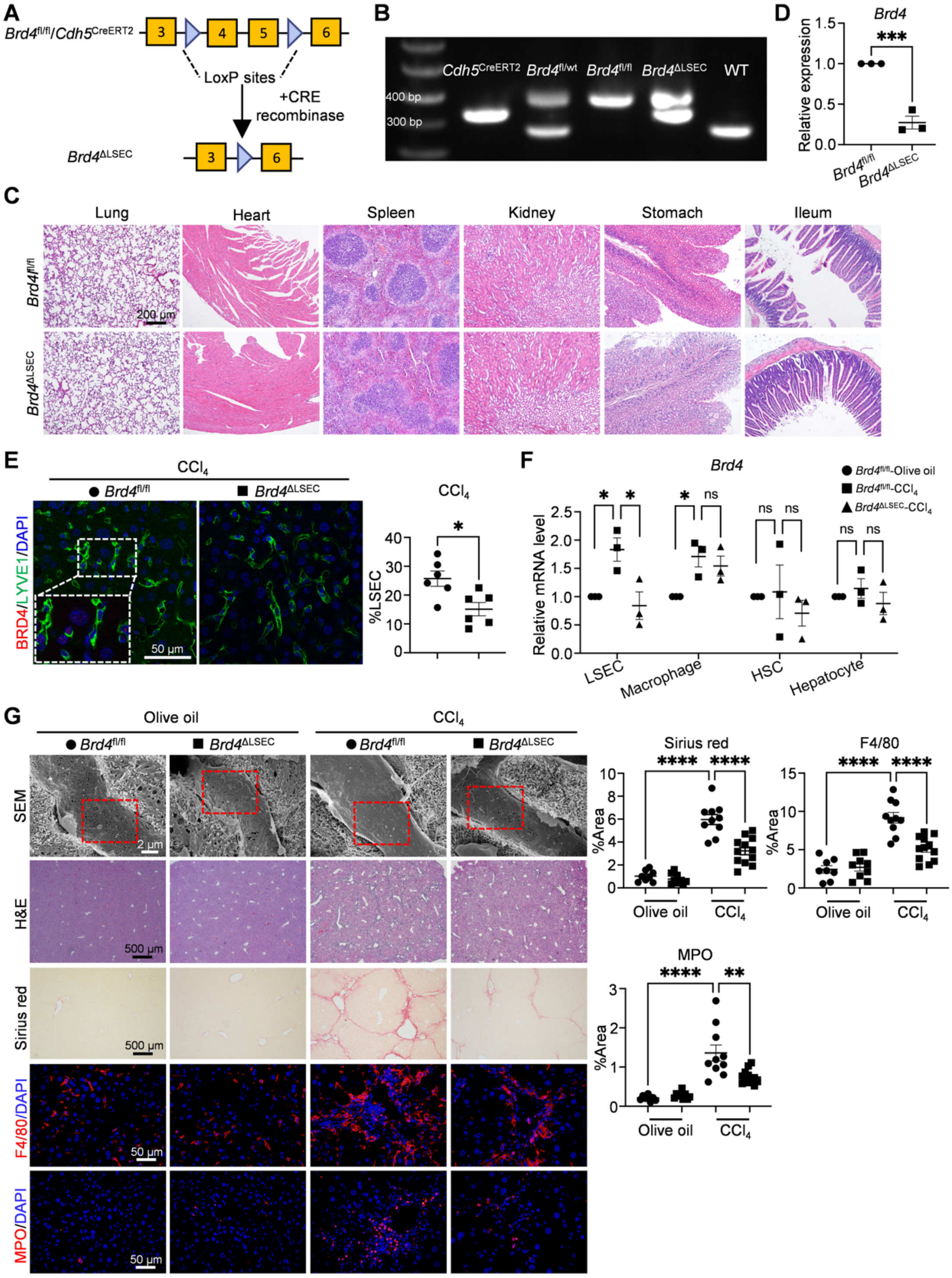

Figure S9. LSEC-specific *Brd4* deletion attenuates liver inflammation and fibrosis in a CCl_4-induced mouse model. (A) Schematic of *Brd4* knockout (*Brd4*$^{fl/fl}$/*Cdh5*^{CreERT2}) alleles. Lines indicate introns; boxes indicate exons. *Brd4* knockout was achieved in an inducible Cre-dependent manner. **(B)** Agarose gel electrophoresis of PCR products revealing the genotyping of *Cdh5*^{CreERT2}, *Brd4*$^{fl/wt}$, *Brd4*$^{fl/fl}$, *Brd4*$^{fl/fl}$/*Cdh5*^{CreERT2} (*Brd4*$^{\Delta LSEC}$), and wild-type (WT) mice. **(C)** Representative

H&E staining of tissue sections (lung, heart, spleen, kidney, stomach and ileum) from *Brd4*$^{fl/fl}$ and *Brd4*$^{\Delta LSEC}$ mice (n=2/group). **(D)** qPCR analysis of *Brd4* gene expression in LSECs isolated from *Brd4*$^{fl/fl}$ and *Brd4*$^{\Delta\ LSEC}$ after tamoxifen injection (n=3/group). **(E)** Costaining of BRD4 and LYVE1 in liver sections from *Brd4*$^{fl/fl}$ and *Brd4*$^{\Delta LSEC}$ mice i.p. injected with CCl_4 (n=6/group). The quantification (right panel) shows the percentage of BRD4 expression in endothelial cells. **(F)** qPCR analysis of the *Brd4* gene in primary liver cells isolated from *Brd4*$^{fl/fl}$ and *Brd4*$^{\Delta LSEC}$ mice treated with olive oil or CCl_4 (n=3/group). **(G)** Representative H&E, Sirius red, F4/80 and MPO staining of liver sections, as well as scanning electron microscopy (SEM) images of liver sinusoids from *Brd4*$^{fl/fl}$ and *Brd4*$^{\Delta LSEC}$ mice subjected to i.p. injection of olive oil (n=8–9/group) or CCl_4 (n=10–12/group). The quantification of SEM, Sirius red, F4/80 and MPO is shown on the right. The panels of SEM from Fig. 5A are cropped from the red dashed boxes. ****$P<0.0001$, ***$P<0.001$, **$P<0.01$, and *$P<0.05$. ns, not significant. The data are presented as the means ± SEMs; two-tailed Student's *t* test (D-E) and one-way ANOVA with Tukey's multiple comparison test (F-G).

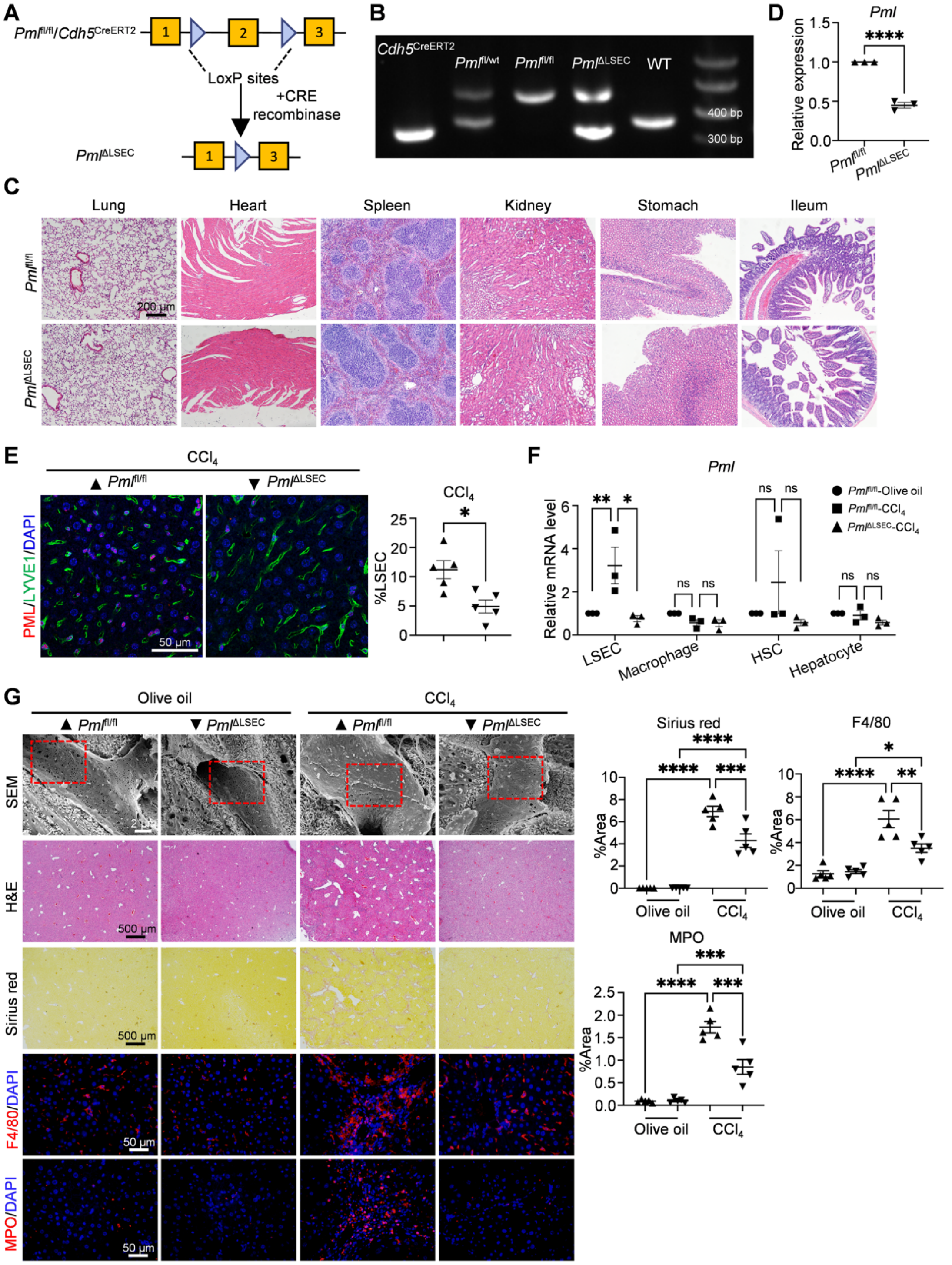

Figure S10. LSEC-specific *Pml* deletion ameliorated liver inflammation and fibrosis in a CCl_4-induced mouse model. (A) Schematic of *Pml* knockout (*Pml*$^{fl/fl}$/*Cdh5*^{CreERT2}) alleles. Lines indicate introns; boxes indicate exons. *Pml* knockout was achieved in an inducible Cre-dependent manner. **(B)** Agarose gel electrophoresis of PCR products revealing the genotyping of *Cdh5*^{CreERT2}, *Pml*$^{fl/wt}$, *Pml*$^{fl/fl}$, *Pml*$^{fl/fl}$/*Cdh5*^{CreERT2} (*Pml*$^{\Delta LSEC}$) and WT mice. **(C)** Representative H&E staining of tissue

sections (lung, heart, spleen, kidney, stomach and ileum) from *Pml*$^{fl/fl}$ and *Pml*$^{\Delta LSEC}$ mice (n=2/group). **(D)** qPCR analysis of *Pml* gene expression in LSECs isolated from *Pml*$^{fl/fl}$ and *Pml*$^{\Delta\ LSEC}$ after tamoxifen injection (n=3/group). **(E)** Costaining of PML and LYVE1 in liver sections from *Pml*$^{fl/fl}$ and *Pml*$^{\Delta LSEC}$ mice i.p. injected with CCl_4 (n=5/group). The quantification (right panel) shows the percentage of PML expression in endothelial cells. **(F)** qPCR analysis of the *Pml* gene in primary liver cells isolated from *Pml*$^{fl/fl}$ and *pml*$^{\Delta LSEC}$ mice treated with olive oil or CCl_4 (n=3/group). **(G)** Representative images of H&E, Sirius red, F4/80 and MPO staining of liver sections, as well as SEM images of liver sinusoids from *Pml*$^{fl/fl}$ and *Pml*$^{\Delta LSEC}$ mice i.p. injected with olive oil (n=5/group) or CCl_4 (n=5/group). The quantification of SEM, Sirius red, F4/80 and MPO is shown on the right. The panels of SEM from Fig. 5C are cropped from the red dashed boxes.****P<0.0001, ***P<0.001, **P<0.01, and *P<0.05. ns, not significant. The data are presented as the means ± SEMs; two-tailed Student's *t* test (D-E) and one-way ANOVA with Tukey's multiple comparison test (F-G).

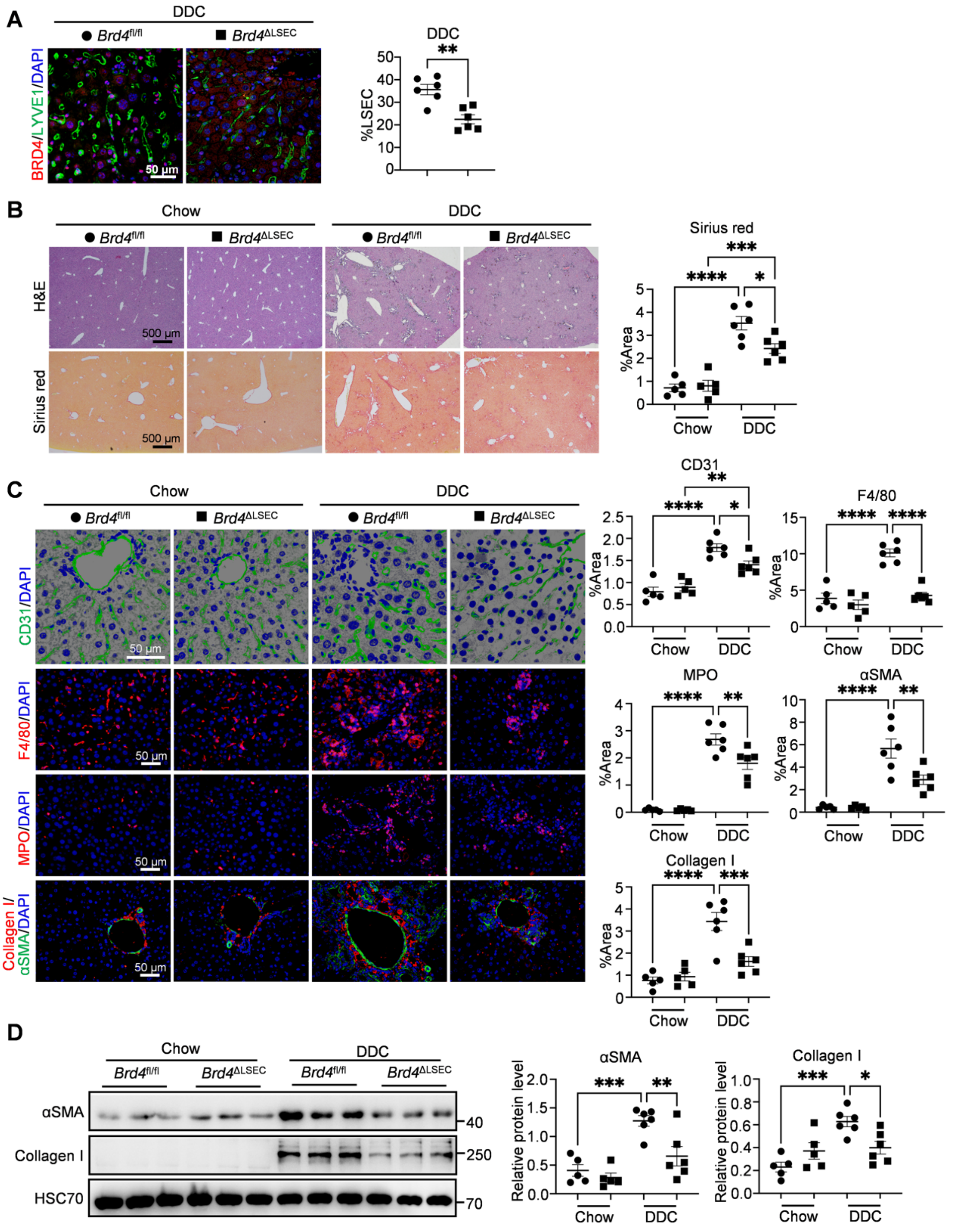

Figure S11. LSEC-specific *Brd4* deletion reduces liver inflammation and fibrosis in a DDC-induced mouse model. *Brd4*$^{fl/fl}$ and *Brd4*$^{\Delta LSEC}$ mice were fed a chow diet (n=5/group) or DDC diet (n=6/group) for 2 weeks. **(A)** Costaining of BRD4 and LYVE1 in liver sections from *Brd4*$^{fl/fl}$ and *Brd4*$^{\Delta LSEC}$ mice fed a DDC diet. The quantification (right panel) shows the percentage of BRD4 expression in endothelial cells. **(B)** Representative H&E and Sirius red staining of liver sections from mice fed a DDC diet.

The quantification of Sirius red is shown on the right. **(C)** Representative IF staining and quantification of CD31, F4/80, MPO, and collagen I/αSMA in liver sections. **(D)** Western blot analysis and quantification of collagen I and αSMA in mouse livers. ****P<0.0001, ***P<0.001, **P<0.01, and *P<0.05. The data are presented as the means ± SEMs; two-tailed Student's t test (A) and one-way ANOVA with Tukey's multiple comparison test (B-D).

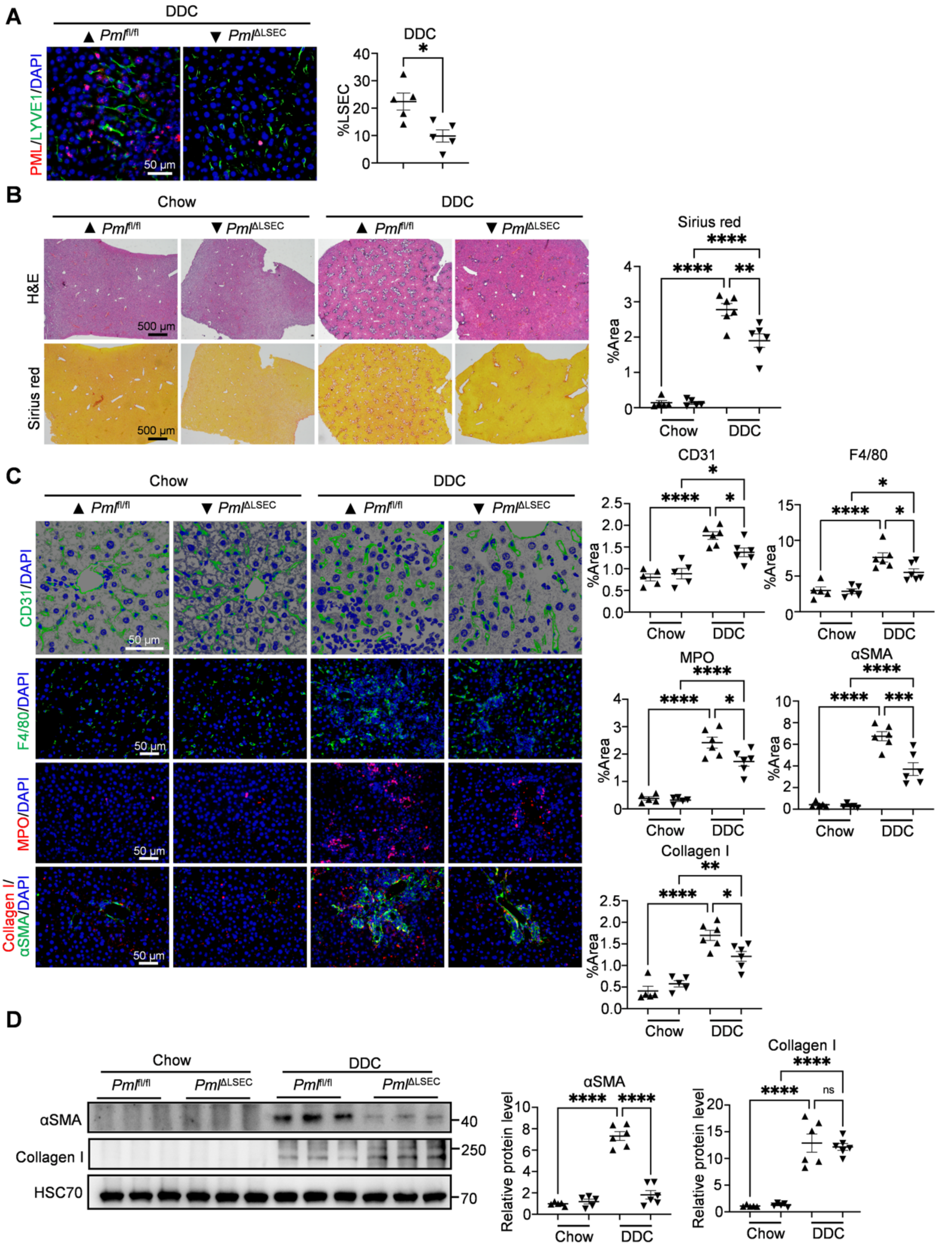

Figure S12. LSEC-specific *Pml* deletion suppresses liver inflammation and fibrosis in a DDC-induced mouse model. *Pml*$^{fl/fl}$ and *Pml*$^{\Delta LSEC}$ mice were fed a chow diet (n=5/group) or DDC diet (n=6/group) for 2 weeks. **(A)** Costaining of PML and LYVE1 in liver sections from *Pml*$^{fl/fl}$ and *Pml*$^{\Delta LSEC}$ mice fed a DDC diet. The percentage of PML expression in endothelial cells was quantified. **(B)** Representative H&E and Sirius red staining of liver sections from mice fed a DDC diet. The quantification of Sirius red

is shown on the right. **(C)** Representative IF staining and quantification of CD31, F4/80, MPO, and collagen I/αSMA in liver sections. **(D)** Western blot analysis and quantification of collagen I and αSMA in mouse livers. ****P<0.0001, ***P<0.001, **P<0.01, and *P<0.05. ns, not significant. The data are presented as the means ± SEMs; two-tailed Student's t test (A) and one-way ANOVA with Tukey's multiple comparison test (B-D).

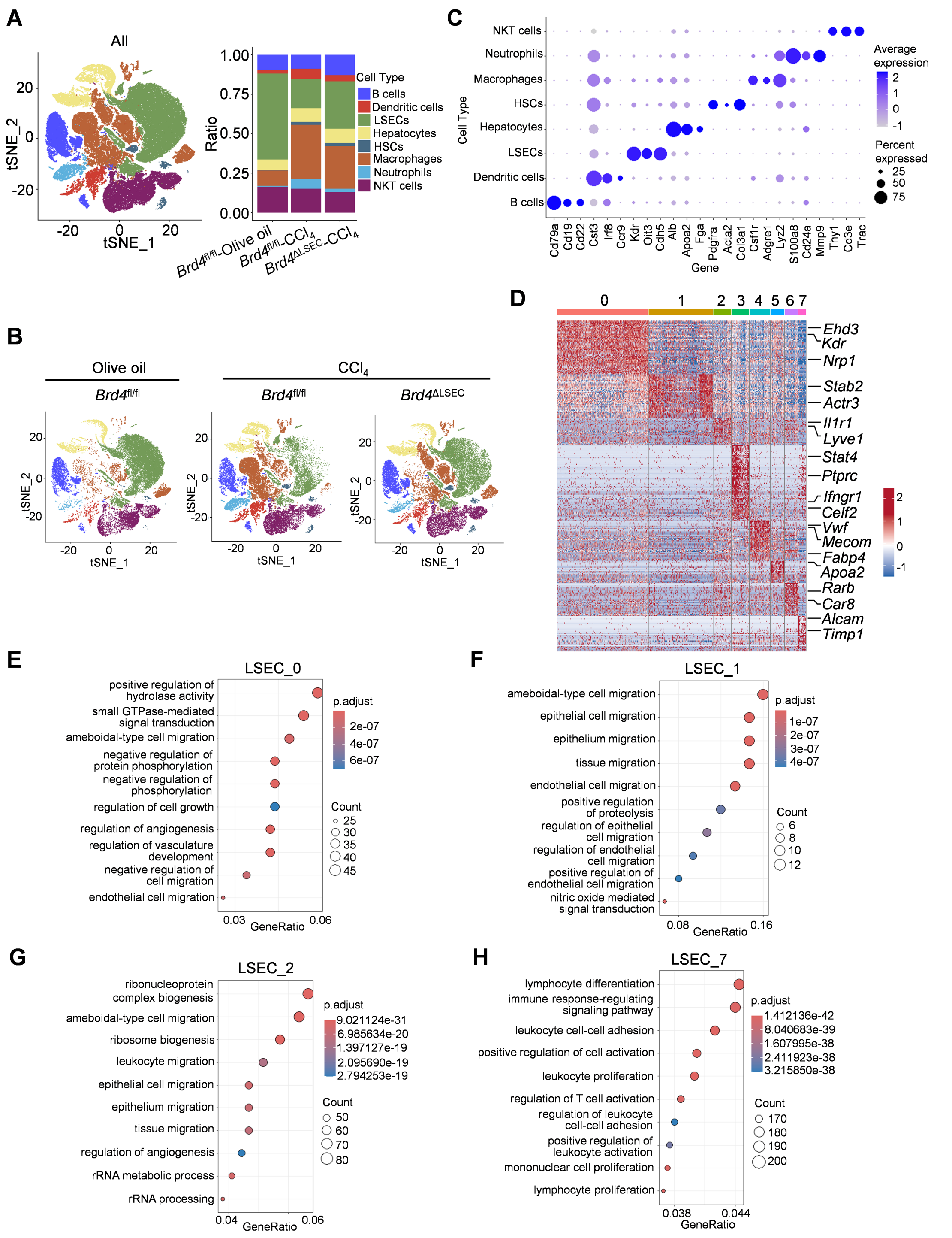

Figure S13. scRNA-seq identifies a proinflammatory endothelial cell landscape. **(A-B)** The tSNE plot of liver cells from *Brd4*$^{fl/fl}$ and *Brd4*$^{\Delta LSEC}$ mice treated with olive oil or CCl_4 revealed 8 different clusters (related to Fig. 6A). Total liver cells and their proportions (A) and liver cells in each group (B). **(C)** Conserved marker genes for liver cell types. **(D)** Heatmap of LSEC clusters with marker genes. **(E-H)** Enriched pathways in LSEC_0, LSEC_1, LSEC_2 and LSEC_7, respectively.

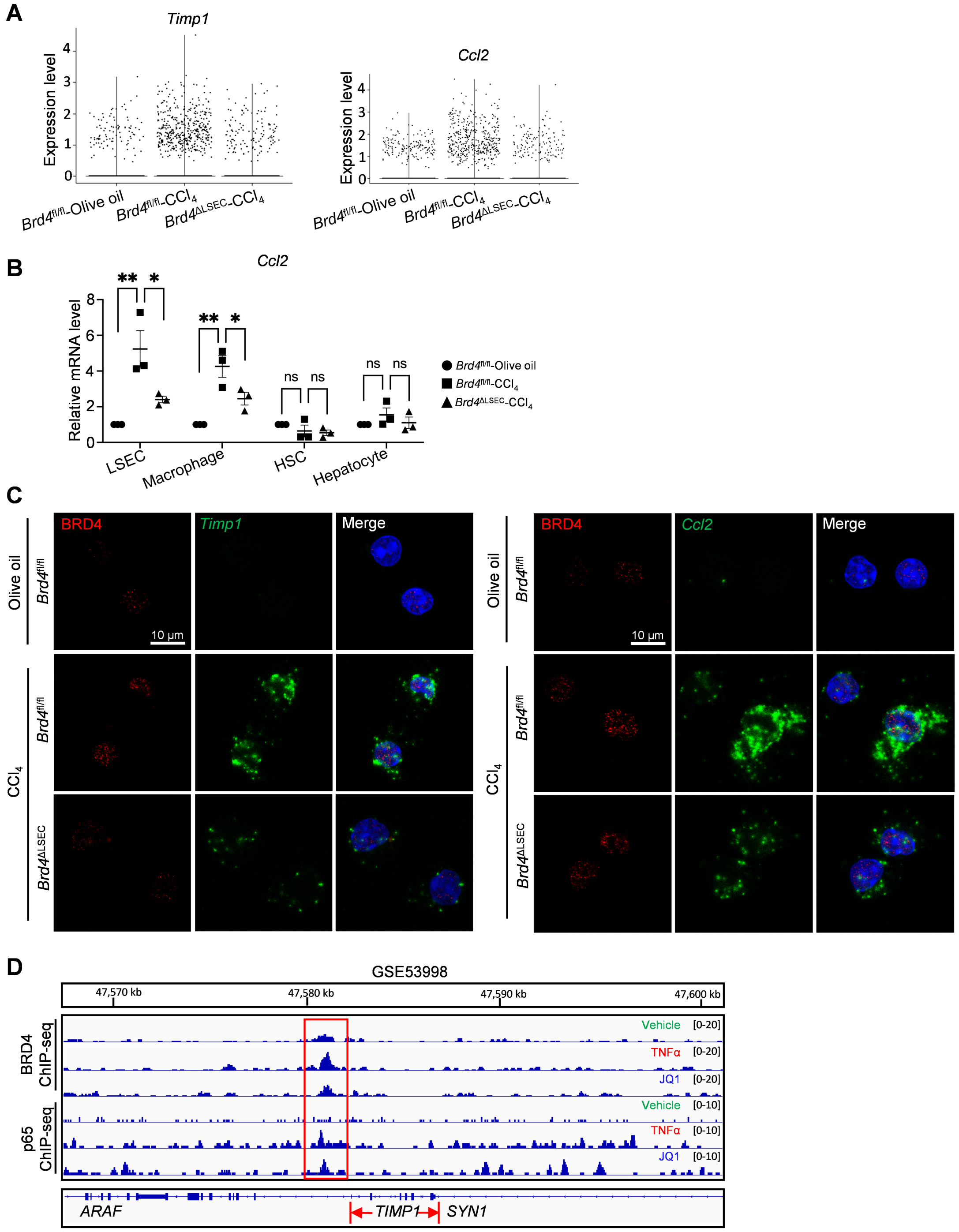

Figure S14. Upregulation of endothelial *Timp1* and *Ccl2* is regulated in a BRD4-dependent manner. (A) *Timp1* and *Ccl2* expression in LSECs among the three groups. **(B)** qPCR analysis of *Ccl2* genes in primary liver cell lines isolated from $Brd4^{fl/fl}$ and $Brd4^{\Delta LSEC}$ mice treated with olive oil or CCl_4 (n=3/group). **(C)** RNA-FISH analysis of *Timp1* (left panel) or *Ccl2* (right panel) probes with BRD4 protein in primary LSECs isolated from $Brd4^{fl/fl}$ and $Brd4^{\Delta LSEC}$ mice treated with olive oil and CCl_4. **(D)** Published BRD4 and p65 ChIP-seq analysis (GSE53998) revealed that the SE activity of the *TIMP1* gene in HUVECs was regulated by BRD4. $^{**}P<0.01$, and $^{*}P<0.05$. ns, not significant. The data are presented as the means ± SEMs; one-way ANOVA with Tukey's multiple comparison test (B).

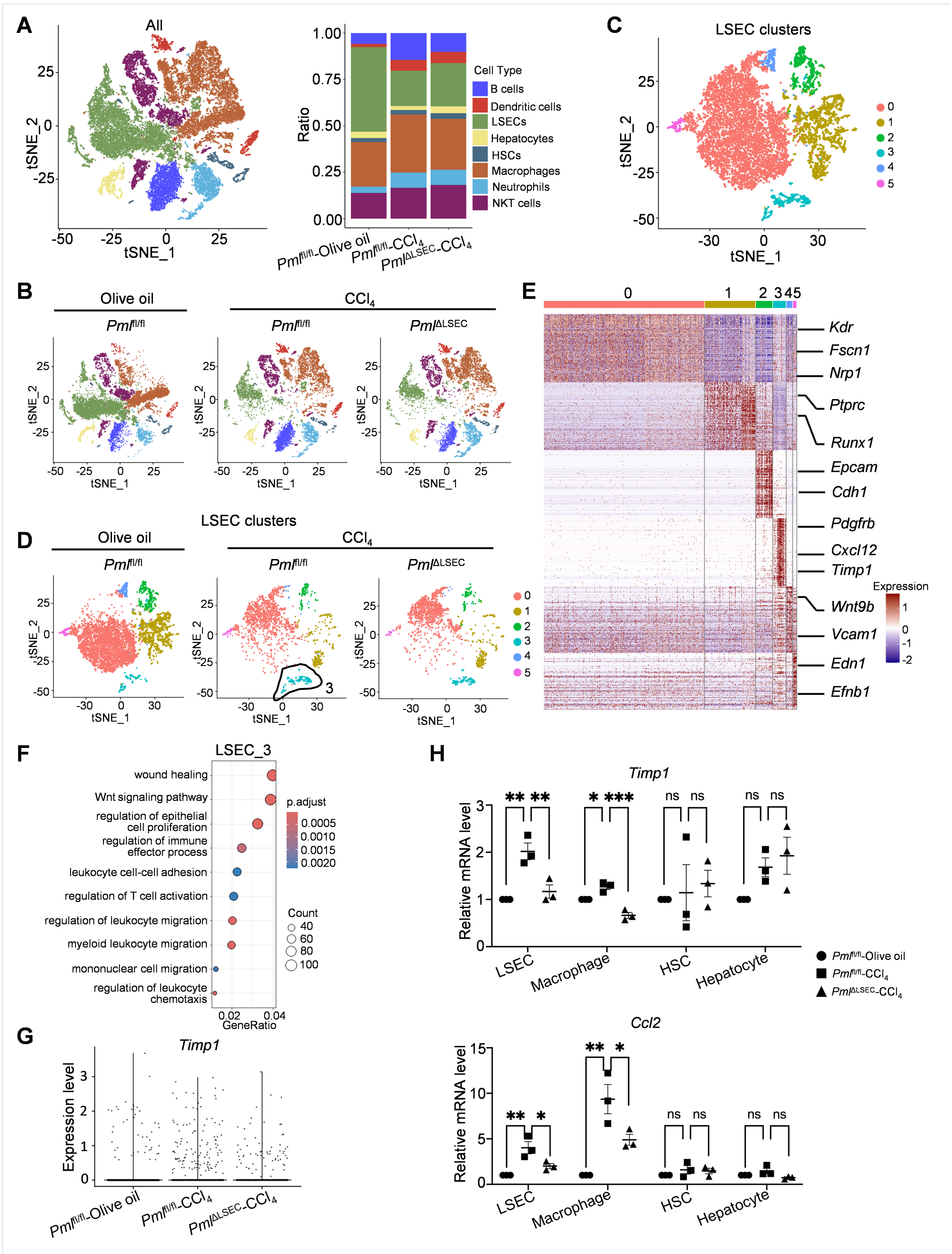

784

785 **Figure S15. scRNA-seq identifies a proinflammatory endothelial cell landscape**

786 **(A-B)** tSNE plot of liver cells from *Pml*$^{fl/fl}$ and *Pml*$^{\Delta LSEC}$ mice treated with olive oil or 787 CCl_4 identified 8 different clusters (n=1/group). Total liver cells and their proportions 788 (A) and liver cells in each group (B). **(C)** The tSNE plot of total LSECs in the three 789 groups. **(D)** The tSNE plot analysis revealing 6 distinct LSEC clusters in the liver. **(E)** 790 Heatmap of LSEC clusters with marker genes. **(F)** Enriched pathways in LSEC_3. **(G)**

Timp1 expression in LSECs among the three groups. **(H)** qPCR analysis of the *Timp1* and *Ccl2* genes in primary liver cell lines isolated from $Pml^{fl/fl}$ and $Pml^{\Delta LSEC}$ mice treated with olive oil or CCl_4 (n=3/group). *****P*<0.0001, ****P*<0.001, ***P*<0.01, and **P*<0.05. ns, not significant. The data are presented as the means ± SEMs; one-way ANOVA with Tukey's multiple comparison test (H).

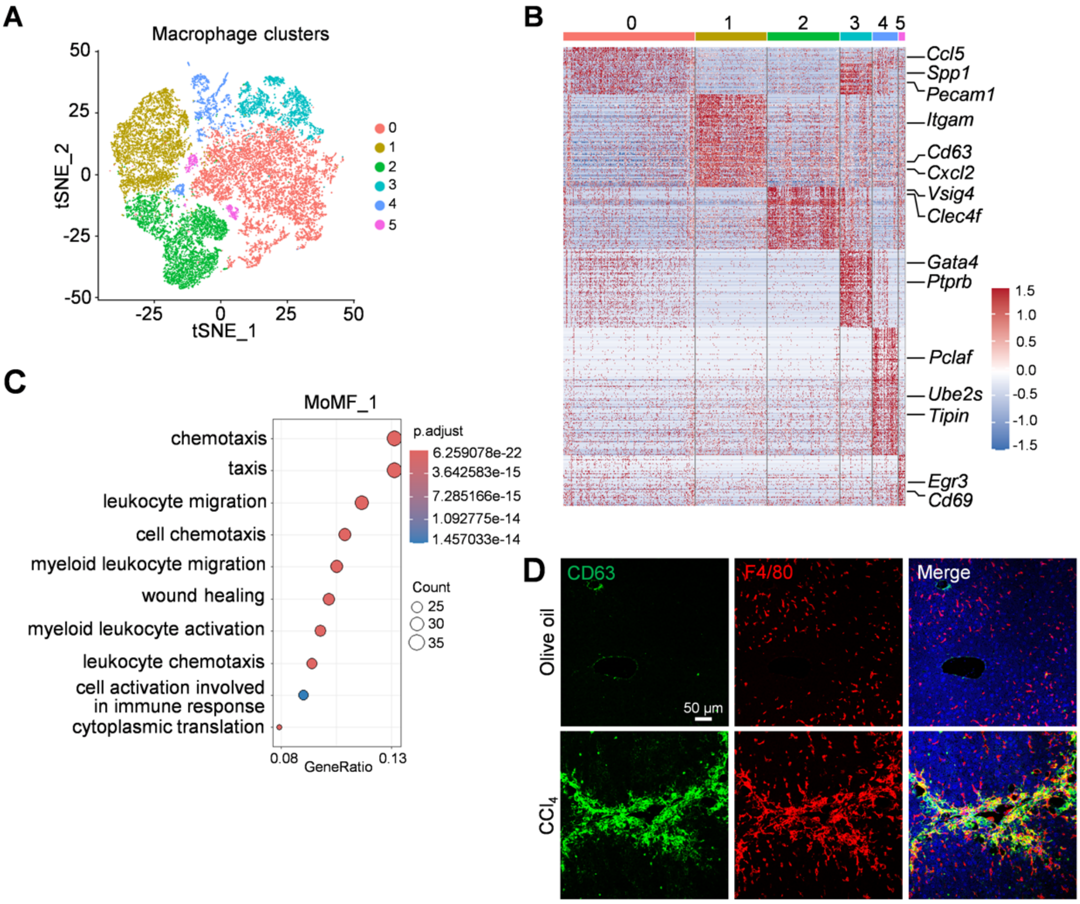

Figure S16. scRNA-seq revealed an increase in inflammatory macrophage infiltration in mouse fibrotic livers. **(A)** The tSNE plot of a total of 6 liver macrophage clusters in $Brd4^{fl/fl}$ and $Brd4^{\Delta LSEC}$ mice treated with olive oil or CCl_4. **(B)** Heatmap of macrophage clusters with marker genes. **(C)** Enriched pathways in macrophage cluster 1 (MoMF_1). **(D)** Representative IF staining of CD63 and F4/80 in mouse liver sections from olive oil- or CCl_4-treated mice.

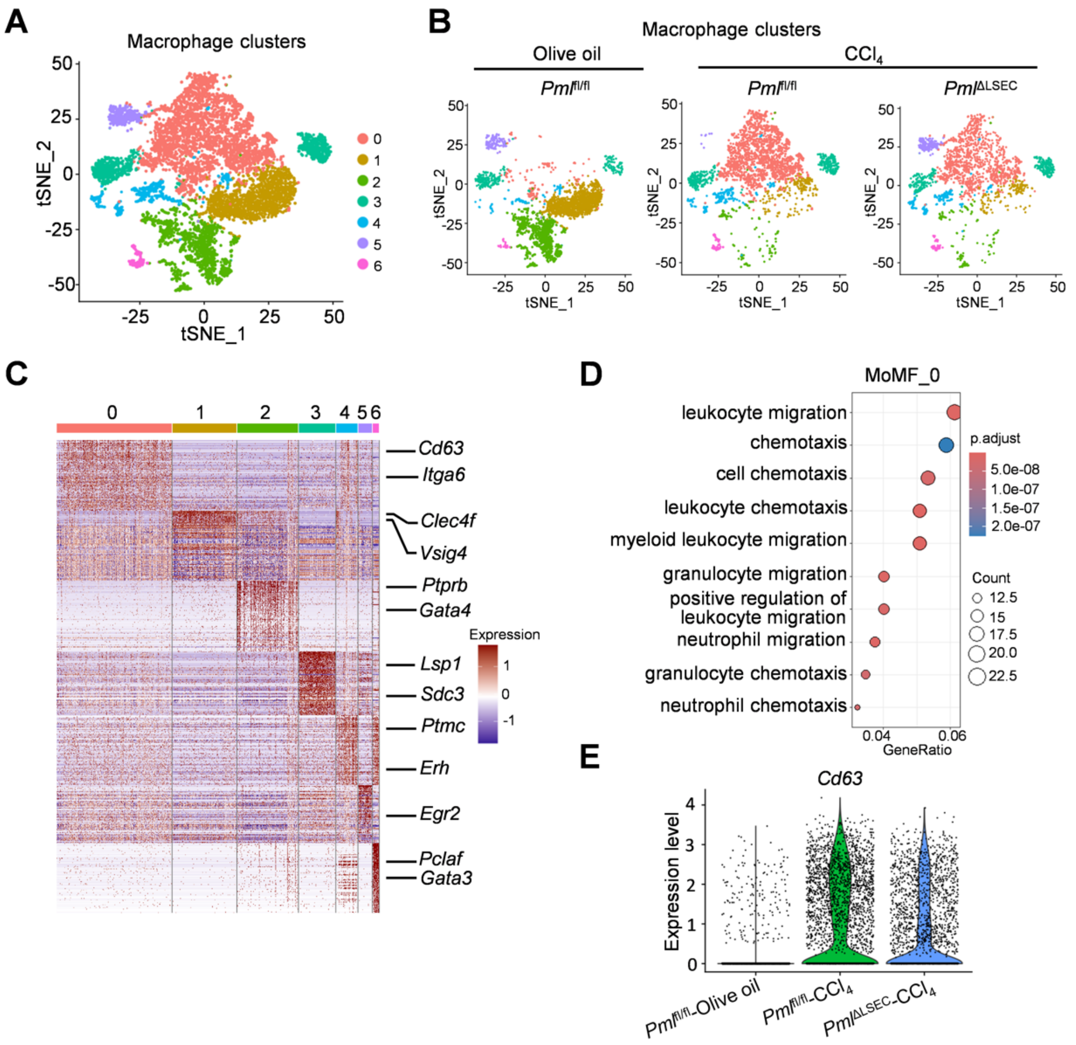

Figure S17. scRNA-seq revealed an increase in inflammatory macrophage infiltration in mouse fibrotic livers. (A-B) The tSNE plot of 7 liver macrophage clusters in *Pml*$^{fl/fl}$ and *Pml*$^{\Delta LSEC}$ mice treated with olive oil or CCl_4. Total macrophages (A) and macrophages in each group (B). **(C)** Heatmap of macrophage clusters with marker genes. **(D)** Enriched pathways in macrophage cluster 0 (MoMF_0). **(E)** *Cd63* expression in macrophages in the three groups.

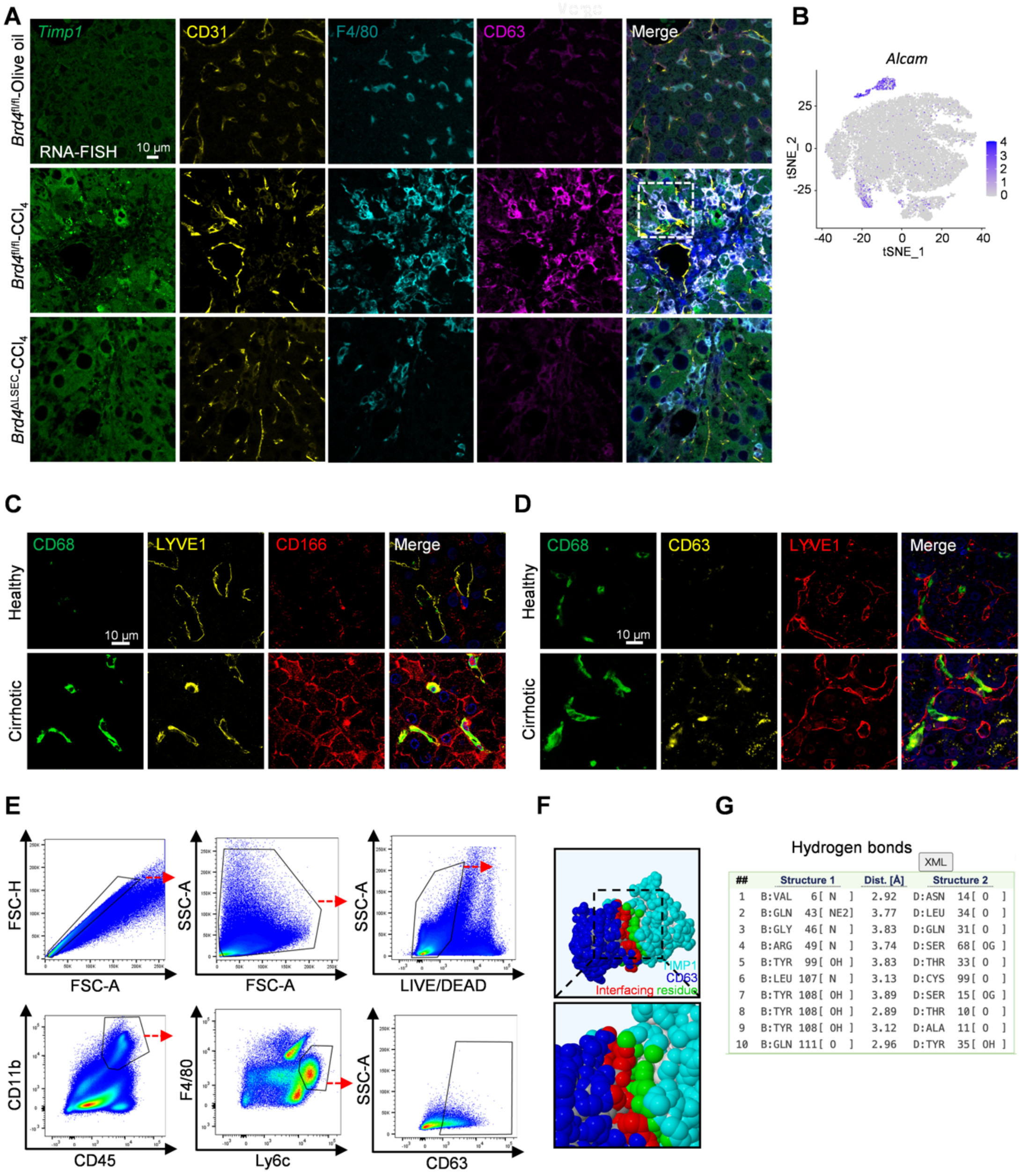

Figure S18. Timp1⁺ LSECs specifically recruit CD63⁺ MoMFs. (A) RNA-FISH analysis of *Timp1* probes and proteins (CD31, F4/80, and CD63) in liver sections from *Brd4*$^{fl/fl}$ and *Brd4*$^{\Delta LSEC}$ mice treated with olive oil or CCl_4 (related to Fig. 7A). **(B)** The tSNE plot of CD166 gene (*Alcam*) expression in LSEC clusters. **(C)** Representative IF staining of CD68, LYVE1 and CD166 in healthy and cirrhotic human livers. **(D)** Representative IF staining of CD68, CD63 and LYVE1 in healthy and cirrhotic human livers. **(E)** Flow cytometry analysis of liver monocyte-derived macrophages (MoMFs). Liver nonparenchymal cells (NPCs) were isolated from olive oil- or CCl_4-treated mice, after which antibodies were used to stain these cells before flow cytometry analysis.

The MoMFs were identified as CD45^{+}, CD11b^{+}, Ly6c^{hi}, and F4/80$^{int/lo}$ cells. **(F)** Molecular docking of the TIMP1-CD63 proteins via the GRAMM-X platform and visualization via the PDBePISA platform. **(G)** TIMP1 and CD63 proteins interact via hydrogen bonds at the interface. Structure 1 is TIMP1, and structure 2 is CD63.

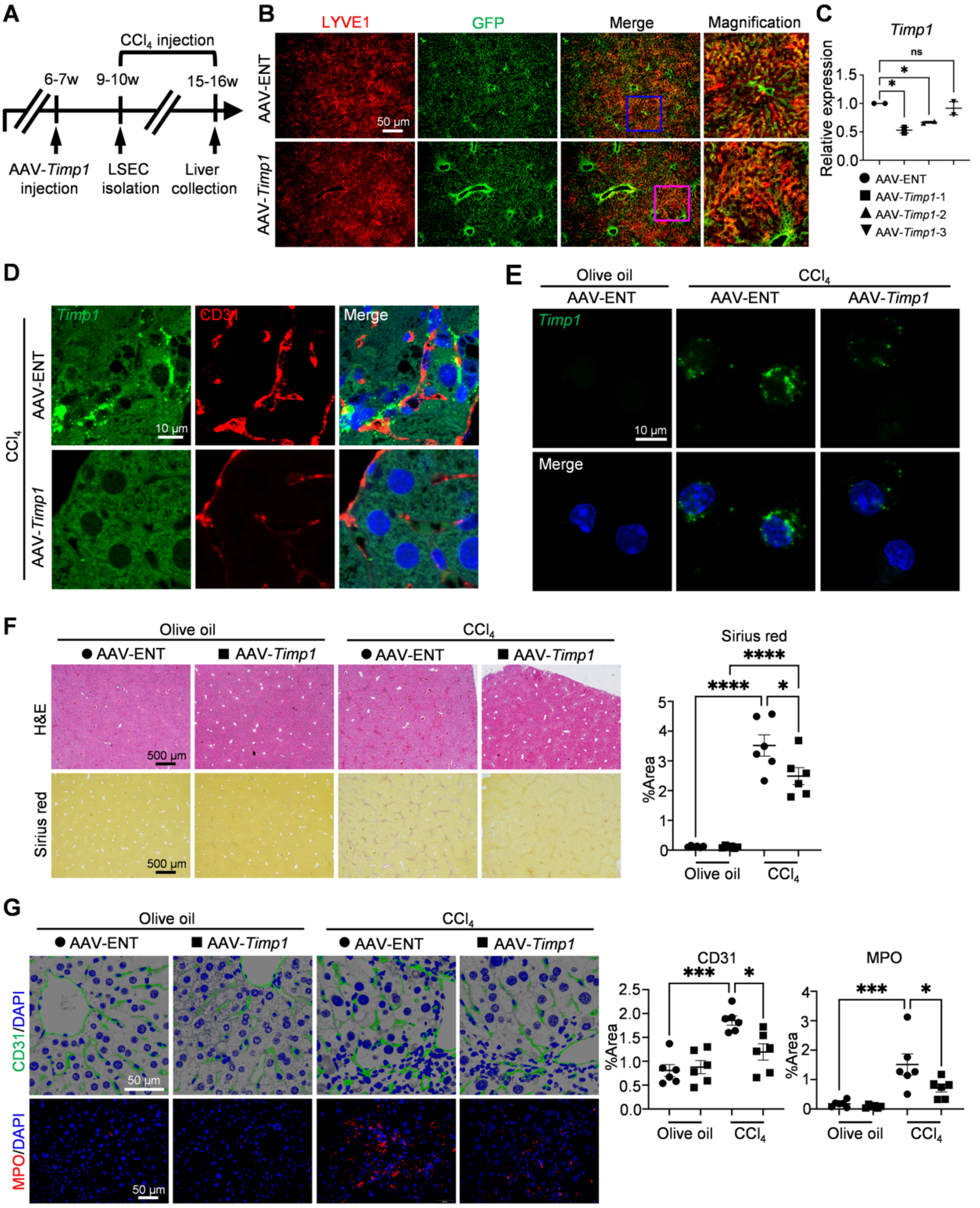

Figure S19. LSEC-specific TIMP1 knockdown decreases liver inflammation and liver fibrosis. (A) Experimental design for the mouse model of AAV-*Timp1* injection

and liver fibrosis. WT mice were pretreated with either AAV-ENT or AAV-*Timp1* via i.v. injection prior to i.p. injection of olive oil or CCl_4. **(B)** Representative IF images of liver sections stained for the LSEC marker LYVE1 and the AAV reporter GFP three weeks after AAV injection. **(C)** LSEC isolation and subsequent qPCR analysis of *Timp1* expression in isolated mouse LSECs were performed 3 weeks after AAV-*Timp1* injection (n=2/group). **(D)** RNA-FISH analysis of the *Timp1* probe with CD31 protein in liver sections from AAV-ENT and AAV-*Timp1* mice treated with CCl_4. **(E)** RNA-FISH analysis of the *Timp1* probe in LSECs isolated from AAV-ENT and AAV-*Timp1* mice treated with olive oil or CCl_4. **(F)** Representative H&E and Sirius red staining of liver sections. The quantification of Sirius red is shown on the right. **(G)** Representative IF staining and quantification of CD31 and MPO in mouse liver sections. ****$P<0.0001$, ***$P<0.001$ and *$P<0.05$. ns, not significant. The data are presented as the means ± SEMs; one-way ANOVA with Tukey's multiple comparison test was used.

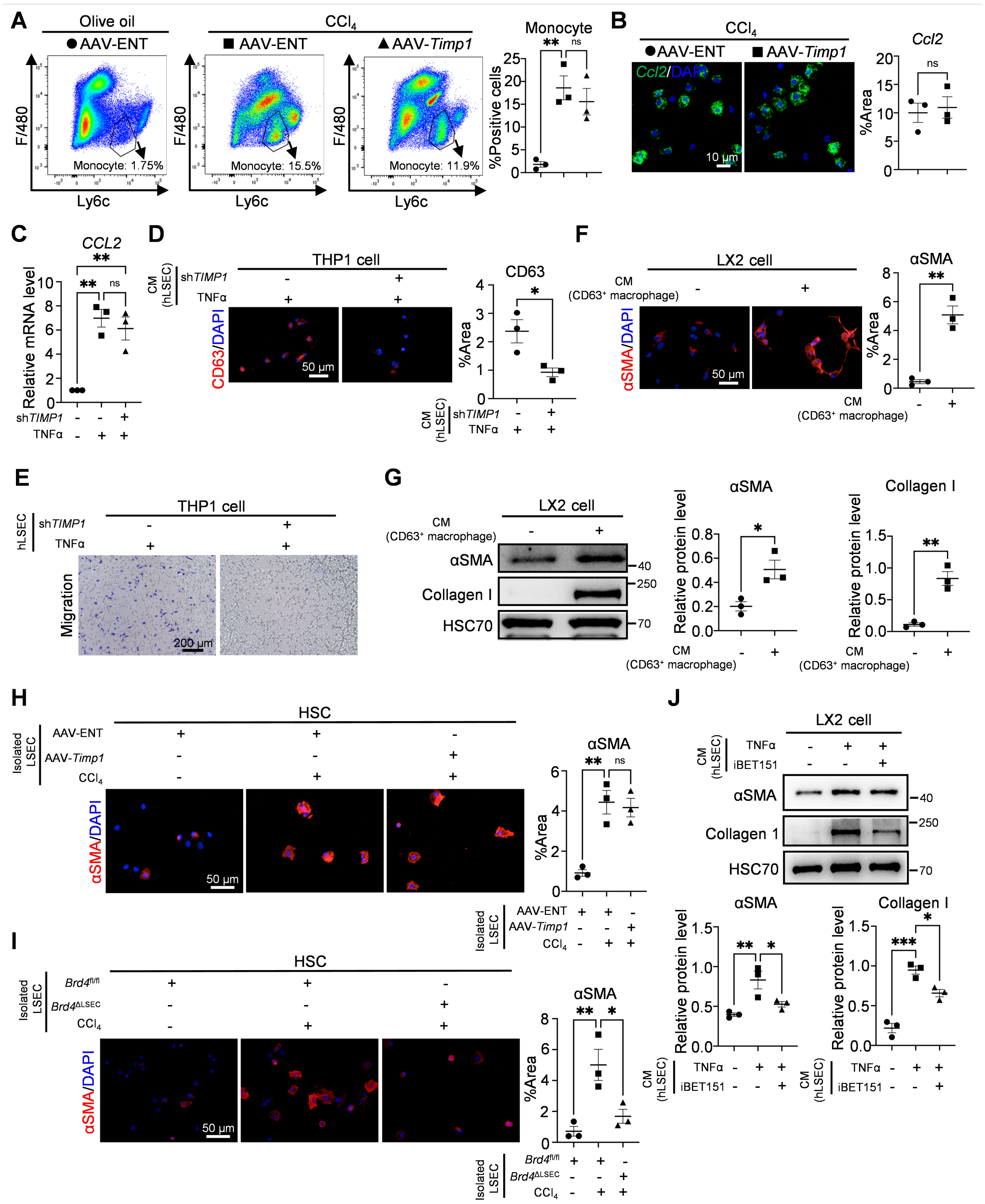

Figure S20. Endothelial TIMP1 induces liver inflammation and HSC activation in a CD63$^+$ MoMF-dependent manner. (A) Flow cytometry analysis and quantification of infiltrating monocytes in livers from AAV-ENT and AAV-*Timp1* mice treated with olive oil or CCl_4 (n=3/group). **(B)** RNA-FISH analysis and quantification of the *Ccl2* probe on LSECs isolated from AAV-ENT and AAV-*Timp1* mice treated with CCl_4 (n=3/group). **(C-E)** hLSECs were transduced with sh*TIMP1* lentivirus and treated with TNFα. The conditioned media (CM) were then collected to treat THP1 cells, or hLSECs were cocultured with THP1 cells (n=3, representing 3 independent experiments). THP1

cells were pretreated with phorbol myristate acetate (PMA) prior to treatment with CM or coculturing with hLSECs. qPCR analysis of the *CCL2* gene in hLSECs (C). Representative IF staining of CD63 in THP1 cells treated with CM from hLSECs (D). Transwell assay of THP1 cells after coculturing with hLSECs (E). **(F-G)** THP1 cells were treated with LPS after exposure to PMA. The conditioned media were then collected to treat LX2 cells (n=3, representing 3 independent experiments). Representative IF staining of αSMA in LX2 cells treated with CM from THP1 cells (F). Western blot analysis and quantification of αSMA and collagen I in LX2 cells (G). **(H)** LSECs isolated from AAV-ENT and AAV-*Timp1* mice treated with olive oil and CCl_4 were cocultured with isolated HSCs from wild-type mice for 12 hours. Representative IF staining and quantification of αSMA in isolated HSCs (n=3, representing 3 independent experiments). **(I)** LSECs isolated from $Brd4^{fl/fl}$ and $Brd4^{\Delta LSEC}$ mice treated with olive oil and CCl_4 were cocultured with isolated HSCs from wild-type mice for 12 hours. Representative IF staining and quantification of αSMA in isolated HSCs (n=3, representing 3 independent experiments). **(J)** hLSECs were treated with TNFα or TNFα plus the BRD4 inhibitor iBET151, after which the CM of hLSECs were collected to treat LX2 cells. Western blot analysis and quantification of αSMA and collagen I in LX2 cells (n=3, representing 3 independent experiments). ***P<0.001, **P<0.01 and *P<0.05. ns, not significant. The data are presented as the means ± SEMs; two-tailed Student's *t* test (B, D, F, G) and one-way ANOVA with Tukey's multiple comparison test (A, C, H, I, J).

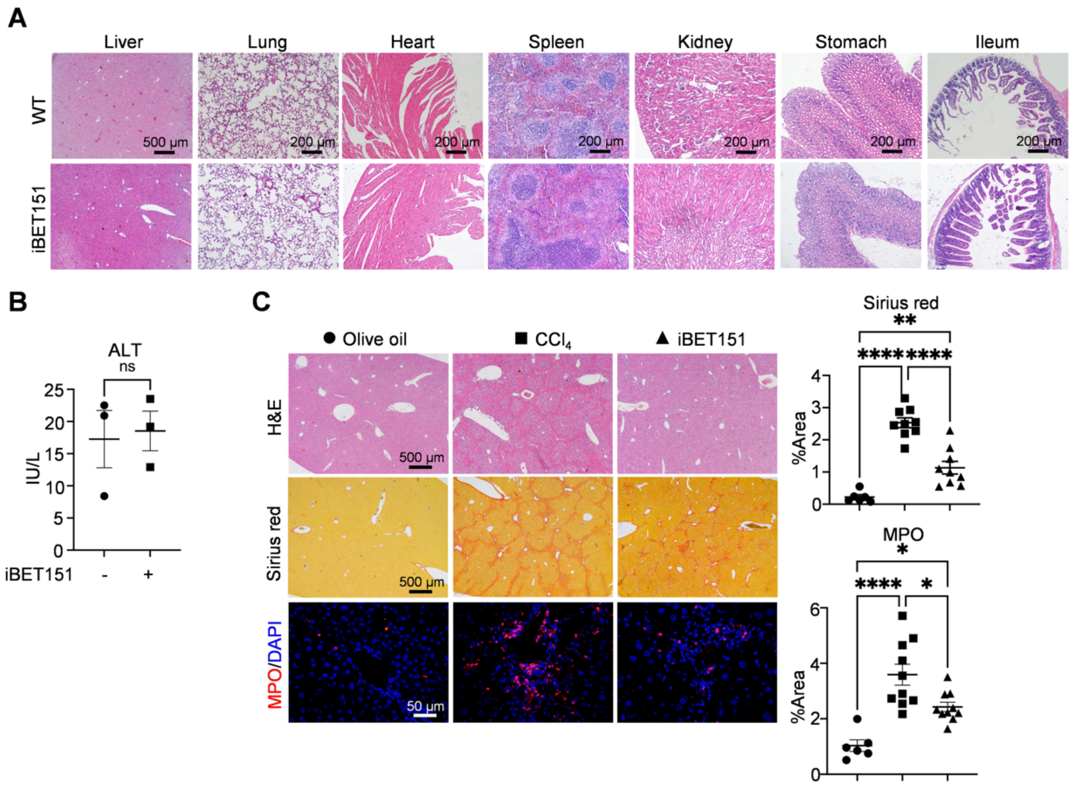

Figure S21. iBET151 treatment inhibits liver inflammation and fibrosis in the absence of systemic toxicity. (A-B) Mice were i.p. injected with iBET151 compound for one week prior to tissue and serum collection. Representative H&E staining of mouse tissue sections (liver, lung, heart, spleen, kidney, stomach and ileum) (A). Serum levels of alanine aminotransferase (ALT) (B). **(C)** Mice were i.p. injected with olive oil, CCl_4 or CCl_4 plus the iBET151 compound (related to Fig. 8A-C). Representative H&E, Sirius red, and MPO staining of mouse liver sections. The quantification of Sirius red and MPO is shown on the right. *****P*<0.0001, ***P*<0.01 and **P*<0.05. ns, not significant. The data are presented as the means ± SEMs; two-tailed Student's *t* test (B) and one-way ANOVA with Tukey's multiple comparison test (C).

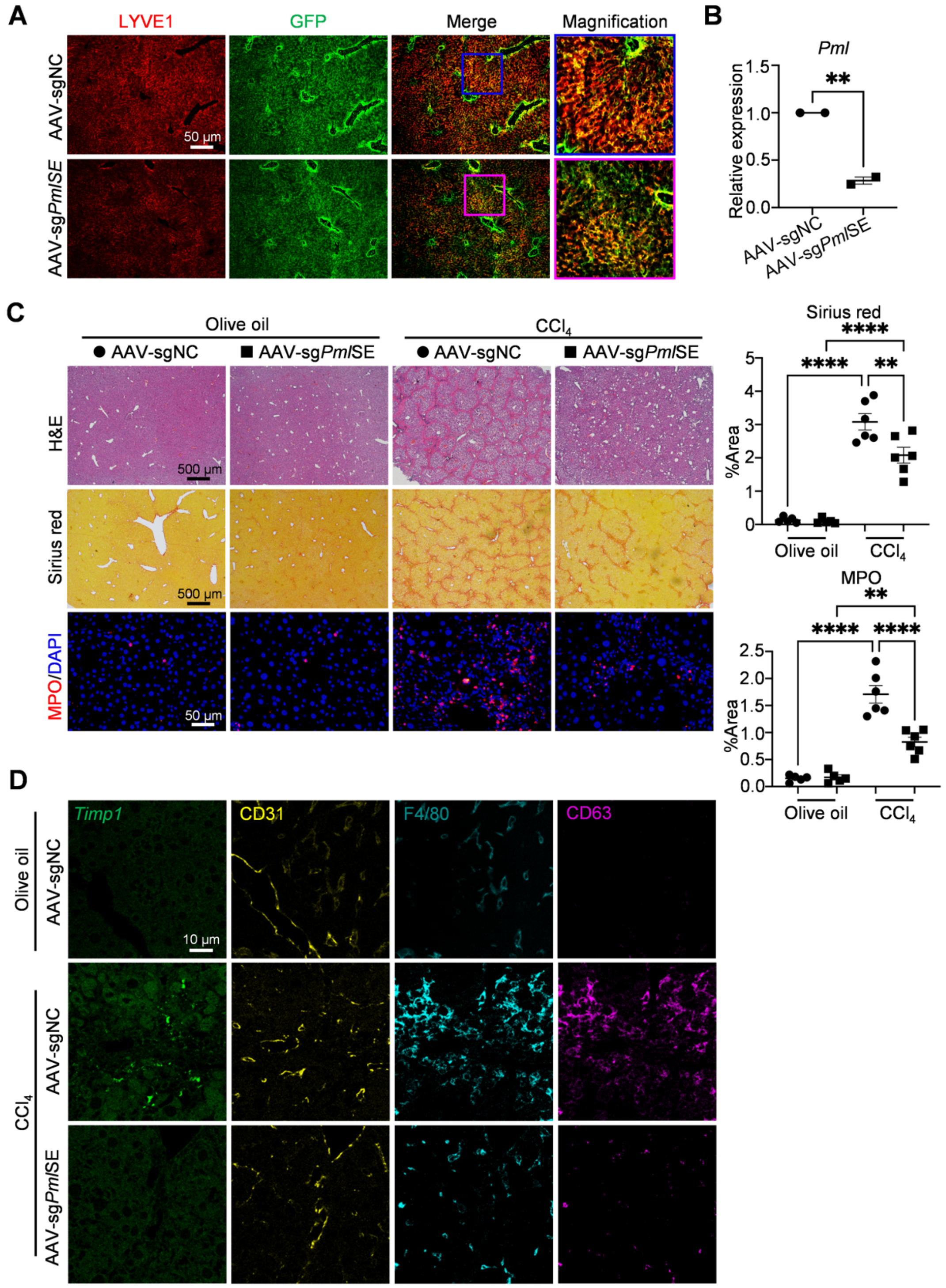

Figure S22. Epigenetic repression of endothelial *Pml* attenuates liver inflammation and fibrosis. (A-D) *dCas9-KRAB*/*Cdh5*^{CreERT2} mice were intravenously injected with AAV-non or AAV-sg*Pml*SE prior to i.p. injection of olive oil or CCl_4. Representative IF images of liver sections stained for the LSEC marker LYVE1 and the AAV reporter GFP two weeks after AAV injection (A). qPCR analysis of *Pml* expression in LSECs isolated from AAV-non and AAV-sg*Pml*SE mice (n=2/group) (B). Representative H&E,

Sirius red, and MPO staining of mouse liver sections. The quantification of Sirius red and MPO is shown on the right (C). **(D)** RNA-FISH analysis of *Timp1* probe and proteins (CD31, F4/80, and CD63) in mouse liver sections (n=3/group) (Related to Fig. 8G). ****$P<0.0001$ and **$P<0.01$. The data are presented as the means ± SEMs; two-tailed Student's *t* test (B) and one-way ANOVA with Tukey's multiple comparison test (C).

Legends for Movies S1 to S5

Movie S1. Portal venography of noncirrhotic and cirrhotic human livers. Portal venography revealed irregular and tortuous hepatic vasculature in human cirrhotic livers.

Movie S2. Intravital imaging of liver sinusoids in mice administered olive oil or CCl_4. DAOSLIMIT revealed sinusoidal distortion and congested blood flow in CCl_4-treated fibrotic mouse livers.

Movie S3. Intravital imaging of F4/80 and Ly6G in mice administered olive oil or CCl_4. DAOSLIMIT revealed increased macrophage (F4/80) and neutrophil (Ly6G) infiltration in CCl_4-treated fibrotic mouse livers.

Movie S4. Intravital imaging of WGA, CD166, and F4/80 in mice administered olive oil or CCl_4. DAOSLIMIT revealed increased macrophage infiltration in proximity to $CD166^+$ endothelial cells in CCl_4-treated $Brd4^{fl/fl}$ livers, a phenomenon not observed in $Brd4^{\Delta LSEC}$ mouse livers. WGA serves as a marker of endothelial cells.

Movie S5. Intravital imaging of WGA, F4/80, and CD63 in mice administered olive oil or CCl_4. DAOSLIMIT indicated increased infiltration of $CD63^+$ macrophages in proximity to endothelial cells in CCl_4-treated $Brd4^{fl/fl}$ livers, whereas this phenomenon was absent in $Brd4^{\Delta LSEC}$ mouse livers.

Supplementary tables

Table S1 Patient information.

Patient No.	Gender	Tissue type	Diagnosis	Analytical Method
#1	F	Control liver	Cholecystolithiasis	IF, WB
#2	M	Control liver	Hepatic hemangioma	IF, WB
#3	F	Control liver	Hepatic hemangioma	IF, WB
#4	F	Control liver	Hepatic hemangioma	IF, WB
#5	M	Control liver	Hepatic cyst	IF, WB
#6	F	Control liver	Hepatic hemangioma	IF, WB
#1	M	Fibrotic liver	HCC	IF
#2	M	Fibrotic liver	HCC	IF
#3	M	Fibrotic liver	HCC	IF
#4	M	Fibrotic liver	HCC	IF
#5	M	Fibrotic liver	HCC	IF
#6	M	Fibrotic liver	HCC	IF
#7	M	Fibrotic liver	HCC	IF
#8	M	Fibrotic liver	HCC	IF
#9	M	Fibrotic liver	HCC	IF
#10	M	Fibrotic liver	HCC	IF
#11	F	Fibrotic liver	HCC	IF
#12	F	Fibrotic liver	HCC	IF
#1	F	Cirrhotic liver	HCC，Cirrhosis	IF, WB
#2	M	Cirrhotic liver	HCC，Cirrhosis	IF, WB
#3	M	Cirrhotic liver	HCC，Cirrhosis	IF, WB
#4	M	Cirrhotic liver	HCC，Cirrhosis	IF, WB
#5	M	Cirrhotic liver	HCC，Cirrhosis	IF, WB
#6	M	Cirrhotic liver	HCC，Cirrhosis	IF, WB
#7	M	Cirrhotic liver	HCC，Cirrhosis	IF
#8	M	Cirrhotic liver	HCC，Cirrhosis	IF
#9	M	Cirrhotic liver	HCC，Cirrhosis	IF
#10	F	Cirrhotic liver	HCC，Cirrhosis	IF
#11	M	Cirrhotic liver	HCC，Cirrhosis	IF
#12	M	Cirrhotic liver	HCC，Cirrhosis	IF
#13	M	Cirrhotic liver	HCC，Cirrhosis	F

F, female; M, male; IF, immunofluorescence; WB, western blot; HCC, hepatocellular carcinoma

Table S2. Histone Modification Compound Library

Compound name	Target	Concentration (μM)
Panobinostat (LBH589)	HDAC	15
Vorinostat (SAHA, MK0683)	Autophagy	15
Entinostat (MS-275)	HDAC	15

Quisinostat (JNJ-26481585) 2HCl	HDAC	15
Valproic acid sodium salt (Sodium valproate)	Autophagy	15
PFI-1 (PF-6405761)	Epigenetic Reader Domain	15
Resveratrol	Autophagy	15
Droxinostat	HDAC	15
MC1568	HDAC	15
Pracinostat (SB939)	HDAC	15
Selisistat (EX 527)	Sirtuin	15
Divalproex Sodium	HDAC	15
Curcumin	Nrf2	15
Nicotinamide (Vitamin B3)	Sirtuin	15
PCI-34051	HDAC	15
Givinostat (ITF2357)	HDAC	15
AR-42	HDAC	15
Fisetin	Sirtuin	15
Tubastatin A HCl	HDAC	15
Resminostat	HDAC	15
M344	HDAC	15
I-BET151 (GSK1210151A)	Epigenetic Reader Domain	15
Sirtinol	Sirtuin	15
Tacedinaline (CI994)	HDAC	15
Entacapone	Histone Methyltransferase	15
Valproic acid	HDAC	15
(-)-Parthenolide	NF-κB	15
Sodium Phenylbutyrate	HDAC	15
Amodiaquine dihydrochloride dihydrate	Histone Methyltransferase	15
Salvianolic acid B	Sirtuin	15
Daminozide	Histone Demethylase	15
Tenovin-6	p53	15
Biphenyl-4-sulfonyl chloride	HDAC	15
AMI-1, free acid	Histone Methyltransferase	15
Sulforaphane	Nrf2	15
AS-8351	Histone Demethylase	15
UF010	HDAC	15
Suberohydroxamic acid	HDAC	15
AK 7	Sirtuin	15
CAY10602	Sirtuin	15
GSK J4 HCl	Histone Demethylase	15
SGC 0946	Histone Methyltransferase	15
(+)-JQ1	Epigenetic Reader Domain	15

C646	Histone Acetyltransferase	15
UNC1999	Histone Methyltransferase	15
RGFP966	HDAC	15
UNC0642	Histone Methyltransferase	15
Bromosporine	Epigenetic Reader Domain	15
IOX1	Histone Demethylase	15
OG-L002	Histone Demethylase	15
SGC-CBP30	Epigenetic Reader Domain	15
MM-102	Histone Methyltransferase	15
HPOB	HDAC	15
JIB-04	Histone Demethylase	15
RG2833 (RGFP109)	HDAC	15
PFI-2 HCl	Histone Methyltransferase	15
ML324	Histone Demethylase	15
CPI-203	Epigenetic Reader Domain	15
MS436	Epigenetic Reader Domain	15
TMP269	HDAC	15
EPZ004777	Histone Methyltransferase	15
Nexturastat A	HDAC	15
MG149	Histone Acetyltransferase	15
LMK-235	HDAC	15
UNC0379	Histone Methyltransferase	15
A-366	Histone Methyltransferase	15
GSK-LSD1 2HCl	Histone Demethylase	15
LLY-507	Histone Methyltransferase	15
AGK2	Sirtuin	15
GSK J1	Histone Demethylase	15
Anacardic Acid	Histone Acetyltransferase	15
BRD4770	Histone Methyltransferase	15
Splitomicin	HDAC	15
Santacruzamate A (CAY10683)	HDAC	15
CAY10603	HDAC	15
EI1	Histone Methyltransferase	15
CPI-169	Histone Methyltransferase	15
Tasquinimod	HDAC	15
MI-2 (Menin-MLL Inhibitor)	Histone Methyltransferase	15
GSK1324726A (I-BET726)	Epigenetic Reader Domain	15
OF-1	Epigenetic Reader Domain	15
BG45	HDAC	15
BRD73954	HDAC	15
EPZ015666 (GSK3235025)	Histone Methyltransferase	15
SRT2104 (GSK2245840)	Sirtuin	15
ORY-1001 (RG-6016) 2HCl	Histone Demethylase	15

GSK2879552 2HCl	Histone Demethylase	15
GSK503	Histone Methyltransferase	15
MI-136	Histone Methyltransferase	15
MI-463	Histone Methyltransferase	15
EPZ020411 2HCl	Histone Methyltransferase	15
SGC707	Histone Methyltransferase	15
OICR-9429	Histone Methyltransferase	15
I-BRD9	Epigenetic Reader Domain	15
SirReal2	Sirtuin	15
AMI-1	Histone Methyltransferase	15
PFI-4	Epigenetic Reader Domain	15
A-196	Histone Methyltransferase	15
Tenovin-1	p53	15
Ricolinostat (ACY-1215)	HDAC	15
BIX 01294	Histone Methyltransferase	15
Scriptaid	HDAC	15
UNC0638	Histone Methyltransferase	15
GSK591	Histone Methyltransferase	15
MS023	Histone Methyltransferase	15
MS049	Histone Methyltransferase	15
BI-7273	Epigenetic Reader Domain	15
PF-CBP1 HCl	Epigenetic Reader Domain	15
HLCL-61 HCL	Histone Methyltransferase	15
Thiomyristoyl	Sirtuin	15
GSK6853	Epigenetic Reader Domain	15
SRT2183	Sirtuin	15
CPI-455 HCl	Histone Demethylase	15
dBET1	Epigenetic Reader Domain	15
ITSA-1 (ITSA1)	HDAC	15
SGC2085	Histone Methyltransferase	15
CPI-1205	Histone Methyltransferase	15
UNC3866	Histone Methyltransferase	15
CBL0137 (CBL-0137)	NF-κB	15
KG-501 (2-naphthol-AS-E-phosphate)	Epigenetic Reader Domain	15
T-3775440 HCl	Histone Demethylase	15
Salermide	Sirtuin	15
PF-06726304	Histone Methyltransferase	15
WT161	HDAC	15
TMP195	HDAC	15
Tucidinostat (Chidamide)	HDAC	15
JQ-EZ-05 (JQEZ5)	Histone Methyltransferase	15
OSS_128167	Sirtuin	15

3-TYP	Sirtuin	15
ACY-738	HDAC	15
GSK3326595 (EPZ015938)	Histone Methyltransferase	15
INCB057643	Epigenetic Reader Domain	15
dBET6	Epigenetic Reader Domain	15
Tinostamustine (EDO-S101)	HDAC	15
TH34	HDAC	15
WM-1119	Histone Acetyltransferase	15
WM-8014	Histone Acetyltransferase	15
Inauhzin	Sirtuin	15
I-CBP112	Epigenetic Reader Domain	15
KDM4D-IN-1	Histone Demethylase	15
BML-210 (CAY10433)	HDAC	15
SR-4370	HDAC	15
WDR5-0103	Histone Methyltransferase	15
NKL 22	HDAC	15
SIS17	HDAC	15
UBCS039	Sirtuin	15
CM272	Histone Methyltransferase	15
MZ-1	Epigenetic Reader Domain	15
BI 894999	Epigenetic Reader Domain	15
BI-9564	Epigenetic Reader Domain	15
Mocetinostat (MGCD0103)	HDAC	15
SRT1720 HCl	Sirtuin	15

Table S3. Sequences for shRNA and sgRNA.

Gene	Sequence	Species
Control-RNAi	TTCTCCGAACGTGTCACGT	Human
BRD4-RNAi-1	GCCTATGTCCTATGAGGAGAA	Human
BRD4-RNAi-2	CCTATGGATATGGGAACAATA	Human
BRD4-RNAi-3	CAGTGACAGTTCGACTGATGA	Human
PML-RNAi-1	AGGAGCAGGATAGTGCCTTTG	Human
PML-RNAi-2	CACCCGCAAGACCAACAACAT	Human
PML-RNAi-3	GTGTACCGGCAGATTGTGGAT	Human
TIMP1-RNAi-1	GCACAGTGTTTCCCTGTTTAT	Human
TIMP1-RNAi-2	CCAGCGTTATGAGATCAAGAT	Human
TIMP1-RNAi-3	CTGTTGTTGCTGTGGCTGATA	Human
Control-RNAi	TTCTCCGAACGTGTCACGT	Mouse
Timp1-RNAi-1 (AAV-shRNA)	CACAGACAGCCTTCTGCAACT	Mouse
Timp1-RNAi-2 (AAV-shRNA)	CATGGAAAGCCTCTGTGGATA	Mouse

Timp1-RNAi-3 (AAV-shRNA)	GCTCAGCAAAGAGCTTTCTCA	Mouse
sgRNA-no targeting control	CGACTGCGGCACTCGATCTC	Mouse
sgRNA SE1_1	ACTGAGCGAGAGATTGCCCGAGG	Mouse
sgRNA SE1_2	GGCAGAGTCCTGTTGTCGACAGG	Mouse
sgRNA SE1_3	AGTTACAATCCAACTCATCCCGG	Mouse
SgRNA SE2_1	GCGTCTCTTGAATGACCTTGAGG	Mouse
sgRNA SE2_2	TCGTACAGAGGTAGACAACAGGG	Mouse
AAV-sgNC	CGCTTCCGCGGCCCGTTCAA	Mouse
AAV-sg*PML*	TCGTACAGAGGTAGACAACA	Mouse

Table S4. Primer sequences.

Gene	Sequence-Forward	Sequence-Reverse
Primers for human qRT-PCR		
CCL2	CGCTCAGCCAGATGCAAT	CAATGGTCTTGAAGATCACAGC
PML	CTCTGAGCTCTGGCCAACAA	TCAAGCCAGCAAATGACACG
TIMP1	GTCATCAGGGCCAAGTTCGT	TGTTCCAGGGAGCCACAAAA
CXCL1	GGAAAGCTTGCCTCAATCCT	TTGTCACTGTTCAGCATCTTTTCG
BRD4	CAGGCCACTGATGGGTACAG	CTCAAGCACAGTGGCAACAC
STOML1	CCTGGGTGATTTTGACCGCT	GGGCAAACCAGCCAGAAATG
GOLGA6A	AGACGGCATGAGAGAGTCCT	GGCAACACCAACTATTGCAG
GAPDH	ACATCGCTCAGACACCATG	TGTAGTTGAGGTCAATGAAGGG
Primers for mouse qRT-PCR		
Pml	CTAATGTAGTTTGTATGGGCTTTGCA	CCTGACTTATGAGTAATCTTGAAGTGG
Brd4	TGGCAGAAGCTCTGGAGAAG	TGCCCCTGTTTCTTTCCTCC
Timp1	TCATGGGTTCCCCAGAAATCA	GGACCTGATCCGTCCACAAA
β-actin	TGACGTTGACATCCGTAAAG	GAGGAGCAATGATCTTGATCT
Primers for mouse genotyping-PCR		
Brd4	GGATTTCCATAGGTCTTCATTTGCT	CAGAGGAGAGCATGAAGATATGTTCC
Pml	AGCGCCTACCTCAGATGAAAGAC	CTTAGCAGGCACTTTGGTCTGAG
dCas9-KRAB	GCAGCCTCTGTTCCACATACAC AAAGTCGCTCTGAGTTGTTAT	TAAGCCTGCCCAGAAGACTC
Cdh5^{CreERT2}	GTCCTGATGGTGCCTATCCTCTTTC	AATCGCGAACATCTTCAGGTTCTG
Primers for sgRNA		
sgRNA-*PML* SE1_1	CACCAGTACTCTCGCCCAAACCCCAGG	AAACCCTGGGGTTTGGGCGAGAGTACT
sgRNA-*PML* SE1_2	CACCGAGGGAAGTTGCGGTGCCACAGG	AAACCCTGTGGCACCGCAACTTCCCTC
sgRNA-	CACCGAACACAATCATCCGCTC	AAACCCTACGAGCGGATGATTGT

PML SE2_1	GTAGG	GTTC
sgRNA-*PML* SE2_2	CACCCGGATGATTGTGTTCCGCCTGGG	AAACCCCAGGCGGAACACAATCATCCG
sgRNA-*PML* SE3_1	CACCGCGGTTAGGCTGAACAGCGTGGG	AAACCCCACGCTGTTCAGCCTAACCGC
sgRNA-*PML* SE3_2	CACCTCAGCCTAACCGCCTAACCTTGG	AAACCCAAGGTTAGGCGGTTAGGCTGA
sgRNA-*PML* SE4_1	CACCCCCGCTTTACCGTAAGTCAGCGG	AAACCCGCTGACTTACGGTAAAGCGGG
sgRNA-*PML* SE4_2	CACCCGGATGAATGGATCAAAGCCGGG	AAACCCCGGCTTTGATCCATTCATCCG

Table S5. Antibody list.

Antibody	Application	Dilution	Source	Company and cat no
Primary antibody				
αSMA	IF	1:200	Rabbit	Abcam, 124964
	WB	1:2000		
Collagen I	IF	1:100	Goat	Southern Biotech, 1310-01
	WB	1:1000	Rabbit	Abcam, 260043
F4/80	IF	1:100	Rabbit	Cell Signaling, 70076
	DAOSLIMIT	3 μg per mouse	Rat	Biolegend, 111604
MPO	IF	1:100	Rat	Abcam, 90810
HSC70	WB	1:1000	Rabbit	Santa Cruz Biotechnology, Sc-7298
BRD4	WB	1:1000	Rabbit	Abcam, 128874
	Co-IP	1:100	Rabbit	Cell Signaling, 83375
	ChIP-seq	5 μg/mg lysate	Rabbit	Bethyl Labs, A301-985A50
PML	WB	1:1000	Mouse	Santa Cruz Biotechology, sc-71910
	Co-IP	1:100	Rabbit	Abcam, 179466
	ChIP-seq	5 μg/mg lysate	Mouse	Santa Cruz Biotechology, sc-71910
H3K27ac	ChIP-seq	2 μg/ mg lysate	Rabbit	Abcam, 4729
CD63	IF	1:100	Rabbit	Abcam, 217345
	DAOSLIMIT	5 μg per mouse	Rat	Biolegend, 143906
CD63	IF	1:100	Mouse	Abcam, 193349
CD68	IF	1:100	Mouse	Santa, sc-20060
CD166	IF	1:100	Rabbit	Abcam, 109215
	DAOSLIMIT	5 μg per mouse		

Ly6G	DAOSLIMIT	5 μg per mouse	Rat	Biolegend, 127610
WGA	DAOSLIMIT	5 μg per mouse	Wheat	Thermo Fisher, W11261
LYVE1	IF	1:400	Goat	R&D, AF2089
LYVE1	IF	1:400	Goat	R&D, AF2125
PML	IF	1:50	Rabbit	Abcam, 67761
CD31	IF	1:100	Rabbit	Cell Signaling, 77699
GST	WB	1:4000	Rabbit	Abcam, 111947
GAPDH	WB	1:10000	Rabbit	ABclonal, A19056
CD45	FC	1 μl per mouse	Rat	BioLegend, 103128
CD11b	FC	1 μl per mouse	Rat	BioLegend, 101205
Ly6c	FC	1 μl per mouse	Rat	BioLegend, 128033
F4/80	FC	1 μl per mouse	Rat	BioLegend, 123141
Cd63	FC	1 μl per mouse	Rat	BioLegend, 143904
Secondary antibody				
HRP-conjugated Goat anti-Rabbit IgG (H+L)	WB	1:10000	Goat	Zhongshanjinqiao，ZB-2301
HRP-conjugated Goat anti-Mouse IgG (H+L)	WB	1:10000	Goat	Zhongshanjinqiao，ZB-2305
Dnk pAb to Rabbit IgG (Alexa Fluor® 488)	IF	1:500	Donkey	Abcam, 150065
Dnk pAb to Rabbit IgG (Alexa Fluor® 555)	IF	1:500	Donkey	Abcam, 150062
Dnk pAb to Rabbit IgG (Alexa Fluor® 647)	IF	1:500	Donkey	Abcam, 150063
Dnk pAb to Mouse IgG (Alexa Fluor® 488)	IF	1:500	Donkey	Abcam, 150105
Dnk pAb to Mouse IgG (Alexa Fluor®	IF	1:500	Donkey	Abcam, 150110

555)				
Dnk pAb to Mouse IgG (Alexa Fluor® 647)	IF	1:500	Donkey	Abcam, 150107
Dnk pAb to Rat IgG (Alexa Fluor® 488)	IF	1:500	Donkey	Abcam, 150153
Dnk pAb to Rat IgG (Alexa Fluor® 555)	IF	1:500	Donkey	Abcam, 150154
Dnk pAb to Rat IgG (Alexa Fluor® 647)	IF	1:500	Donkey	Abcam, 150155
Dnk pAb to Goat IgG (Alexa Fluor® 488)	IF	1:500	Donkey	Abcam, 150129
Dnk pAb to Goat IgG (Alexa Fluor® 555)	IF	1:500	Donkey	Abcam, 150130
Dnk pAb to Goat IgG (Alexa Fluor® 647)	IF	1:500	Donkey	Abcam, 150131
