# Supplementary material for: Epigenetically controlled endothelial promyelocytic leukemia drives liver inflammation and fibrosis
Source: J Clin Invest. 2026 Mar 17;136(11):e196730. doi: 10.1172/JCI196730 (PMC13221223; doi:10.1172/JCI196730)
Supplement: Unedited blot and gel images [file jci-136-196730-s111.pdf]

Full unedited gel

Full unedited gel for Figure 1K

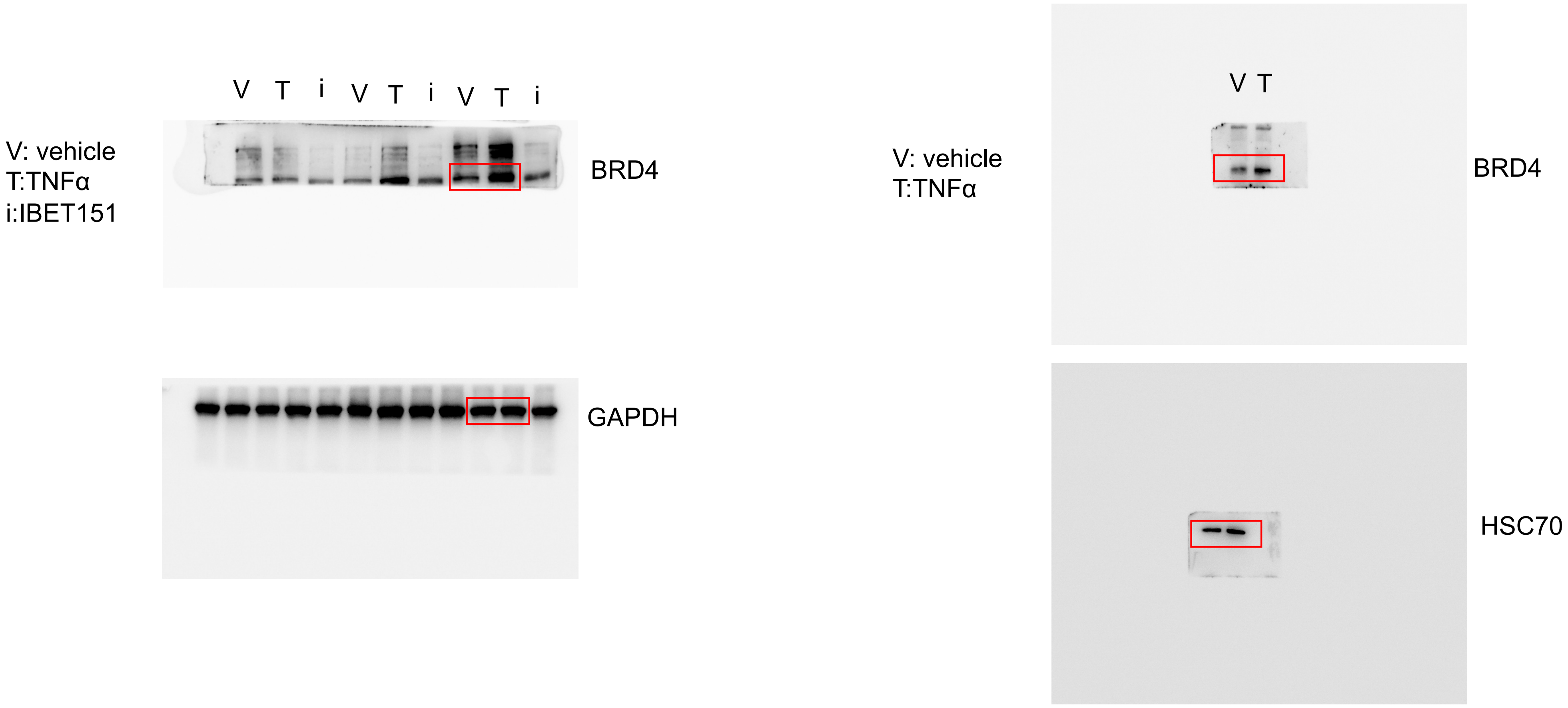

Full unedited gel for Figure 2H

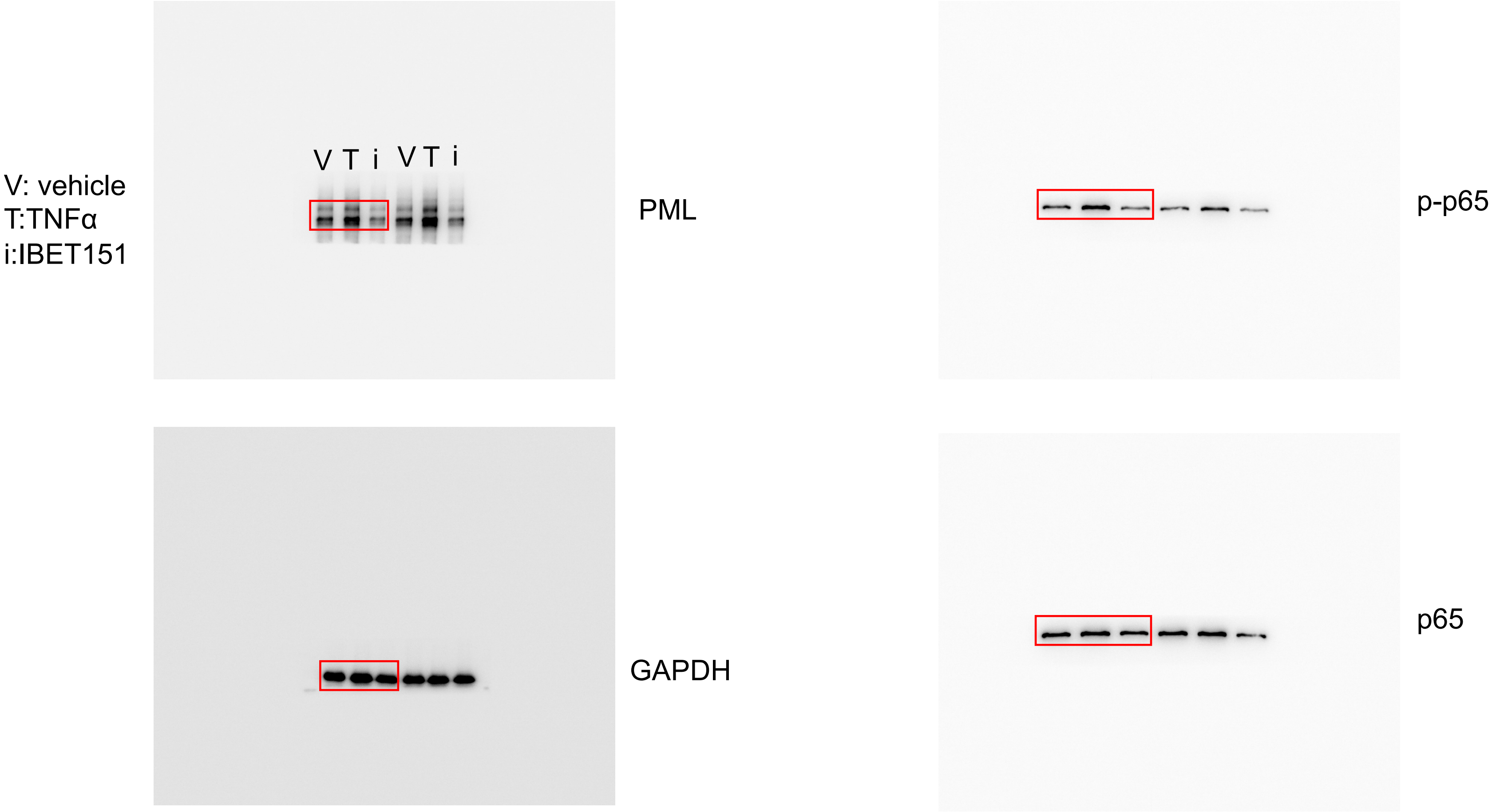

Full unedited gel for Figure 3B

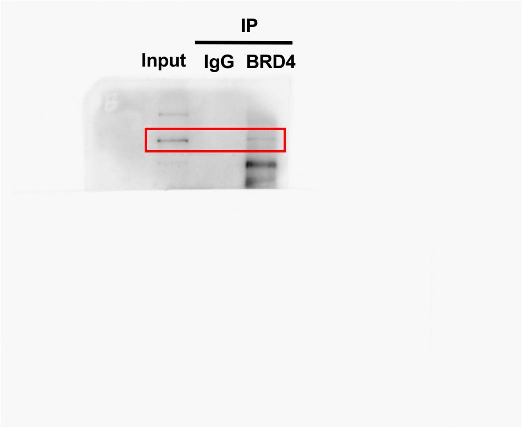

BRD4

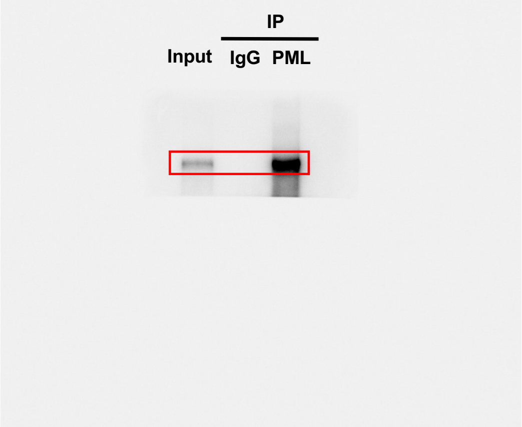

PML

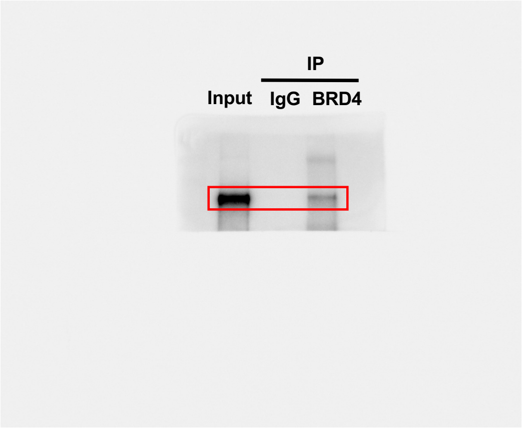

PML

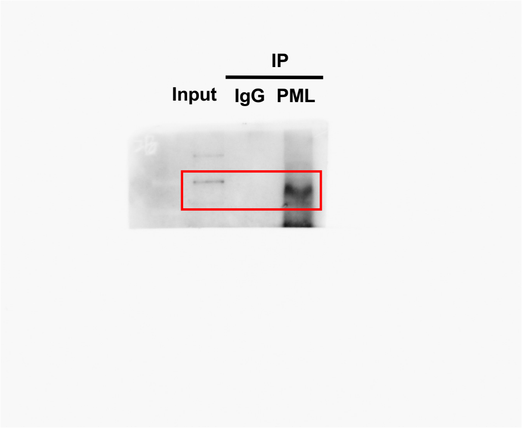

BRD4

Full unedited gel for Figure 3D

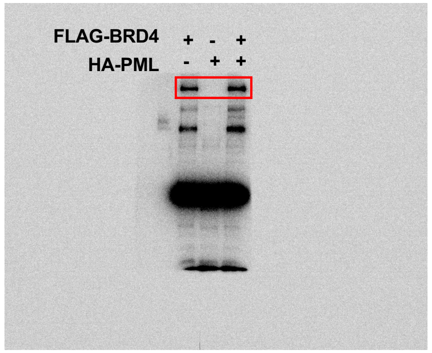

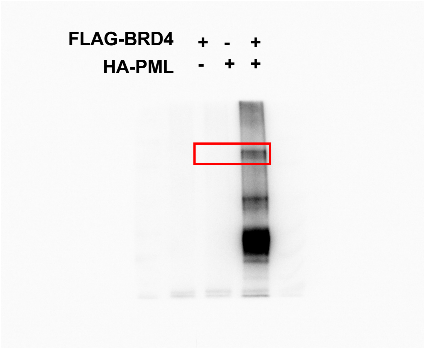

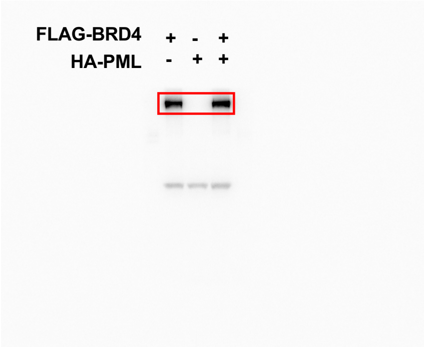

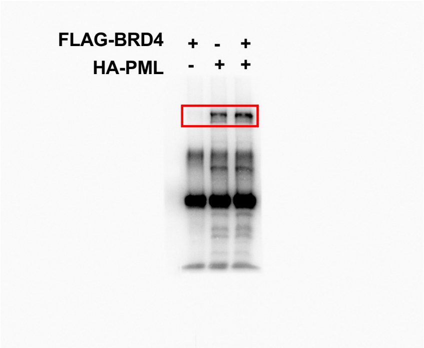

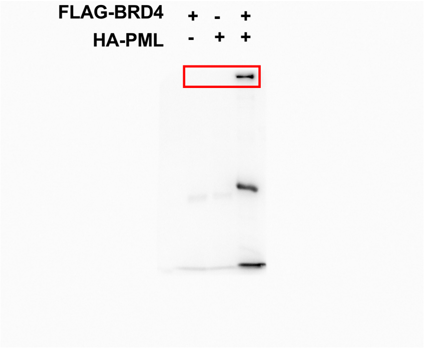

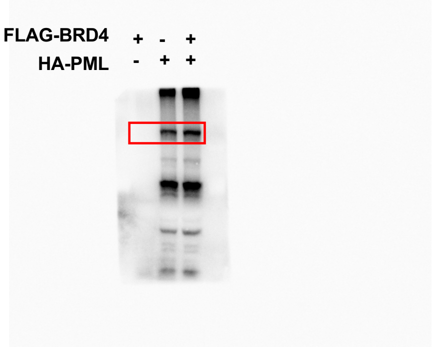

Full unedited gel for Figure 3G and Figure S5C

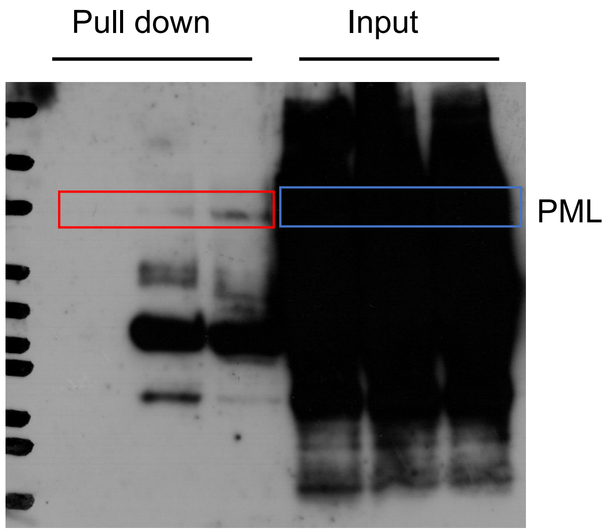

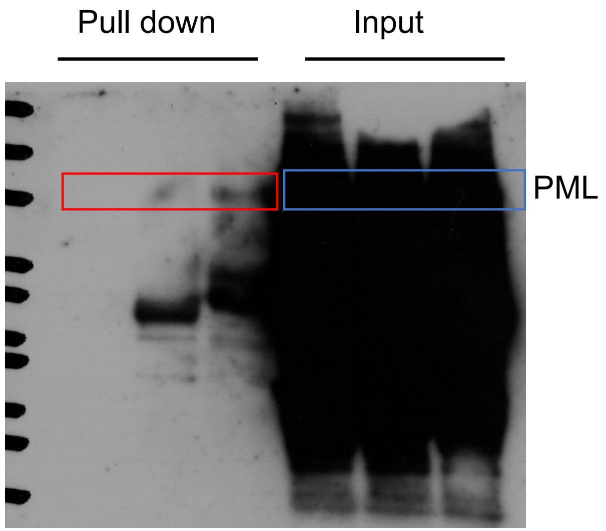

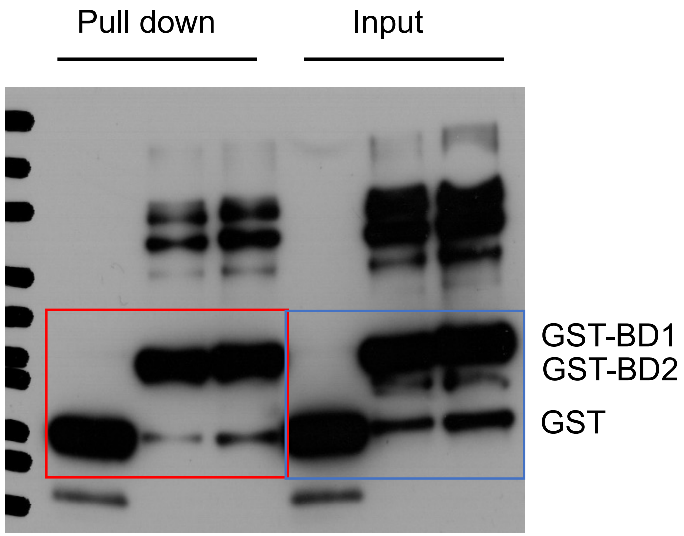

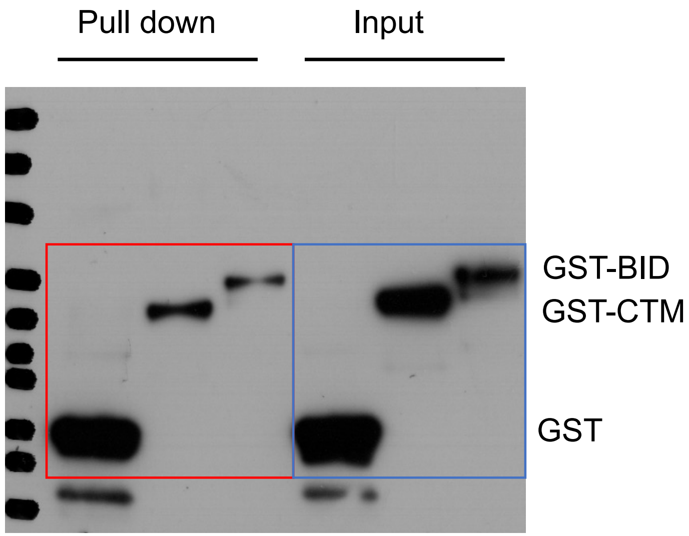

Full unedited gel for Figure 3I and Figure S5D

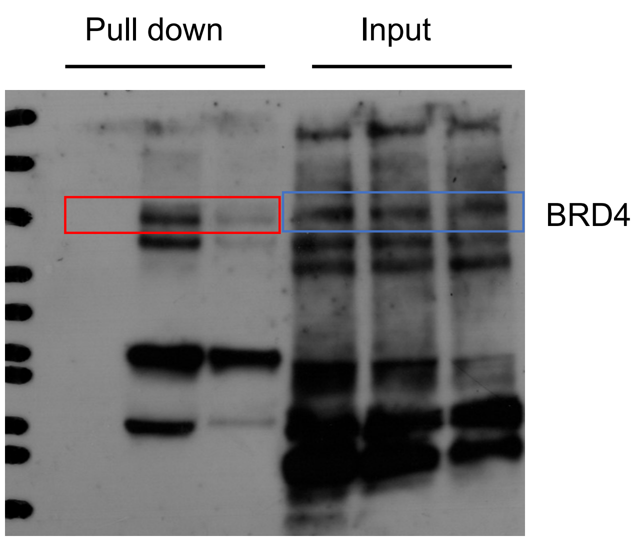

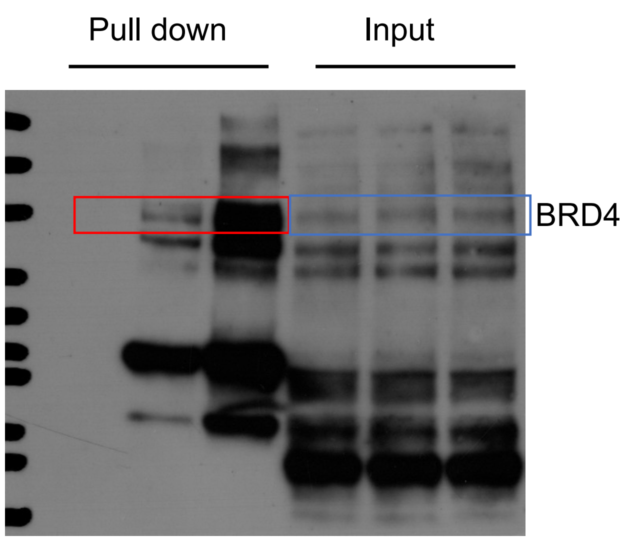

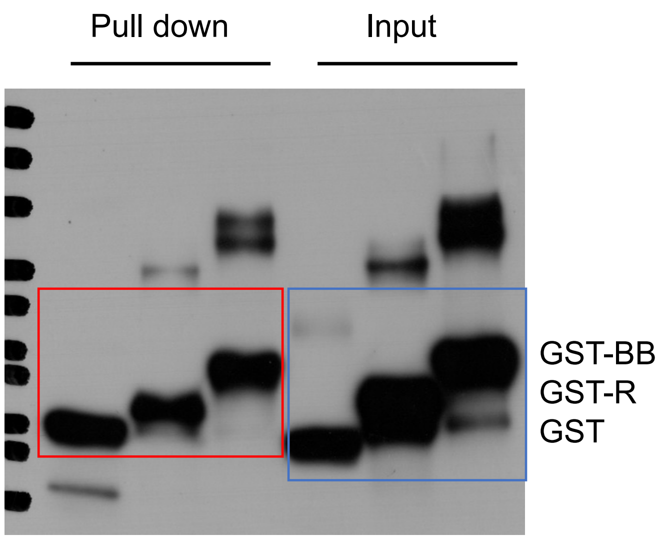

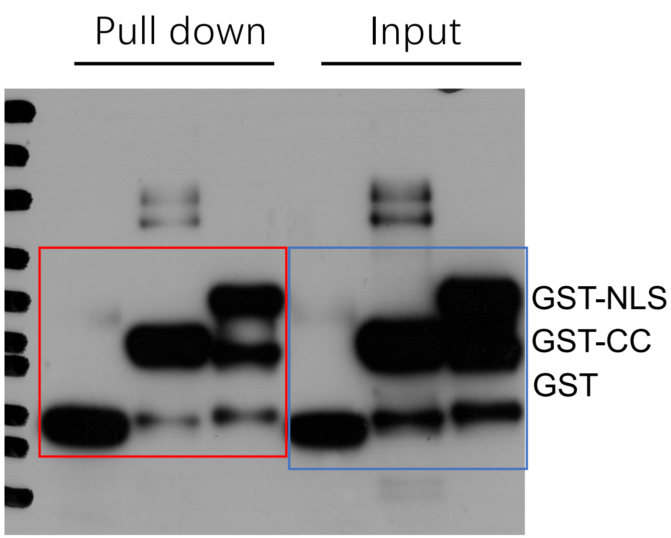

Full unedited gel for Figure 4E

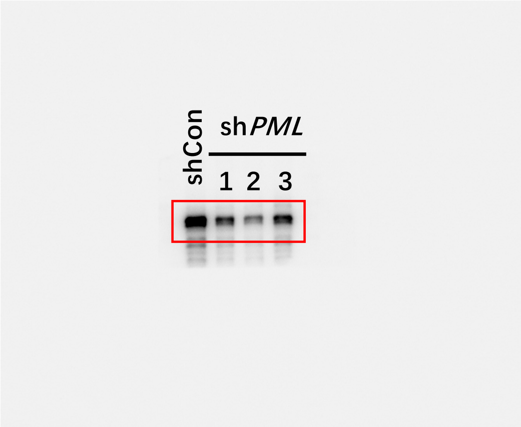

PML

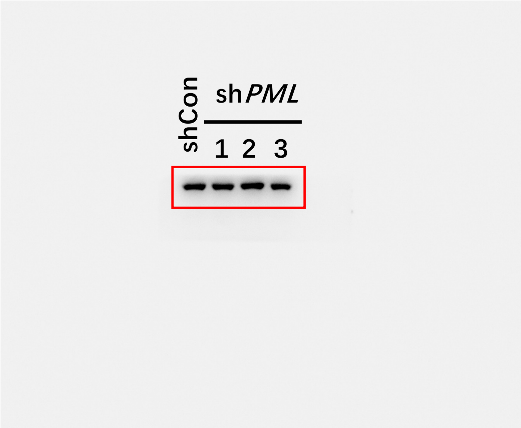

HSC70

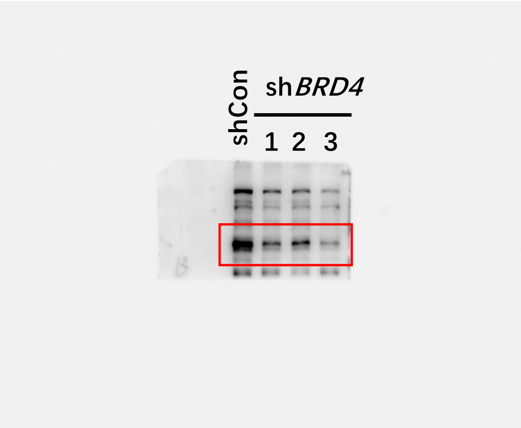

BRD4

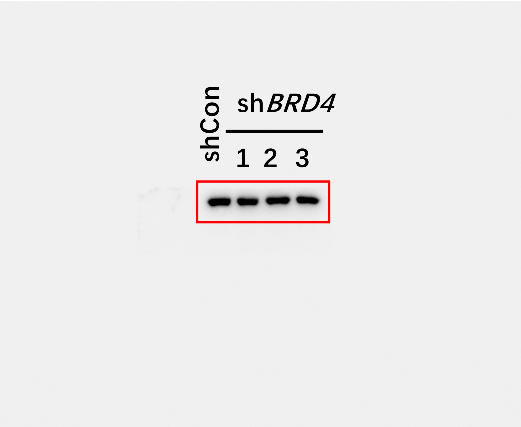

HSC70

Full unedited gel for Figure 5B

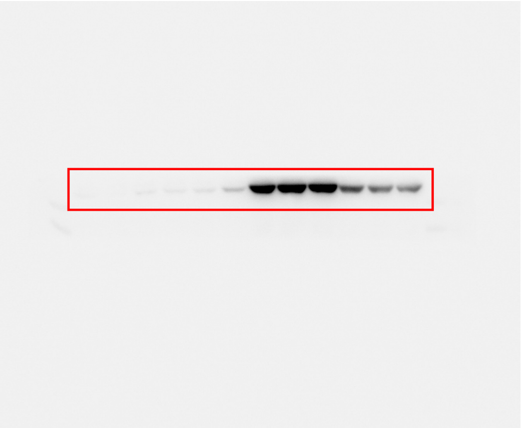

αSMA

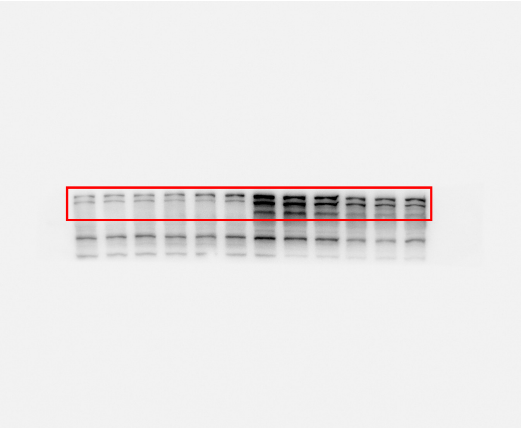

Collagen I

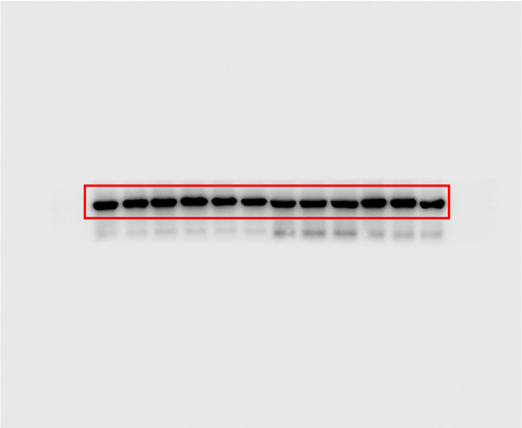

HSC70

Full unedited gel for Figure 5D

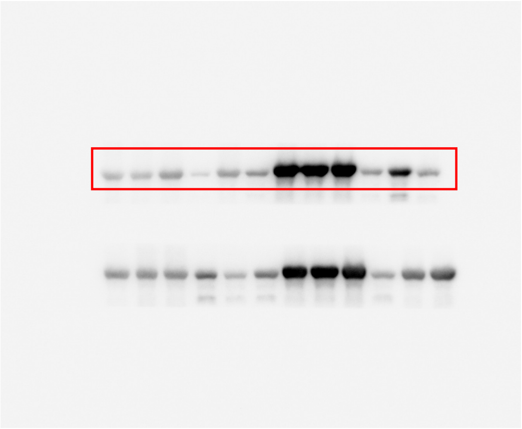
αSMA

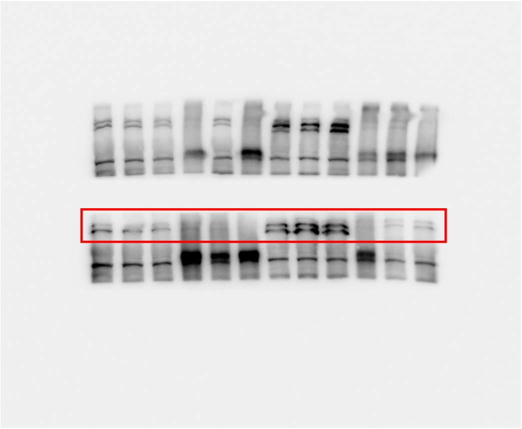
Collagen I

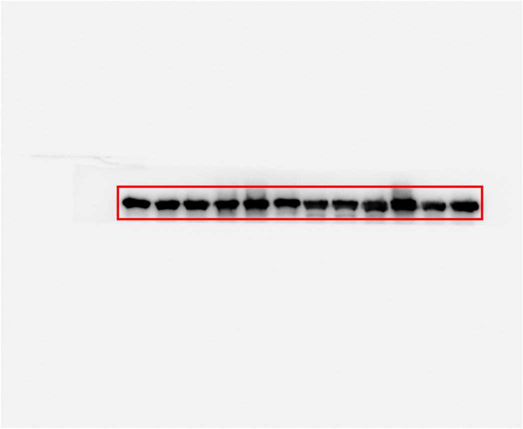
HSC70

Full unedited gel for Figure 7F

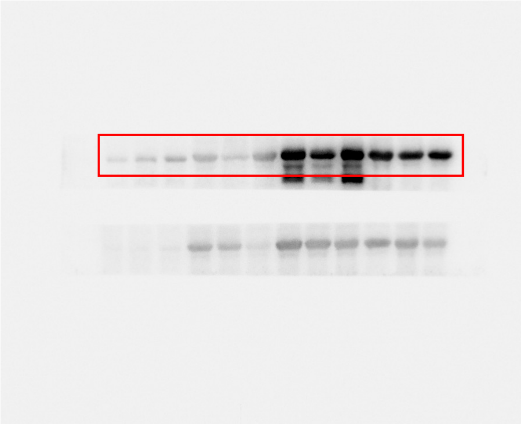

αSMA

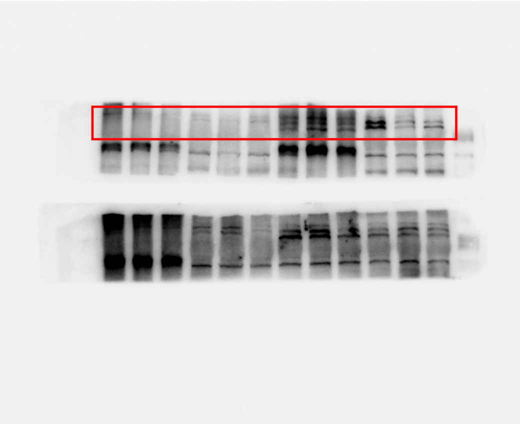

Collagen I

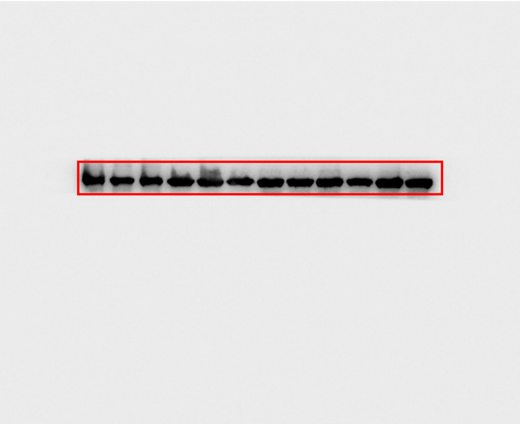

HSC70

Full unedited gel for Figure 8C

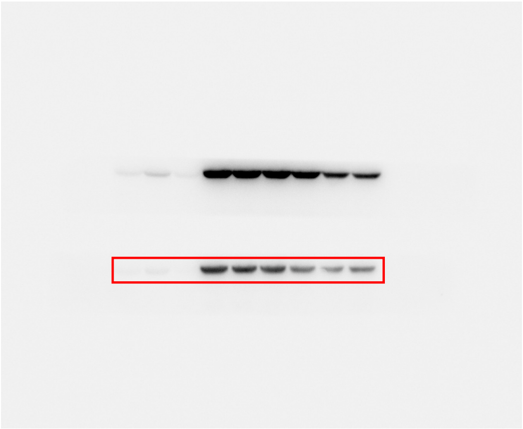

αSMA

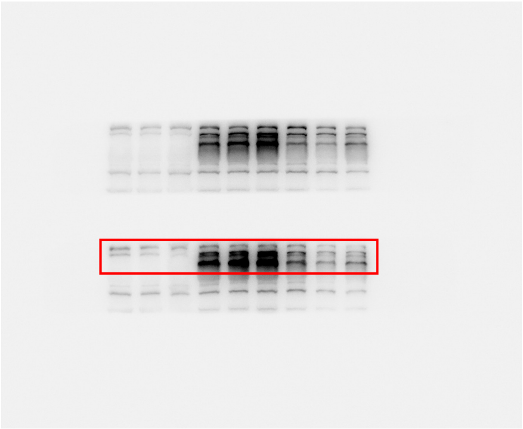

Collagen I

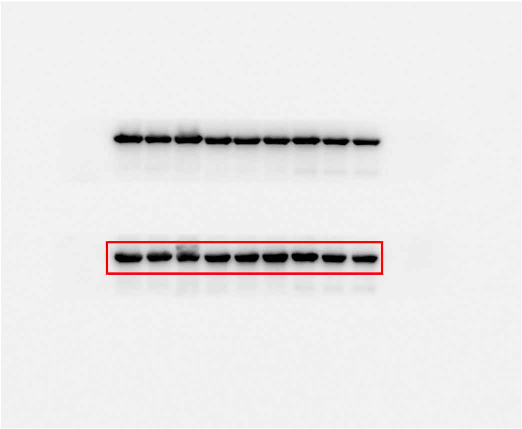

HSC70

Full unedited gel for Figure 8F

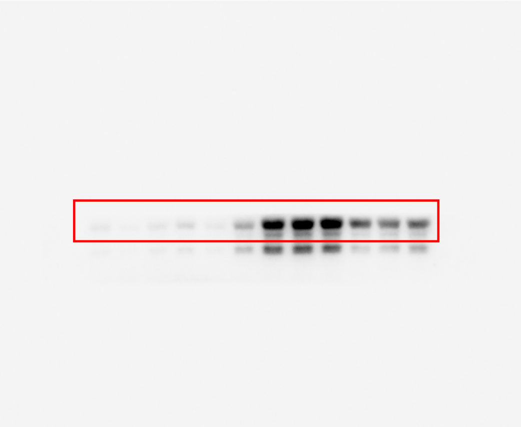

αSMA

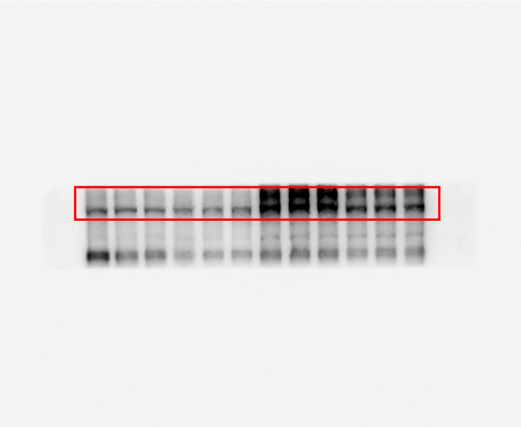

Collagen I

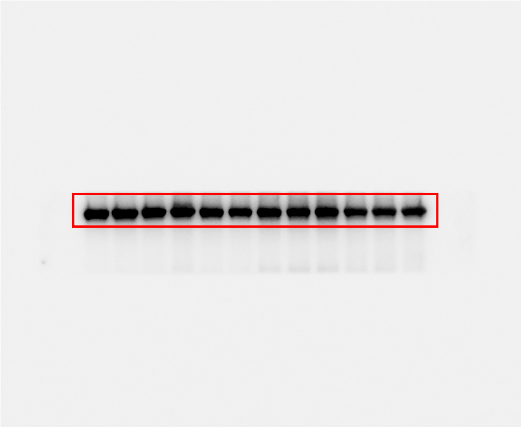

HSC70

Full unedited gel for Figure S2F

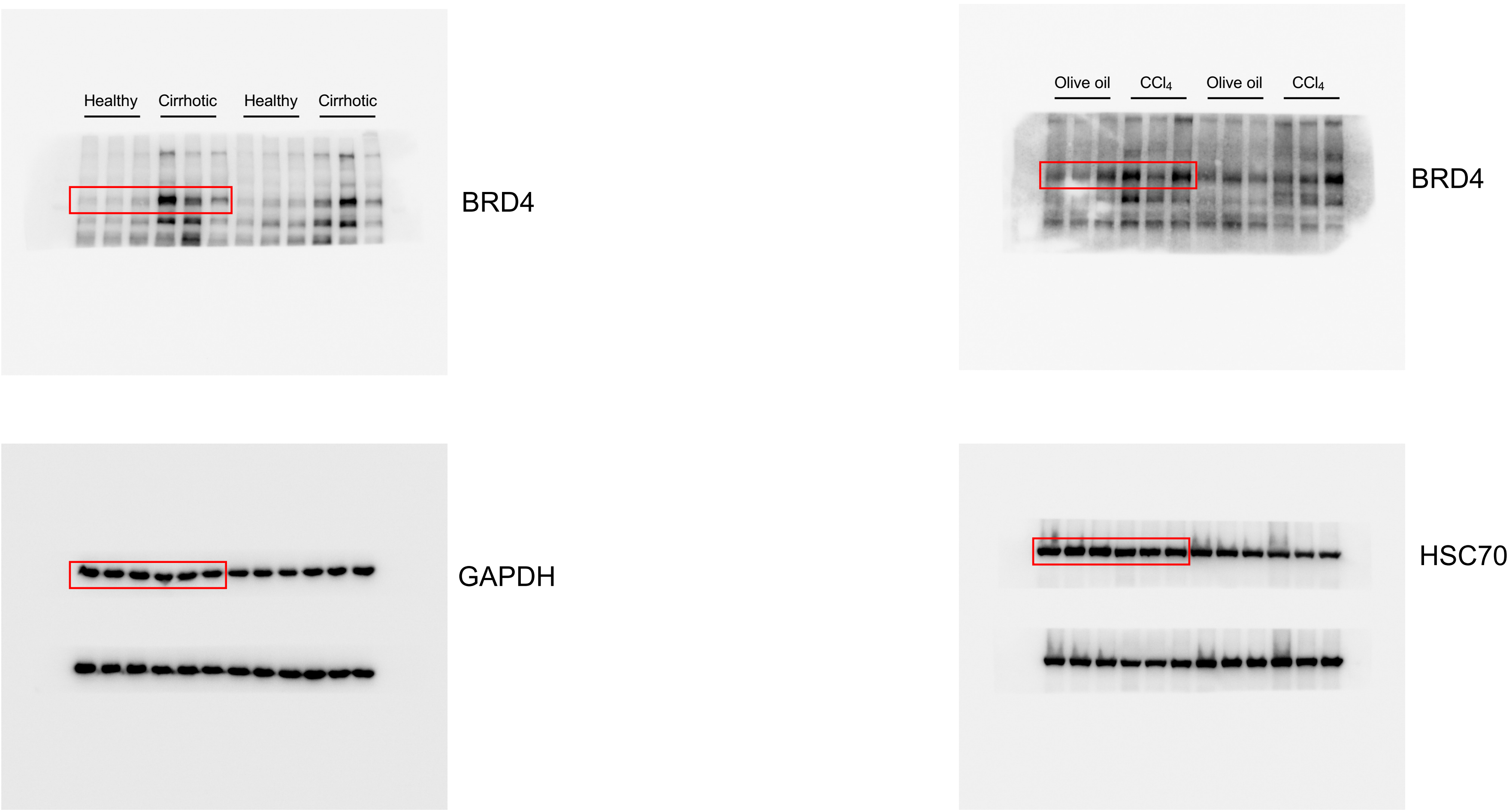

Full unedited gel for Figure S5A

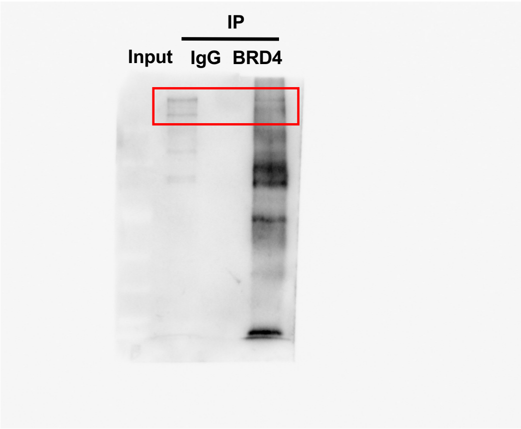

BRD4

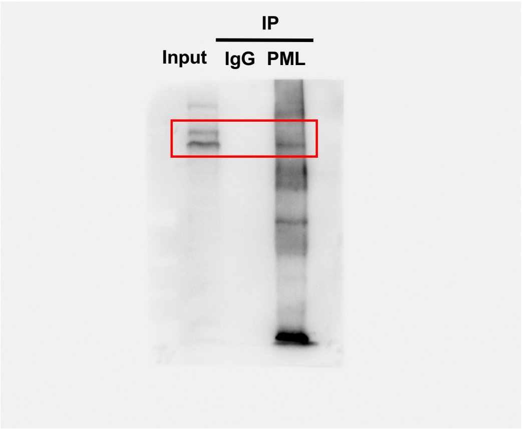

PML

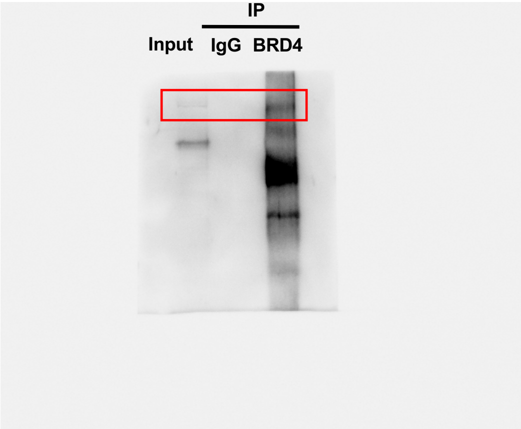

PML

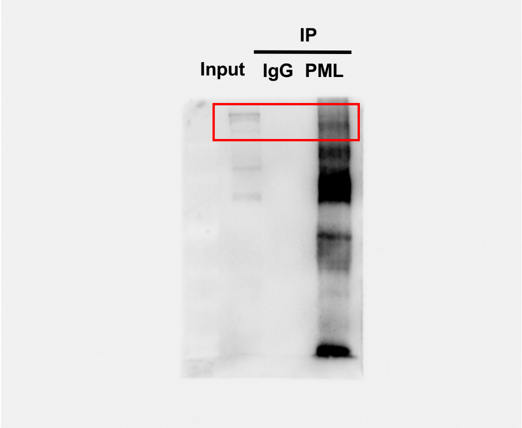

BRD4

Full unedited gel for Figure S11D

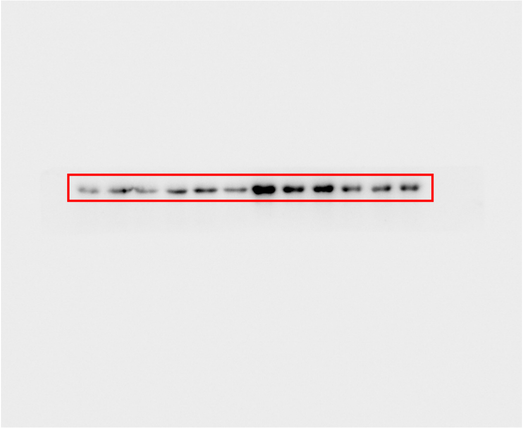

αSMA

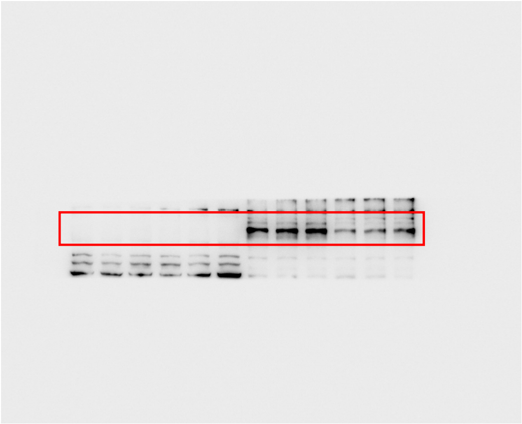

Collagen I

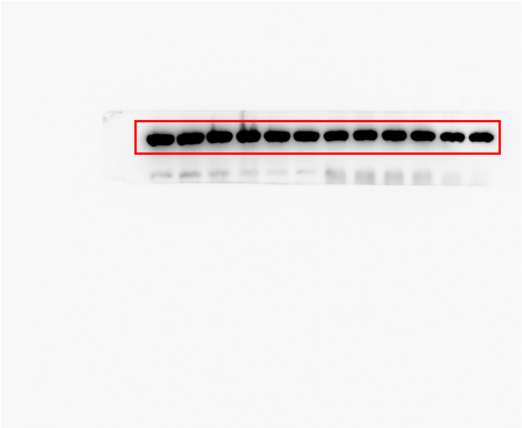

HSC70

Full unedited gel for Figure S12D

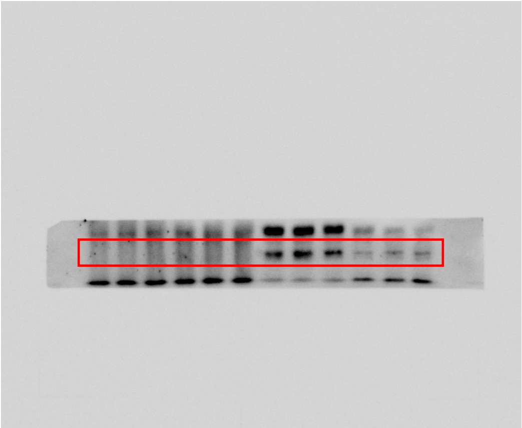

αSMA

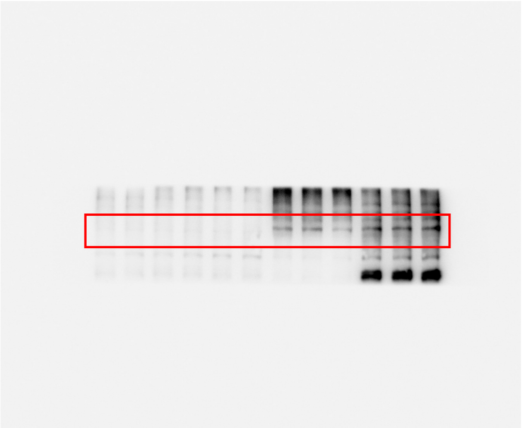

Collagen I

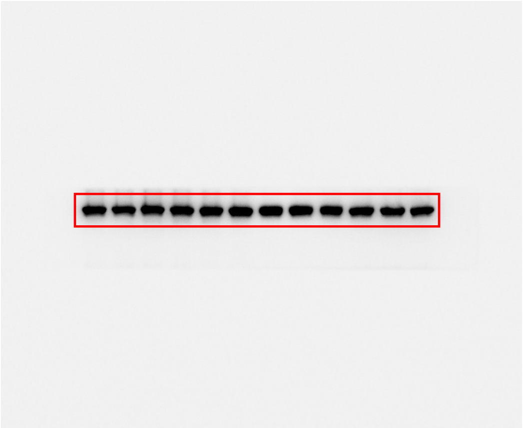

HSC70

Full unedited gel for Figure S20G

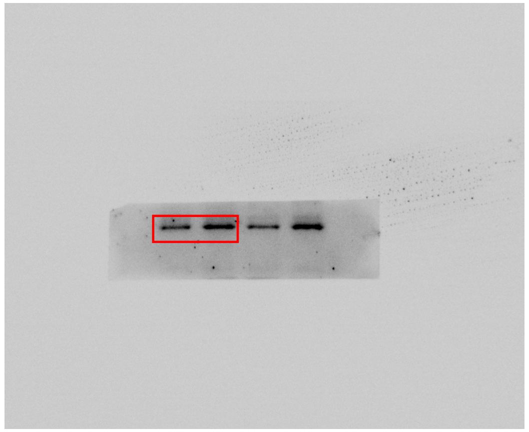

αSMA

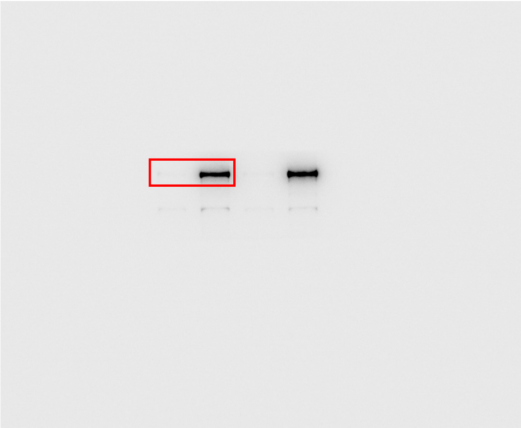

Collagen I

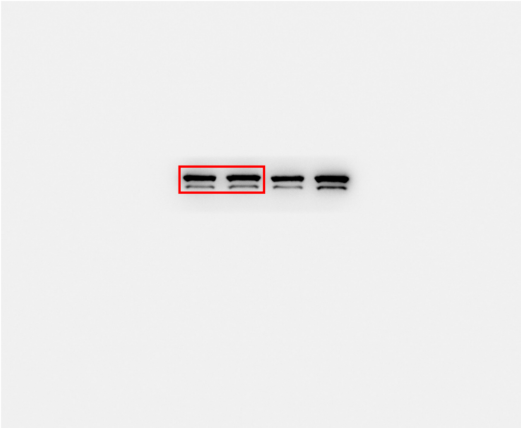

HSC70

Full unedited gel for Figure S20J

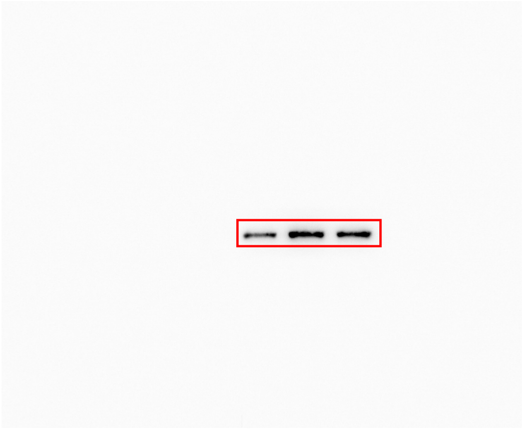

αSMA

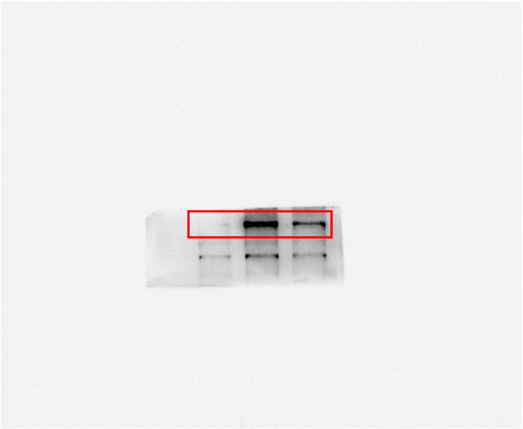

Collagen I

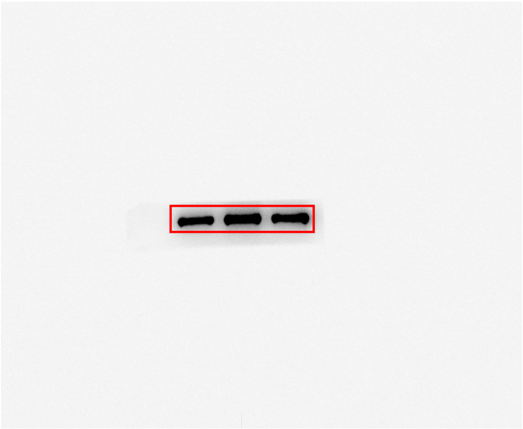

HSC70
